# VariantSpark: Cloud-based machine learning for association study of complex phenotype and large-scale genomic data

**DOI:** 10.1093/gigascience/giaa077

**Published:** 2020-08-06

**Authors:** Arash Bayat, Piotr Szul, Aidan R O’Brien, Robert Dunne, Brendan Hosking, Yatish Jain, Cameron Hosking, Oscar J Luo, Natalie Twine, Denis C Bauer

**Affiliations:** Health and Biosecurity, Commonwealth Scientific and Industrial Research Organisation (CSIRO), 11 Julius Ave North Ryde NSW 2113 Australia; Data61, Commonwealth Scientific and Industrial Research Organisation (CSIRO), 5 Garden St Eveleigh NSW 2015 Australia; Health and Biosecurity, Commonwealth Scientific and Industrial Research Organisation (CSIRO), 11 Julius Ave North Ryde NSW 2113 Australia; Data61, Commonwealth Scientific and Industrial Research Organisation (CSIRO), 5 Garden St Eveleigh NSW 2015 Australia; Health and Biosecurity, Commonwealth Scientific and Industrial Research Organisation (CSIRO), 11 Julius Ave North Ryde NSW 2113 Australia; Health and Biosecurity, Commonwealth Scientific and Industrial Research Organisation (CSIRO), 11 Julius Ave North Ryde NSW 2113 Australia; Health and Biosecurity, Commonwealth Scientific and Industrial Research Organisation (CSIRO), 11 Julius Ave North Ryde NSW 2113 Australia; Department of Systems Biomedical Sciences, School of Medicine, Jinan University, 601 Huangpu Ave, Guangzhou, Guangdong Province, China; Health and Biosecurity, Commonwealth Scientific and Industrial Research Organisation (CSIRO), 11 Julius Ave North Ryde NSW 2113 Australia; Health and Biosecurity, Commonwealth Scientific and Industrial Research Organisation (CSIRO), 11 Julius Ave North Ryde NSW 2113 Australia; Department of Biomedical Sciences, Macquarie University NSW 2109 Australia

## Abstract

**Background:**

Many traits and diseases are thought to be driven by >1 gene (polygenic). Polygenic risk scores (PRS) hence expand on genome-wide association studies by taking multiple genes into account when risk models are built. However, PRS only considers the additive effect of individual genes but not epistatic interactions or the combination of individual and interacting drivers. While evidence of epistatic interactions ais found in small datasets, large datasets have not been processed yet owing to the high computational complexity of the search for epistatic interactions.

**Findings:**

We have developed VariantSpark, a distributed machine learning framework able to perform association analysis for complex phenotypes that are polygenic and potentially involve a large number of epistatic interactions. Efficient multi-layer parallelization allows VariantSpark to scale to the whole genome of population-scale datasets with 100,000,000 genomic variants and 100,000 samples.

**Conclusions:**

Compared with traditional monogenic genome-wide association studies, VariantSpark better identifies genomic variants associated with complex phenotypes. VariantSpark is 3.6 times faster than ReForeSt and the only method able to scale to ultra-high-dimensional genomic data in a manageable time.

## Findings

Traditional genome-wide association studies (GWAS) evaluate genomic variants (referred to hereinafter as “variants") across the genomes of many samples for statistical association with the phenotype in question. These studies are aimed at detecting variants associated with common and complex traits and diseases, such as heart disease, diabetes, and height [1]. GWAS has been successful at identifying >50,000 associated variants in thousands of complex phenotypes (GWAS catalog [2]). Yet, many phenotypes with genetic components remain only partially explained by genetics, the so-called missing heritability problem [3].

One possible explanation is that these phenotypes are driven by an additive effect of several variants (“polygenic phenotype"), resulting in a small association power for each variant [4–7]. Polygenic risk score (PRS) takes into account this additive effect to compute the genomic risk factor for a trait [8]. PRS refers to a range of statistical methods that consider the GWAS association power as a weight for the variant. Given the weight and the risk allele for each variant, PRS computes the genomic risk factor for a given sample [9]. For many phenotypes, PRS is shown to be a more accurate predictor of risk than single variants alone [10], which statistically supports the idea of the phenotype being polygenic.

Another explanation is the existence of epistatic interaction (referred to hereinafter as “interaction") between sets of variants [11] (“epistatic phenotype"). In an interaction, the combination of ≥2 variants highly correlate with the phenotype but individual variants do not show a strong correlation with the phenotype. Thus the phenotype cannot be explained by the individual variants. Variant interactions remain invisible to traditional GWAS and subsequently to PRS methods. Several algorithms have been developed to speed up the search for the interactions [12,13], and they have been successful to identify significant statistical interactions [14]. There is also evidence that interactions are biologically relevant [15]. However, the high computational complexity of these methods prevents them from being applied to whole-genome data. Pruning the dataset is an option but does not guarantee to preserve all the interacting variants.

Given that there is statistical proof for the existence of both polygenic phenotype and epistatic phenotype, there is a likelihood of a complex phenotype to exist—a phenotype that is driven by several variants individually as well as several sets of interactive variants. A novel association approach is hence needed to take into account the individual variant association power, as well as the association power driven by the interactive variants. Furthermore, such a methodology needs to be applicable to genomic-scale data. Taking all variants into account reduces the chance of missing important interactions. Note that the association of interactive variants is only visible when all of them are combined. The computational complexity of such analyses made them infeasible in the past; however, combining more efficient algorithms with parallel computing resources has opened up a new avenue.

One promising algorithm to use is random forest (RF) [16], which is a machine learning approach used in many modern bioinformatics analyses [17] including genomics [18–20]. It is designed to identify interactions between the given features (variants in the context of GWAS) and incorporate them into a prediction model. RF also computes a metric for each variant called “importance score" that is an indicator of the association power for a variant. Importance score combines individual and interaction association power into a single value. Thus RF is a perfect candidate for the association study of a complex phenotype. The randomness in the RF model is the key to avoid over-fitting, making it a robust method. Unlike black box models such as deep learning [21], the RF model is readable and can be used to extract important rules and identify interactive features. Even though RF is not a deterministic algorithm, it is an accurate approximation with a manageable computational requirement.

There are 2 layers of parallelization to speed up an algorithm: multi-threading and distributed computing. The former is a common approach that allows programmers to use all processors and memory available in a single computer, usually a high-performance computer (HPC). The latter allows a program to be executed in parallel on multiple independent computers connected by a network (known as a computer cluster, referred to hereinafter as a “cluster"). Given that the network is far slower than processors and memory, it is critical to implement the program in a way that reduces network operation and avoid a potential bottleneck. Apache Spark [22] (referred to hereinafter as “Spark") is a widely used platform for distributed computing. Distributed computing is a potential solution [23] to overcome the ever-increasing quantity of genomic data, exceeding astronomical data in volume [24].

Here, we introduce VariantSpark, a Spark-based software package for association study of complex phenotypes and genomic-scale datasets. VariantSpark is the first publicly available distributed implementation of RF with the following features to reduce networking, to maximize resource utilization, and to suit genomic datasets:

- Vertical data partitioning
- Processing multiple nodes of multiple trees in parallel
- Efficiently storing genomic data in fast and a low-level Spark memory structure (Resilient Distributed Dataset [RDD])

The wider VariantSpark software suite implements k-means clustering and is compatible with standard genomic data formats (e.g., VCF), and is integrated with Hail [25] to offer a range of other standard genomic analyses in a distributed manner. To assess VariantSpark’s capability we compare it against the state-of-the-art bioinformatics implementation of RF, as well as the latest application-agnostic distributed implementations of RF. Ranger [26] is one of the fastest multi-threaded RFs, written in C++. As reported by its developer, Ranger is 180 times faster than the parallel version of the widely used randomForest R package [27] and requires 3.5 times less memory. It is also 2.2 and 2.6 times faster than randomForestSRC [28] and Random-Jungle [29], respectively. Ranger also implements a save-memory mode that is 1.6 times slower than normal mode but requires half the memory. To the best of our knowledge, no other multi-threaded RF claimed to be faster than Ranger. Despite this, processing data from whole-genome sequencing [30] remains practically impossible using this method. RF needs to maintain the complete dataset decompressed in memory. So a dataset of 100 million variants and 10,000 samples requires 1 TB of memory (assuming 1 byte per genotype), which is unlikely available on standard HPC.

A cluster, on the other hand, can easily scale to hold hundreds of terabytes of data (as most cloud providers can supply). The most popular distributed implementation of RF is Google’s PLANET [31], which is integrated into the Spark machine learning library (MLlib) [32]. PLANET uses horizontal partitioning, which is a parallelization along the wrong dimension because it does not allow high-dimensional data to be loaded into memory as required for random access by the RF algorithm. PLANET is faster than the randomForest R package, with comparisons to other implementations provided in Bayat et al. [33]. ReForeSt [34] is, to the best of our knowledge, the fastest distributed implementation of RF and is up to 3 times faster than MLlib (PLANET). ReForeSt uses similar partitioning as in Spark MLl, extends a machine learning benchmark study [35], and was shown to be faster than XGBOOST [36] and H2O [37] for the largest dataset in the study (10M) [38]. Parallel Random Forest (PRF) [39] is another distributed RF that takes a vertical partitioning approach and claims to be twice as fast as MLlib. The implementation has not been released and hence could not be included in our comparison. The only other relevant distributed algorithm that also implements vertical partitioning relevant for high-dimensional data is Yggdrasil [40]. Yet, Yggdrasil is limited to Decision Tree [41] (DT) and does not expand to build an RF mode. However, none of these tools were tested in ultra-high-dimensional data, which we define as datasets with >10M features.

Here, we first compare the performance of VariantSpark with the approach used in traditional GWAS, logistic regression (LR) [42]. We consider various simulated phenotypes, including complex phenotypes, and different-sized datasets, to compare the tools’ ability to detect associated variants. Then we compare VariantSpark’s runtime with Ranger, ReForeSt, and Yggdrasil. Finally, we demonstrate the scalability of VariantSpark and evaluate sensitivity to hyper-parameter choices.

### Datasets

Two different sets of synthetic datasets are used in this study, both of which are publicly available [43] for the replication of this study (see Supplementary Data File 4). The first set uses real genotypes taken from the 1000-Genomes (1KG) Project [44] and a simulated phenotype made by Polygenic Epistatic Phenotype Simulator (PEPS) [45]. In the second set, both genotype and phenotype are simulated by VariantSpark’s embedded simulator. The phenotype is a function of 5 randomly selected variants and a given noise parameter.

#### Real genotype and simulated phenotype

We use these datasets to compare the accuracy of VariantSpark with LR. A set of phenotypes are simulated for 1KG samples using PEPS that uses real genotype data and simulates a binary phenotype associated with a subset of randomly selected variants.

PEPS first forms *n*-way truth-variables, which are used to simulate the phenotype. A variable could be an individual variant (1-way variable) or set of *n* variants with epistatic interaction (*n*-way variable), so 2-way variables are pairwise epistatic interactions; 3-, 4-, and 5-way variables are higher-order epistatic interactions. Each variant is involved in only 1 variable. Variants involved in truth-variables (associated with the phenotype) are called truth-variants (TVs) and are to be discovered by VariantSpark or LR.

Table 1 lists 9 PEPS simulated phenotypes (provided in Supplementary Data File 3) in 3 categories: PI, PE, and PX. PI phenotypes are made of only 1-way variables (individual variant). PE phenotypes are made of 2-way or higher-order variables (epistatic variables only). PX phenotypes include epistatic and individual variables (complex phenotype). In each category, there are 3 phenotypes with low (L), moderate (M), and high (H) number of TVs.

**Table 1:** Nine phenotypes simulated with PEPS

Phenotype name	Category	No. of *n*-way truth-variables					Total No.	
		1-way	2-way	3-way	4-way	5-way	Truth-variables	Truth-variants
PIL	PI	5	0	0	0	0	5	5
PIM		50	0	0	0	0	50	50
PIH		500	0	0	0	0	500	500
PEL	PE	0	2	2	2	2	8	28
PEM		0	20	20	20	20	80	280
PEH		0	50	50	50	50	200	700
PXL	PX	5	3	2	1	1	12	26
PXM		50	25	17	13	10	115	253
PXH		500	250	167	125	100	1,142	2,501

The 1KG dataset consists of 2,504 samples and ∼80M variants with multi-allelic variants converted to multiple bi-allelic variants. We generate 4 subsets of this dataset by randomly selecting variants, 2 by adding the TVs of all phenotypes back if they were removed by this process (see Table 2).

**Table 2:** 1000-Genome dataset and its subsets

Dataset	No. of variants	% of truth-variants Included
1KG-80M	81,647,203	100
1KG-5M	5,000,516	6.1
1KG-500K	500,446	0.6
1KG-5M-T	5,016,789	100
1KG-500K-T	517,729	100

There are 2,504 samples in these datasets.

#### Simulated Genotype and Simulated Phenotype

These datasets, listed in Table 3, are used for the runtime analysis of VariantSpark. We start from 1,000 samples and 10,000 variants and increase the number of samples or variants 10 times at each step to reach either 100,000 samples and 10,000,000 or 10,000 samples and 100,000,000 variants. These genotypes are simulated with random distribution of phenotypes using the VariantSpark gen-features command.

**Table 3:** Synthetic datasets generated by VariantSpark

Dataset	Size	No.		
		Samples (nS)	Variants (nV)	Genotypes nS × nV
1K-10K	10M	1,000	10,000	1e7
1K-100K	100M	1,000	100,000	1e8
1K-1M	1B	1,000	1,000,000	1e9
1K-10M	10B	1,000	10,000,000	1e10
1K-100M	100B	1,000	100,000,000	1e11
10K-10K	100M	10,000	10,000	1e8
10K-100K	1B	10,000	100,000	1e9
10K-1M	10B	10,000	1,000,000	1e10
10K-10M	100B	10,000	10,000,000	1e11
10K-100M	1T	10,000	100,000,000	1e12
100K-10K	1B	100,000	10,000	1e9
100K-100K	10B	100,000	100,000	1e10
100K-1M	100B	100,000	1,000,000	1e11
100K-10M	1T	100,000	10,000,000	1e12

The phenotype is simulated using VariantSpark gen-label commands and based on 5 randomly selected variants all with equal contributions (all weights are set to 1.0). To make a more complex phenotype, the mean and the standard deviation of the noise, *-*gm and *-*gs parameters, respectively, are both set to 0.5. The fraction of noise variants, *-*gvf parameter, is set to 100/nV to include 100 noise variants (randomly selected from the variants in the dataset).

For the comparison to other tools, we subset variants from the 10K-10M dataset and include the 5 TVs in all subsets. We start from 100 variants and double it at each step. These datasets are listed in Table 4.

**Table 4:** Datasets for high-resolution comparison of the VariantSpark runtime with other implementations of RF

Dataset	No. of variants (nV)	Dataset	No. of variants (nV)
1X	100	512X	51,200
2X	200	1KX	102,400
4X	400	2KX	204,800
8X	800	4KX	409,600
16X	1,600	8KX	819,200
32X	3,200	16KX	1,638,400
64X	6,400	32KX	3,276,800
128X	12,800	64KX	6,553,600
256X	25,600	10M	10,000,000

X represents 100 and KX represents 102,400. 10M is identical to the 10K-10M dataset. Each dataset includes 10,000 samples.

### Compute resources

For reproducibility, all tests are performed on Amazon Web Services (AWS) compute resources. We use AWS EC2 (Elastic Compute Cloud) and EMR (Elastic Map Reduce) for HPC and cluster compute, respectively. We use clusters of different sizes listed in Table 5. For all clusters, the master-node is an r4.2xlarge EC2 instance with 8 virtual central processing unit (vCPU) and 61 GB of memory. Compute-nodes are r4.4xlarge with 16 vCPU and 122 GB of memory except for C256-S and C256-L, where we use r4.2xlare and r4.8xlarge EC2 instances as compute-nodes. The r4.8xlarge has 32 vCPU and 244 GB of memory.

**Table 5:** EMR clusters and compute-nodes

Cluster	Compute-nodes	Master + compute	
		vCPU	Memory (GB)
C16	1 × r4.4xlarge	8 + 16	61 + 122
C32	2 × r4.4xlarge	8 + 32	61 + 244
C64	4 × r4.4xlarge	8 + 64	61 + 488
C128	8 × r4.4xlarge	8 + 128	61 + 976
C256	16 × r4.4xlarge	8 + 256	61 + 1,952
C512	32 × r4.4xlarge	8 + 512	61 + 3,904
C1024	64 × r4.4xlarge	8 + 1,024	61 + 7,808
C256-S	32 × r4.2xlarge	8 + 256	61 + 1,952
C256-L	8 × r4.8xlarge	8 + 256	61 + 1,952

### Experimental set-up

The combination of 9 phenotypes described in Table 1 and 5 genotype datasets described in Table 2 results in 9 × 5 = 45 case/control datasets, which we process with both VariantSpark and LR Wald test implemented in Hail. We pass the first 2 principal component analysis vectors as co-variate to LR. VariantSpark uses the following parameters for this experiment: nTree = 1,000, mTry = 0.1 × nV, maxD = 15, and minNS = 50.

We ranked the variants on the basis of *P*-value computed by LR and importance score computed by VariantSpark. We replicates the experiments 3 times (similar phenotypes are simulated but different randomly selected TVs are used to form the phenotype). In the last 2 replicates, we did not process the 1KG-80M dataset with VariantSpark owing to high computational cost. Owing to technical issues, the VariantSpark results for PIL on 1KG-80M were missed for the first replicate.

VariantSpark and ReForeSt are executed on a C256 cluster while Ranger is executed on r4.16xlarge computer with 64 vCPU and 488 GB of memory, which is the practical limit of HPC. We apply maxD = 15 and minNS = 50 where applicable and build 1,000 trees (nTree = 1,000) with mTry = 0.1 × nV. We build a DT with Yggdrasil 10 times. For VariantSpark we build a forest with 10 trees and set mTry = nV because this parameter setting mimics growing a DT.

When testing VariantSpark’s scalability the following parameters were applied to all experiments below, unless mentioned otherwise: maxD = 15, minNS = 50, mTry = 0.1 × nV, and rbs = 100 (grow 100 trees in parallel).

- Dataset size: The expected runtime to build 1,000 trees for all datasets in Table 3 on C256 cluster. Because the actual runtime is too high for larger datasets, we build fewer trees (i.e., 500, 100, or 10) and record the data load time (β) and average train time per tree (θ) reported by VariantSpark. The expected runtime for 1,000 trees is computed as β + (1,000 × θ). The exact number of trees for each dataset and the un-normalized runtime can be found in Supplementary Data File 1.
- Cluster size: 500 trees are built for 10K-1M dataset on clusters of different sizes (see Table 5). We also replicate this experiment for a 10 times larger dataset (10K-10M) but only on C256, C512, and C1024 clusters.
- Compute-node size: 1,000 trees are built for 10K-1M dataset on C256-S, C256, and C256-L. This experiment is replicated 3 times to show that VariantSpark runtime variation is negligible.
- Batch size (rbs): 500 trees are built for 10K-1M dataset with rbs equal to 10, 50, 100, or 500.

The following experiments are performed to show the effect of different parameters on the VariantSpark runtime and out-of-bag (OOB) error rate (prediction accuracy) when processing the 10K-1M dataset.

- Unlimited: 500 trees are built with no limits on the depth of the tree or the node size.
- Maximum depth (maxD): 500 trees are built with no limits on the node size but the maxD varies as follows: 3, 5, 7, 9, 11, 13, 15, 20, 25, 100.
- Minimum node size (minNS): 500 trees are built with no limits on depth of the trees but the minNS varies as follows: 5, 10, 50, 100, 500, 1,000.
- mTry: 500 trees are built with the mTry varying as follows: 10, 50, 100, 500, 1,000, 5,000, 10,000, 100,000.
- nTree: Starting from 100 trees and doubling the number of trees up to 1,600 trees.

### Result 1: VariantSpark detects complex genomic interactions

We compare the performance of VariantSpark with LR using phenotypes of different complexity and different sized datasets. First, we report how many TVs, i.e., variants associated with the phenotype, can be detected by the respective approaches.

**Figure 1:**
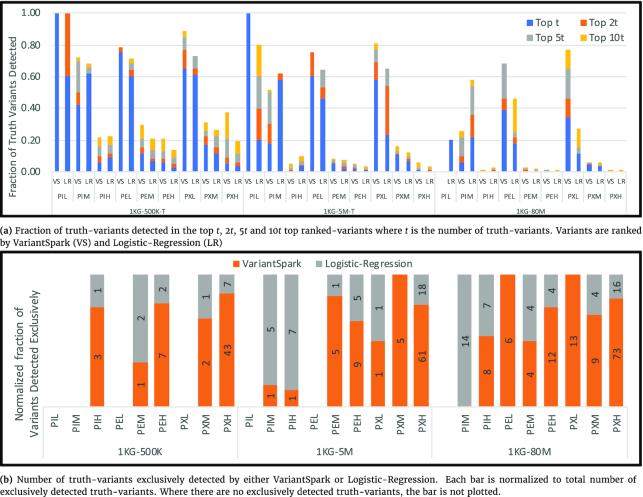
VariantSpark comparison with Logistic-Regression on their ability to detect phenotype-associated variants. Phenotype labels (i.e., PIL, PIM, ...) are described in Table 1.

Fig. 1a shows the fraction of TVs found in the top *r* ranked-variants (RVs) for all phenotype categories (see Table 1) and dataset sizes. More TVs can be detected with higher value of *r*, so we let *r* vary between *t*, 2*t*, 5*t*, and 10*t*, where *t* is the number of TVs (note, *t* is different for each phenotype). We do not consider the order of variants in the list of RVs. All experiments were replicated 3 times. We highlight results from the first replicate for 1KG-80M (2,504 samples and 80M features), 1KG-5M-T (5M variants subset including all truth variants), and 1KG-500K-T here, but other results (second and third replicates, as well as 1KG-5M and 1KG-500K subsets) are reported in Supplementary Data File 1 and support the same conclusion.

Ideally, all TVs are expected to be listed in the top *t* RVs, resulting in a maximum value of 1. VariantSpark indeed achieves this for 2 datasets (1KG-500K-T, 1KG-5M-T) and phenotypes with low numbers of individually associated variables (PIL). LR, on the other hand, only detects all TVs in the smaller of these 2 datasets and only when expanding the list to the top 2*t* RVs.

More generally, VariantSpark detects either more TV or an equivalent proportion for most phenotypes and data set sizes. VariantSpark especially outperforms LR for epistatic (PE) and complex (PX) phenotypes where interactions are involved (achieving scores up to X times better than LR). This is because the association power gained by the interaction between variants remains invisible to LR.

Conversely, LR performs ≤2.2 times better than VariantSpark on datasets withdividual TVs (PIM and PIH). This gain over VariantSpark is likely due to the need to tune hyper-parameter choices for each dataset, which has resulted in non-optimal performance in these instances (see Hyper-Parameter Tuning section).

For phenotypes with a high number of TVs (i.e., PIH, PEH, and PXH) the detection rate is low for both VariantSpark and LR, especially in the case of the largest dataset (1KG-80M). For such complex phenotypes, detecting all TVs, even in the top 10*t* RVs, is a difficult task.

**Figure 2:**
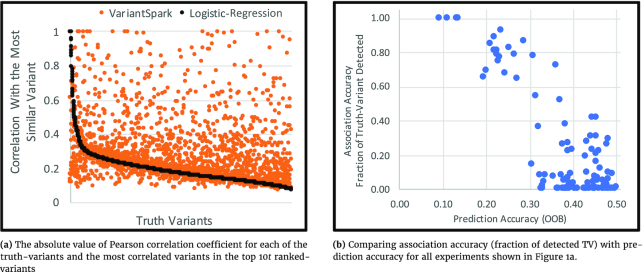
Comparison of exclusively detected variants and correlation with prediction accuracy.

Fig. 2a illustrates a more in-depth comparison of VariantSpark and LR processing the 1KG-80M dataset with PXH phenotype. Note, in this dataset the truth variables are not necessarily present, reflecting a more realistic scenario of associated variants being filtered out by various pre-processing and quality control steps. We hence perform a more qualitative analysis by considering the detection of variants that correlate with TVs (i.e., variants in the same haplotype as a TV). The horizontal axis lists all TVs even if they were not included in the dataset. For each TV we look for the most correlated variant in the top 10*t* RVs and plot the maximum absolute value of the Pearson correlation coefficient (γ). High γ indicates that the detected variant highly correlates with the TV and possibly identifies the same genomic region as the TV. TVs are sorted on the basis of their LR γ. The γ-values for all experiments and both methods are listed in Supplementary Data File 2.

As shown in Fig. 2a while LR quickly exhausts its ability to detect the TV or equivalent variants (γ decreases to <0.5), VariantSpark’s γ stays >0.75 for more variants. We define the number of exclusively detected TVs by VariantSpark as the number of TVs where the VariantSpark γ is >0.75 and LR γ is <0.5. The number of exclusively detected variants by LR is defined similarly. We quantify the number of exclusively detected variants by either method on the 1KG-500K and 1KG-5M datasets. As shown in Fig. 2b the number of exclusively detected TVs by VariantSpark is up to 4.6 times higher than LR (if both VariantSpark and LR detect TVs exclusively). Complete numerical comparisons including for 1KG-5M-T and 1KG-500K-T datasets and the other 2 replicates are provided in Supplementary Data File 1.

It is worth noting that association accuracy, i.e., the ability to recover TV, is distinct from prediction accuracy, i.e., predicting the correct label for a sample. As shown in Fig. 2b, prediction accuracy shows only a moderate correlation with association accuracy (correlation coefficient equal to −0.84). This is because a sufficiently large feature set can create a model that can predict the label by chance, while choosing the TV is a less stochastic process, as demonstrated by the larger value range on the vertical axis. When finding disease genes where the TVs are unknown, using the prediction accuracy to the known labels can only be used as a rough proxy.

### Result 2: VariantSpark outperforms state-of-the-art HPC and distributed implementations

We benchmark VariantSpark against the fastest HPC and distributed implementation of RF, respectively: Ranger and ReForeSt. We record the runtime of all 3 tools on synthetic datasets with 10,000 samples and doubling the number of variants, starting from 100 to 6.5 million and then 10 million.

**Figure 3:**
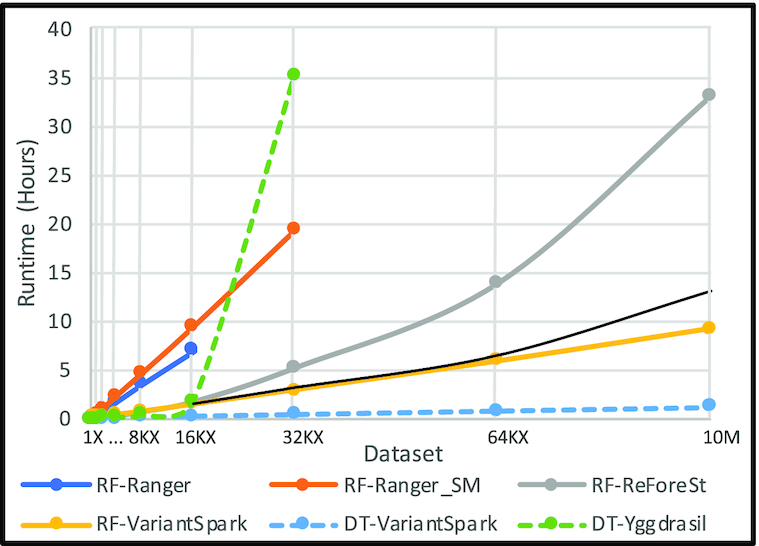
VariantSpark’s runtime compared with other implementations of Random Forest (RF) and Decision Tree (DT). The RF and DT workloads are different and should not be compared with each other. The number of variants in the dataset is doubled at each step (see Table 4 for the list of datasets used for the comparison). The thin unmarked black line illustrates the case if the runtime increases linearly starting from the average runtime of VariantSpark and Reforest for a dataset of 1.6M variants.

Fig. 3 shows that only VariantSpark and ReForeSt were able to process the 2 largest datasets. Ranger fails to process datasets of >1.6M variants, and while in save-memory mode, it processes up to 3.2M, it does so 1.4 times slower than in normal mode. Ranger is executed on a computer with 488 GB of memory. Yet it could not process a dataset of >3.2M owing to excessive memory usage. Note that the biggest dataset in the comparison (10M x 10K) has 100 billion genotypes, which can be loaded into 100 GB of memory, and VariantSpark processes it with a peak memory usage of 120 GB.

For the largest dataset that Ranger processed (1.6M), ReForeSt and VariantSpark performed 4.1 and 4.6 times faster than Ranger, respectively. Ranger advertises a special GWAS mode, but we were unable to successfully run this mode. The quoted runtime in the Ranger publication (for 10,000 samples, 150,000 variants, and mTry = 15,000) shows that GWAS mode is twice as fast as normal mode and uses the same amount of memory as save-memory mode. Given this information, the GWAS mode would not be able to process datasets with >3.2M variants and would remain slower than VariantSpark and ReForeSt.

VariantSpark and ReForeSt performed comparably for 1.6M variants (5,450 and 6,044 seconds, respectively); after this point, the runtime of ReForeSt increases exponentially while VariantSpark increases sub-linearly. Note the thin unmarked black line illustrating a linear runtime increase starting from the average runtime of VariantSpark and Reforest for a dataset of 1.6M variants (5,747 seconds). VariantSpark is 3.6 times faster than ReForeSt processing a dataset with 10M variants, a difference that increases further for larger datasets owing to the exponential vs sub-linear runtime behavior.

We also compare VariantSpark with Yggdrasil, as the only other vertical partitioning implementation. Because Yggdrasil only builds a single DT, we run VariantSpark with mTry equal to the number of variants to emulate building a DT and record the runtime of building DTs 10 times with each method. As shown in Fig. 3, Yggdrasil’s runtime increased dramatically for 3M variants and took 35 hours to complete (possibly due to excessive memory usage). VariantSpark performed 9 and 87.4 times faster than Yggdrasil for a dataset of 1.6M and 3.2M variants, respectively. As mentioned, the biggest dataset requires 100 GB memory to be loaded. While Yggdrasil is executed on a computer cluster with 2 TB of memory, it processes the 3.2M dataset with difficulty.

### Result 3: VariantSpark scales at most linearly with sample and variant increases

**Figure 4:**
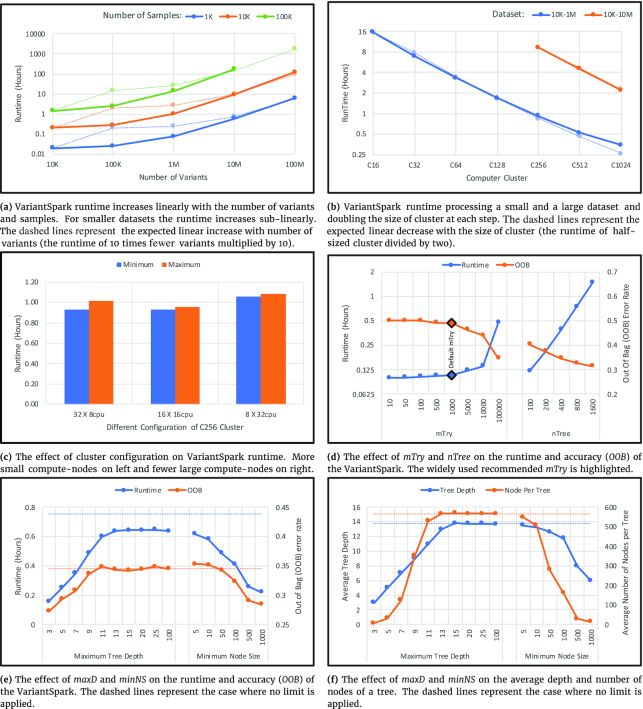
VariantSpark runtime as a function of size of (a) the dataset, (b) the cluster, and (c) the compute-nodes. VariantSpark runtime and accuracy as a function of (d) mTry and nTree and (e) maxD and minNS. (f) The effect of maxD and minNS on the average depth and the number of nodes per tree.

We test VariantSpark’s scalability by recording the runtime when increasing the number of variants 10 times at each step, with 1,000, 10,000, and 100,000 samples, respectively. As shown in Fig. 4a, the runtime increases sub-linearly with the increasing number of variants and increases linearly with the increasing number of samples. Note that both axes are on a logarithmic scale.

VariantSpark can use distributed compute resources efficiently and scales linearly with the size of the cluster as shown in Fig. 4b. It records the speed-up gained when doubling the size of the cluster processing the 10K sample and 1M variant dataset. Up to the C512 cluster, the runtime can be halved (speed-up ∼2). However, using a C1024 cluster, the speed-up decreases to 1.5, which is due to the 10K-1M dataset not being large enough to be efficiently partitioned over 1,024 CPUs and networking becomes a bottleneck. The 2-fold speed-up on this larger cluster is achieved when processing a 10 times larger dataset (10K-10M).

We also investigate whether high-performance compute-nodes perform better than commodity ones, by running the same job on 3 clusters of the overall same capacity but with different numbers and sizes of compute-nodes. Each cluster processes the job 3 times with minimum and maximum runtimes plotted. Fig. 4c shows that compute-node choice has little effect on the runtime. Interestingly, the most expensive HPC computer node (8 computers each with 32 vCPU) delivered a worse runtime compared to a commodity set-up (32 computers each with 8 vCPU), with the best performance delivered by a moderate size computer (16 computers each with 16 vCPU). This is because of the balance between CPUs, memory, and network performance. The runtime variation between replicates is <10%, with the largest difference observed in clusters utilizing more compute-nodes. This is likely due to the increase in networking between nodes, which is subject to external fluctuations.

### Result 4: Hyper-parameter tuning is different for ultra-high-dimensional data

There are 4 important parameters to set when building an RF model:

- nTree: Number of trees in the forest
- mTry: Number of variants evaluated at each node of a tree
- maxD: Maximum depth of a tree to grow
- minNS: Minimum number of samples in a node to be processed

Here, we show that parameter choice substantially affects the performance and accuracy of the trained model. We varied these parameters and recorded runtime and OOB. Also, we record the average number of nodes per tree and average tree depth to show the effect of maxD and minNS on the RF model.

As a rule of thumb, it is recommended to set mTry to the square root of nV; however, our findings show that this recommendation does not suit the analysis of genome-wide datasets. Fig. 4d shows the effect of mTry on the runtime and accuracy of the RF model (10K-1M dataset). mTry = 0.1 × nV = 100,000 shows a substantial improvement in the accuracy compared to the previously recommended mTry = (nV)^1/2^ = 1,000.

Applying limits to RF training to keep trees shallow and efficient affects the runtime and accuracy of the model. Fig. 4e shows the runtime and OOB of VariantSpark when no limits are applied, as well as when maximum depth (maxD) and minimum node size (minNS) are set. Applying these limits reduces the runtime up to 4.8 times. Interestingly, applying these limits also reduces OOB (increases accuracy). This is because deep down in the trees, there are fewer samples in nodes and it is more likely for a variant to gain information by chance. In other words, there is less statistical support for the information obtained from the bottom of a deep tree, and this pushes the model to overfit the data. Also illustrated in this figure is the effect of maxD and minNS on runtime. Note, getting the best accuracy by setting optimal parameter values depends on the complexity of the phenotype and the size of the dataset and hence likely differs between datasets.

Fig. 4d shows that increasing the number of trees nTree increases the runtime linearly as expected. Also, the OOB is reduced (higher prediction accuracy) when doubling the nTree at each step. However, the reduction in OOB is slowed down when training excessive numbers of trees. In other words, we cannot reduce OOB to zero by increasing the number of trees. At this stage, it is not possible to predefine the optimal number of trees because it depends on the complexity of the phenotype and the size of the dataset.

The effect of VariantSpark batch size (number of trees processed in parallel) is recorded in Supplementary Data File 1. An appropriate batch size (depending on the size of cluster and networking performance) can result in the highest speed-up at no cost to the accuracy.

## Methods

VariantSpark is a distributed implementation of the original RF classification algorithm [16]. It accepts ordinal features and a categorical response variable. In the context of GWAS, features are genomic variants and encoded to 0, 1, and 2 for 0/0, 0/1, and 1/1 genotypes, respectively. This is the VariantSpark default encoding when the data are provided in a genomic VCF format. It is possible to use a more complex encoding in a comma-separated value (CSV) format. The user can combine other ordinal omics data with genomics data for multi-omics analysis. In case/control studies, the response variable is a binary phenotype. Yet, VariantSpark can perform multi-class analysis too.

### Importance score captures interactions

When processing a node of a tree, RF evaluates randomly selected variants to separate samples of a node into 2 child nodes (a binary tree). The goal is to keep samples of the same class in 1 of the child nodes. The best split is the variant that results in the highest separation of samples. The best split maximizes the “information gained," which is a metric that measures the quality of separation. VariantSpark uses Gini-Index impurity as described in [16] to compute information gained as used in the original RF.

The samples in each node are selected as a result of the best split in all parent nodes. Thus the best split and information gained in each node depend on all variants selected in the upstream nodes (to the root) and the interaction between them. Given a large number of trees built in an RF model a variant can be selected as the best split in various nodes in the forest. The information gained by the variant in each of these nodes discloses part of its interactions (with variants selected in upstream nodes above it). The importance score of a variant, computed as the average information gained for the variant, represents all of its interactions discovered by the RF model.

### Algorithmic computational complexity

Here we describe the theoretical dependency of VariantSpark’s runtime on different parameters. The runtime of the core computation (excluding loading data to memory) is expected to be linear in nTree × nNode × mTry × nS. The nTree and mTry are directly given by the user and represent the number of trees in the forest and the number of variables to be evaluated for each node of each tree, respectively. nS represents the number of samples in the dataset. To evaluate each variant at each node of a tree, the algorithm needs to loop through all samples. nNode represents the average number of non-leaf nodes per tree.

The value of nNode is determined after the RF is trained because it depends on mTry, maxD, and minNS, as well as the complexity of the phenotype and the size of the dataset. The lower mTry, the lower the chance for a node to divide into pure (leaf) nodes, thus the higher nNode. With a lower maxD or a higher minNS, trees are smaller (nNode is lower). Also, more samples in the dataset result in deeper trees (nodes get purer with more splits), which ultimately increases the nNode. If a phenotype depends only on a few strongly associated signals, trees are shallower and the nNode is smaller.

Given nV = 100M, nS = 10K, nTree = 10K, nNode = 100, and mTry = 0.1 × nV a computer should perform 10^17^ operations to build the RF model. This massive computational requirement indicates the importance of using a distributed computing platform for such analysis.

The number of classes in the phenotype and the number of different values a feature can take also affects the processing time. However, we did not consider their effect because for most analysis the phenotype is a binary value and bi-allelic genotypes are encoded to 0, 1, and 2. The time it takes to load data into memory is a linear function of nS × nV (number of samples and variants in the dataset, respectively).

### Distributed computing

VariantSpark is implemented on top of Apache Spark, a fast distributed computing platform. In the Spark platform, the dataset is partitioned in the memory of several computers (compute-nodes), controlled by a central computer (master-node). In most implementations of machine learning algorithms, the dataset is partitioned by samples (horizontal partitioning) such that each compute-node contains the data for all features and a set of samples. This is because most machine learning datasets include a large number of samples and a small number of features. However, in genomic datasets, it is the number of features that outgrows the number of samples by several orders of magnitude. Partitioning by variants (vertical partitioning) is more effective for genomic data.

**Figure 5:**
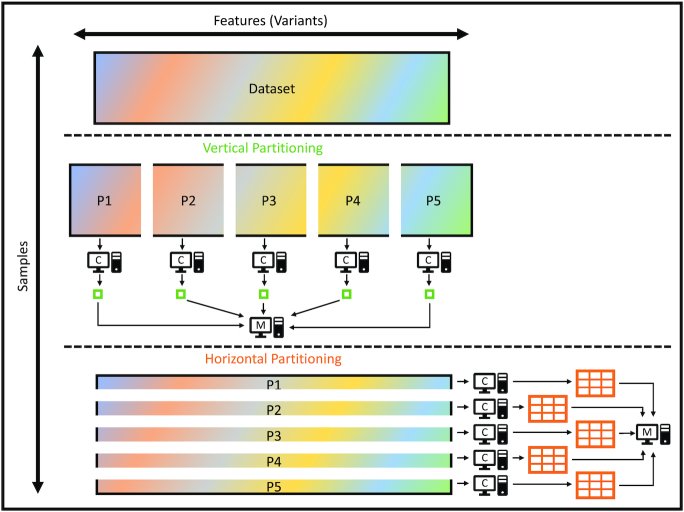
Illustration of partitioning strategies for distributed computing implementations of RF. For genomics data the number of features is larger than the number of samples. Here, vertical partitioning better balances data divisions and makes communication between compute-nodes (C) and the master-node (M) more efficient. Specifically, training each node of each tree with vertical partitioning enables each each compute-node to find the local best split in the allocated partition and to only communicate the best local split with the master (small green squares). In contrast with horizontal partitioning, each compute-node must communicate the summary statistics of all allocated samples with the master node (large orange tables).

Vertical partitioning helps to reduce slow networking operations. If data are partitioned by samples, to process each node of a tree, each compute-node in the cluster must partially evaluate the selected mTry variants and send back results to the master node of the cluster for aggregation. However, when partitioning by variants, each compute-node evaluates a subset of the mTry variants (existing in its local memory) and only sends the information about the best local split to the master-node (Fig. 5).

Another important optimization in VariantSpark is parallelizing the processing of several nodes from several trees in a batch such that network operations never become a bottleneck. Finally, VariantSpark uses Spark RDD, which provides the lowest level of access to the memory to deliver the highest performance.

## Conclusion

While there is evidence for polygenic and epistatic phenotypes, polygenic-epistatic phenotypes have not been studied yet, likely because the existing GWAS methods are underpowered to perform such compute-intensive association studies. VariantSpark is the first methodology to perform complex association analyses on whole-genome sequencing experiments and outperforms other state-of-the-art implementations.

The results provided in this article first demonstrate the capability of VariantSpark in detecting associative signals of complex interactions, and second elaborate the performance and scalability when processing large-scale datasets. Akin to deep learning methods, VariantSpark’s hyper-parameters need to be iteratively tuned to each dataset, which is made possible by VariantSpark’s speed and scalability.

VariantSpark is not a replacement for traditional association analysis but a complement. The results of traditional GWAS (LR) and VariantSpark should be considered together to gain insights into the full influence of the genome on disease and other phenotypes. Similarly, VariantSpark’s output may be usable to prioritize variants in PRS to reduce noise levels.

## Availability of Source Code and Requirements

VariantSpark source code and compilation instructions:

- Project name: VariantSpark
- Project home page: https://github.com/aehrc/VariantSpark
- Operating system(s): Platform independent (Java Virtual Machine)
- Programming language: Scala with Python and Hail Interface
- Other requirements: Java 8, Apache Spark 2
- License: CSIRO Open Source Software Licence v1.0, based on MIT/BSD
- RRID:SCR_018383
- BioTools: biotools:variantspark (https://bio.tools/)

AWS CloudFormation templates to simplify the configuration and installation process:

- Project name: VariantSpark-AWS
- Project home page: https://github.com/aehrc/VariantSpark-aws
- Programming language: YAML
- License: CSIRO Open Source Software Licence v1.0, based on MIT/BSD

## Availability of Supporting Data and Materials

An archival copy of the code is also available in GigaDB [43]. Finally, to facilitate the use of VariantSpark on AWS cloud, we extended our CloudFormation templates and made it available on the AWS Marketplace (https://aws.amazon.com/marketplace/pp/AEHRC-VariantSpark-Notebook/B07YVND4TD) such that a user with minimal technical knowledge can get access to a computer cluster of any size with VariantSpark, Hail, and Jupyter Notebook installed and ready to use.

## Additional Files

**Supplementary Data File 1**. Provides an extended and more detailed numerical comparison for all figures.

**Supplementary Data File 2**. Includes the maximum correlation values (γ) for all experiments.

**Supplementary Data File 3**. Provides all PEPS simulated phenotypes for 1000-Genomes dataset with truth-variants and PEPS configuration files.

Supplementary Data File 4. Explains access to raw data and output file (available on GigaScience Database [43]) as well as technical instruction including:

- 1000-Genome data and subsets in vcf compressed format.
- Dataset simulated with VariantSpark.
- Complete correlation matrix between TVs and RVs for all analyses.
- List of RVs for all analyses.
- Random Forest model created by VariantSpark in JSON format (used to compute average tree depth and number of nodes).
- Instructions to create an AWS EMR cluster via terminal.
- Instructions to submit VariantSpark jobs to the cluster.

## Abbreviations

1KG: 1000-Genomes; AWS: Amazon Web Services; DT: decision tree; GWAS: genome-wide association study; HPC: high-performance computer; LR: logistic regression; OOB: out-of-bag error rate; PEPS: Polygenic Epistatic Phenotype Simulator; PRS: polygenic risk score; RDD: Resilient Distributed Dataset; RF: random forest; RV: ranked variant; TV: truth variant; vCPU: virtual central processing unit.

## Definitions

- nS: Number of samples in dataset
- nV: Number of variants in dataset
- nTree: Number of trees
- mTry: Number of variables to evaluate at each node of a tree
- maxD: Maximum depth of a tree
- minNS: Minimum number of samples in each node to be processed
- rbs: Number of trees to be processed in parallel
- γ: Maximum absolute Pearson correlation coefficient between a truth-variant and any of the ranked-variants

## Funding

Funding was from CSIRO Health and Biosecurity. The authors gratefully acknowledge AWS cloud credits that were used to fund the cloud cost of this work.

## Authors' Contributions

A.B. and D.C.B. designed the experiment. P.S., A.B., B.H., Y.J., C.H., and A.R.O. implemented the software used in this research. A.B. and R.D. conducted the experiments. A.B., D.C.B., O.J.L., and N.T. wrote the manuscript. All authors read and approved the manuscript.

## Supplementary Material

giaa077_GIGA-D-19-00335_Original_Submission

giaa077_GIGA-D-19-00335_Revision_1

giaa077_GIGA-D-19-00335_Revision_2

giaa077_GIGA-D-19-00335_Revision_3

giaa077_GIGA-D-19-00335_Revision_4

giaa077_Response_to_Reviewer_Comments_Original_Submission

giaa077_Response_to_Reviewer_Comments_Revision_1

giaa077_Response_to_Reviewer_Comments_Revision_2

giaa077_Response_to_Reviewer_Comments_Revision_3

giaa077_Reviewer_1_Report_Original_Submission Brendan Lawlor -- 10/13/2019 Reviewed

giaa077_Reviewer_1_Report_Revision_1 Brendan Lawlor -- 5/19/2020 Reviewed

giaa077_Reviewer_2_Report_Original_Submission Joshua W. K. Ho, PhD -- 10/18/2019 Reviewed

giaa077_Reviewer_2_Report_Revision_1 Joshua W. K. Ho, PhD -- 5/12/2020 Reviewed

giaa077_Reviewer_3_Report_Original_Submission Faraz Faghri -- 11/4/2019 Reviewed

giaa077_Supplemental_Files

## References

[1] Visscher PM, Wray NR, Zhang Q, et al. 10 years of GWAS discovery: biology, function, and translation. Am J Hum Genet. 2017;101(1):5–22. 28686856 10.1016/j.ajhg.2017.06.005 PMC5501872

[2] MacArthur J, Bowler E, Cerezo M, et al. The new NHGRI-EBI Catalog of published genome-wide association studies (GWAS Catalog). Nucleic Acids Res. 2017;45(D1):D896–D901. 27899670 10.1093/nar/gkw1133 PMC5210590

[3] Manolio TA, Collins FS, Cox NJ, et al. Finding the missing heritability of complex diseases. Nature. 2009;461(7265):747–53. 19812666 10.1038/nature08494 PMC2831613

[4] Boyle EA, Li YI, Pritchard JK An expanded view of complex traits: from polygenic to omnigenic. Cell. 2017;169(7):1177–86. 28622505 10.1016/j.cell.2017.05.038 PMC5536862

[5] Nicod J, Davies RW, Cai N, et al. Genome-wide association of multiple complex traits in outbred mice by ultra-low-coverage sequencing. Nat Genet. 2016;48(8):912. 27376238 10.1038/ng.3595 PMC4966644

[6] Yang J, Manolio TA, Pasquale LR, et al. Genome partitioning of genetic variation for complex traits using common SNPs. Nat Genet. 2011;43(6):519–25. 21552263 10.1038/ng.823 PMC4295936

[7] Manolio TA, Collins FS, Cox NJ, et al. Finding the missing heritability of complex diseases. Nature. 2009;461(7265):747–53. 19812666 10.1038/nature08494 PMC2831613

[8] Wray NR, Goddard ME, Visscher PM Prediction of individual genetic risk to disease from genome-wide association studies. Genome Res. 2007;17(10):1520–28. 17785532 10.1101/gr.6665407 PMC1987352

[9] Chatterjee N, Shi J, García-Closas M Developing and evaluating polygenic risk prediction models for stratified disease prevention. Nat Rev Genet. 2016;17(7):392. 27140283 10.1038/nrg.2016.27 PMC6021129

[10] Mavaddat N, Pharoah PD, Michailidou K, et al. Prediction of breast cancer risk based on profiling with common genetic variants. J Natl Cancer Inst. 2015;107(5), doi:10.1093/jnci/djv036. PMC4754625 25855707

[11] Phillips PC Epistasis—the essential role of gene interactions in the structure and evolution of genetic systems. Nat Rev Genet. 2008;9(11):855–67. 18852697 10.1038/nrg2452 PMC2689140

[12] Niel C, Sinoquet C, Dina C, et al. A survey about methods dedicated to epistasis detection. Front Genet. 2015;6:285. 26442103 10.3389/fgene.2015.00285 PMC4564769

[13] Shang J, Zhang J, Sun Y, et al. Performance analysis of novel methods for detecting epistasis. BMC Bioinformatics. 2011;12:475. 22172045 10.1186/1471-2105-12-475 PMC3259123

[14] Wan X, Yang C, Yang Q, et al. BOOST: a fast approach to detecting gene-gene interactions in genome-wide case-control studies. Am J Hum Genet. 2010;87(3):325–40. 20817139 10.1016/j.ajhg.2010.07.021 PMC2933337

[15] Evans DM, Spencer CC, Pointon JJ, et al. Interaction between ERAP1 and HLA-B27 in ankylosing spondylitis implicates peptide handling in the mechanism for HLA-B27 in disease susceptibility. Nat Genet. 2011;43(8):761–7. 21743469 10.1038/ng.873 PMC3640413

[16] Breiman L Random Forests. Mach Learn. 2001;45(1):5–32.

[17] Qi Y Random forest for bioinformatics. In: Zhang C, Ma Y Ensemble Machine Learning. Springer; 2012:307–23.

[18] Chen X, Ishwaran H Random forests for genomic data analysis. Genomics. 2012;99(6):323–9. 22546560 10.1016/j.ygeno.2012.04.003 PMC3387489

[19] Goldstein BA, Polley EC, Briggs FB Random forests for genetic association studies. Stat Appl Genet Mol Biol. 2011;10(1):32. 22889876 10.2202/1544-6115.1691 PMC3154091

[20] O’Brien AR, Saunders NFW, Guo Y, et al. VariantSpark: population scale clustering of genotype information. BMC Genomics. 2015;16:1052. 26651996 10.1186/s12864-015-2269-7 PMC4676146

[21] Eraslan G, Avsec Ž, Gagneur J, et al. Deep learning: new computational modelling techniques for genomics. Nat Rev Genet. 2019;20(7):389–403. 30971806 10.1038/s41576-019-0122-6

[22] Zaharia M, Xin RS, Wendell P, et al. Apache Spark: a unified engine for big data processing. Commun ACM. 2016;59(11):56–65.

[23] Massie M, Nothaft F, Hartl C, et al. ADAM: genomics formats and processing patterns for cloud scale computing. Technical Report No. UCB/EECS-2013-207 EECS Department, University of California Berkeley; 2013.

[24] Stephens ZD, Lee SY, Faghri F, et al. Big data: astronomical or genomical?. PLoS Biol. 2015;13(7):e1002195. 26151137 10.1371/journal.pbio.1002195 PMC4494865

[25] Hail library. https://github.com/hail-is/hail. Accessed on July 1, 2019.

[26] Wright MN, Ziegler A Ranger: a fast implementation of random forests for high dimensional data in C++ and R. J Stat Softw. 2016;77, doi:10.18637/jss.v077.i01.

[27] Liaw A, randomForest package for R, https://cran.r-project.org/web/packages/randomForest/randomForest.pdf. Accessed on July1, 2019.

[28] Ishwaran H, Kogalur UB, Blackstone EH, et al. Random survival forests. Ann Appl Stat. 2008;2(3):841–60.

[29] Schwarz DF, König IR, Ziegler A On safari to Random Jungle: a fast implementation of random forests for high-dimensional data. Bioinformatics. 2010;26(14):1752–8. 20505004 10.1093/bioinformatics/btq257 PMC2894507

[30] Telenti A, Pierce LCT Deep sequencing of 10,000 human genomes. Proc Natl Acad Sci U S A. 2016;113(42):11901–6. 27702888 10.1073/pnas.1613365113 PMC5081584

[31] Bayardo BP, Herbach JS, Basu S, et al. PLANET: massively parallel learning of tree ensembles with MapReduce. In: Proceedings of the 35th International Conference on Very Large Data Bases. 2009, doi:10.14778/1687553.1687569.

[32] Meng X, Bradley J, Yavuz B, et al. MLlib: Machine learning in Apache Spark. J Mach Learn Res. 2016;17(1):1235–41.

[33] Bayat A, Szul P, O’Brien AR, et al. VariantSpark, a random forest machine learning implementation for ultra high dimensional data. bioRxiv. 2019, doi:10.1101/702902.

[34] Lulli A, Oneto L, Anguita D ReForeSt: random forests in Apache Spark. In: Lintas A, Rovetta S, Verschure P et al. et al., eds. International Conference on Artificial Neural Networks. Cham: Springer; 2017:331–9.

[35] Pafka S benchm-ml. https://github.com/szilard/benchm-ml. Accessed on July 1, 2019.

[36] Chen T, Guestrin C Xgboost: a scalable tree boosting system. In: Proceedings of the 22nd ACM SIGKDD International Conference on Knowledge Discovery and Data Mining. ACM; 2016:785–94.

[37] H2O: Open-source machine learning platform for enterprises. https://www.h2o.ai/h2o/. Accessed on July 1, 2019.

[38] Oneto L, Cipollini F, Lulli A, et al.ReForeSt. https://github.com/alessandrolulli/reforest. Accessed on July 1, 2019.

[39] Chen J, Li K, Tang Z, et al. A parallel random forest algorithm for big data in a spark cloud computing environment. IEEE Trans Parallel Distrib Syst. 2016;28(4):919–33.

[40] Abuzaid F, Bradley JK, Liang FT, et al. Yggdrasil: an optimized system for training deep decision trees at scale. Adv Neural Inf Process Syst. 2016;29:3817–25.

[41] Breiman L, Friedman J, Olshen RA, et al. Classification and Regression Trees. 1st ed. Belmont, CA, USA: Wadsworth; 1984.

[42] Kleinbaum DG, Dietz K, Gail M, et al. Logistic Regression. Springer; 2002.

[43] Bayat A, Szul P, O’Brien AR, et al. Supporting data for “VariantSpark: cloud-based machine learning for association study of complex phenotype and large-scale genomic data.”. GigaScience Database. 2020; 10.5524/100759. PMC7407261 32761098

[44] 1000 Genomes Project Consortium A global reference for human genetic variation. Nature. 2015;526(7571):68. 26432245 10.1038/nature15393 PMC4750478

[45] Bayat A, Hosking B, PEPS: Polygenic Epistatic Phenotype Simulator. https://github.com/aehrc/PEPS. Accessed on July 1, 2019.

