# Supplementary material for: VariantSpark: Cloud-based machine learning for association study of complex phenotype and large-scale genomic data
Source: Gigascience. 2020 Aug 6;9(8):giaa077. doi: 10.1093/gigascience/giaa077 (PMC7407261; doi:10.1093/gigascience/giaa077)
Supplement: giaa077_GIGA-D-19-00335_Original_Submission [file giaa077_giga-d-19-00335_original_submission.pdf]

GigaScience

VariantSpark: A Distributed Implementation of Random Forest Tailored for Ultra High Dimensional Genomic Data

--Manuscript Draft--

**Manuscript Number:**	GIGA-D-19-00335
**Full Title:**	VariantSpark: A Distributed Implementation of Random Forest Tailored for Ultra High Dimensional Genomic Data
**Article Type:**	Research
**Funding Information:**	
**Abstract:**	The demands on machine learning methods to cater for ultra high dimensional datasets with millions of features have been increasing in domains like life sciences and the Internet of Things (IoT). While Random Forests are suitable for ``wide'' datasets, current implementations such as Google's PLANET lack the ability to scale to such dimensions. Recent improvements by Yggdrasil begin to address these limitations but do not extend to Random Forest. This paper introduces VariantSpark, a novel Random Forest implementation on top of Apache Spark. VariantSpark uses vertical partitioning and trains multiple trees in parallel. VariantSpark is up to 9 and 89 times faster than Google's PLANET and Yggdrasil, respectively, and is the first method capable of scaling to millions of features.
**Corresponding Author:**	Arash Bayat<br><br>AUSTRALIA
**Corresponding Author Secondary Information:**	
**Corresponding Author's Institution:**	
**Corresponding Author's Secondary Institution:**	
**First Author:**	Arash Bayat
**First Author Secondary Information:**	
**Order of Authors:**	Arash Bayat
	Piotr Szul
	Aidan R. O'Brien
	Robert Dunne
	Oscar J. Luo
	Yatish Jain
	Brenden Hosking
	Denis Bauer
**Order of Authors Secondary Information:**	
**Additional Information:**	
**Question**	**Response**
Are you submitting this manuscript to a special series or article collection?	No
**Experimental design and statistics**<br><br>Full details of the experimental design and	Yes

Powered by Editorial Manager® and ProduXion Manager® from Aries Systems Corporation

statistical methods used should be given in the Methods section, as detailed in our Minimum Standards Reporting Checklist. Information essential to interpreting the data presented should be made available in the figure legends.<br><br>Have you included all the information requested in your manuscript?	
**Resources**<br><br>A description of all resources used, including antibodies, cell lines, animals and software tools, with enough information to allow them to be uniquely identified, should be included in the Methods section. Authors are strongly encouraged to cite Research Resource Identifiers (RRIDs) for antibodies, model organisms and tools, where possible.<br><br>Have you included the information requested as detailed in our Minimum Standards Reporting Checklist?	Yes
**Availability of data and materials**<br><br>All datasets and code on which the conclusions of the paper rely must be either included in your submission or deposited in publicly available repositories (where available and ethically appropriate), referencing such data using a unique identifier in the references and in the "Availability of Data and Materials" section of your manuscript.<br><br>Have you have met the above requirement as detailed in our Minimum Standards Reporting Checklist?	Yes

Powered by Editorial Manager® and ProduXion Manager® from Aries Systems Corporation

Click here to download Manuscript main.tex

GigaScience, 2017, 1–6

doi: xx.xxxx/xxxx
Manuscript in Preparation
Paper

PAPER

VariantSpark: A Distributed Implementation of Random Forest Tailored for Ultra High Dimensional Genomic Data

Arash Bayat[1,*], Piotr Szul[2,†], Aidan R. O'Brien[2,*], Robert Dunne[2,†], Oscar J. Luo[2,*], Yatish Jain[2,*], Brendan Hosking[2,*] and Denis C. Bauer[2,*]

[1]Health and Biosecurity, CSIRO, Australia and [2]Data61, CSIRO, Australia

[*]Arash.Bayat@csiro.au; Denis.Bauer@csiro.au
[†]Contributed equally.

Abstract

The demands on machine learning methods to cater for ultra high dimensional datasets with millions of features have been increasing in domains like life sciences and the Internet of Things (IoT). While Random Forests are suitable for "wide" datasets, current implementations such as Google's PLANET lack the ability to scale to such dimensions. Recent improvements by Yggdrasil begin to address these limitations but do not extend to Random Forest. This paper introduces VariantSpark, a novel Random Forest implementation on top of Apache Spark. VariantSpark uses vertical partitioning and trains multiple trees in parallel. VariantSpark is up to 9 and 89 times faster than Google's PLANET and Yggdrasil, respectively, and is the first method capable of scaling to millions of features.

Key words: Bioinformatics; GWAS; Machine Learning; Cloud Computing

Introduction

The ongoing digital revolution is causing a dramatic increase in data collected about almost every aspect of life [1]. These datasets are not only growing by capturing more events (samples) but also by capturing more information about these events (features). The challenge of "big" and "wide" data is especially pronounced in the biomedical space where, for example, whole-genome sequencing technology enables researchers to extract over 3 billion features from the human genome for analysis [2]. Other domains are also seeing a rapid increase in the number of features processed by statistical or machine learning applications [3].

While statistical linear models can deal with such wide datasets by analyzing each feature independently [4], there is a growing demand for more realistic approaches that can discover interacting features using machine learning [5]. In the life-science space, this would allow modelling the interactions between genes that result in complex traits like height [6] or diseases like obesity [7]. In particular, Decision Tree [5] based models have been successfully applied to uncover interactions between features [8]. Other examples of using Random Forest in GWAS context includes [9, 10, 11, 12].

As Decision Trees fitting algorithms are greedy and may yield an estimate with high variance, Breiman developed an ensemble approach for improving accuracy by aggregating over a large number of Decision Trees, called Random Forest [13]. Random Forest models are particularly well suited for datasets that are wide and there is a need to capture interactions for two reasons:

- Wide datasets, particularly when there are more features than samples, cause other machine learning methods to overfit easily, whereas Random Forest models are more resistant to this "curse of dimensionality" [14, 15]
- There is significant scope for parallelization in Random Forest algorithms allowing forests to be grown efficiently even on large datasets.

Compiled on: September 26, 2019.
Draft manuscript prepared by the author.

Key Points

- Random Forest has been used in genome-wide association studies but has never been scaled to the whole genome data.
- VariantSpark is a distributed implementation of Random Forest tailored for high dimensional genomic data.
- Vertical data partitioning and parallel tree growing allow VariantSpark to process a dataset of millions of variants and samples.

Table 1. Methods evaluated

Evaluated Methods	Implemented Language	Computational Platform	Reference
VariantSpark	Scala	Spark Cluster	Our work
Google's PLANET (Spark MLlib)	Scala Cluster	Spark	[18]
Yggdrasil	Scala	Spark Cluster	[3]
H2O.ai	Scala & R	Proprietary Cluster	[17]
Random Forest	*Fortran* & R	HPC	[13]
Ranger	C++ & R	HPC	[16]

The first implementations of Random Forest in R (Random Forest) were based on the original *Fortran* code by Breiman [13]. Later Ranger [16] provided a C++ implementation of Random Forest with an R interface, which also covers the loading and pre-processing. H2O.ai [17] provides another R interfacing implementation with Scala back end. In terms of parallelization, all of these implementations are optimized for a high-performance computing (HPC) machine (a single computing node). However, the Random Forest algorithm allows for parallelization on a distributed computing platform.

Apache Spark is particularly suitable for such massively parallel interconnected calculations as it offers a distributed computing architecture that enables communication beyond compute-node boundaries in a standardized approach [2]. In the Spark cluster, there are several workers and a master each of which is a computing node in the network. The Driver program (run on the master node) coordinates the job flow by controlling the executors (run on the worker nodes).

Google's PLANET [18] is a *MapReduce* implementation of Random Forest and the first to parallelize processing each node of a tree in a distributed fashion. Hence, the ideas from Google's PLANET are now used in many "Big Data" machine learning libraries, such as Spark MLlib and *XGBoost* [19]. Google's PLANET partitions data by samples with workers holding all features for a fraction of samples. However, this solution produces an approximate split and limits the depth of tree for high dimensional datasets [3].

Yggdrasil [3] overcomes this limitation by flipping the dataset and partitioning it by features rather than samples. The Driver aggregates the best local splits computed on the executors, identifies the best global split, and updates the executors accordingly. However, the work is limited to Decision Trees and does not implement bootstrapping or *mtry* (number of features considered at each node), which are essential components in a Random Forest.

VariantSpark extends Yggdrasil's approach to Decision Trees to Random Forest models. VariantSpark also introduces a novel method of parallelization in the tree growing process such that nodes of different trees are processed in parallel. This enables highly accurate multivariate models to be built on large datasets with millions of features. For more details see *Implementation*.

In the result section, we evaluated different implementations of Random Forest, including Google's PLANET, for their ability to scale to large datasets and then tests the limits of VariantSpark. Finally, we benchmark VariantSpark against Yggdrasil. Implementation details are elaborated in the method section.

Results and discussion

VariantSpark outperforms existing methods for multi-class classification

Figure 1a shows the execution time of all methods on different sized datasets. Note that not all programs were able to process all datasets due to out of memory errors and time-out. VariantSpark is faster than all other methods for all dataset except for the smallest dataset (500K) where Ranger R implementation is faster (70 seconds *vs.* 110 seconds). The Ranger R is the second-fastest implementation of Random Forest but cannot process the largest dataset (80M features). VariantSpark is 4.5, 5.3 and 4.2 times faster than Ranger R processing 1M, 6M, and 20M dataset respectively. Google's PLANET is the only other Spark implementation of Random Forest but cannot process dataset larger than 6M features. VariantSpark is 9.3 and 26.4 times faster than Google's PLANET processing 1M and 6M dataset respectively.

VariantSpark is the only method able to scale to the largest dataset (80M features). This demonstrates that although Google's PLANET was designed to handle many samples, it is unable to efficiently cope with a large number of features. Extrapolating from this, the dotted line in Figure 1a shows that all current implementations would require between 27 hours and 11 days to finish analyzing the largest dataset (80M), while VariantSpark completes the task in about 3 hours on a small cluster set-up (C1).

Figure 1b compares the accuracy and speed of the different Random Forest implementations on the largest dataset they successfully complete. The accuracy is measured in terms of out-of-bag error rate (lower is better) and the speed is computed as million variants per second (higher is better). VariantSpark is the fastest implementation and delivers the most accurate result as it was the only method able to utilize the whole genome (80M).

The runtime of VariantSpark can be substantially improved due to its ability to efficiently utilize large numbers of commodity computers. Table 2 shows the runtime when utilizing a large Spark cluster (C2) to run the same analysis as above. We can observe that the speed-up improvement grows with data set size, with up to 5-fold speed-up for 80M (from $11,760$ to $2,214$ seconds). Also, noteworthy is the reduction in error when the whole data set is utilized.

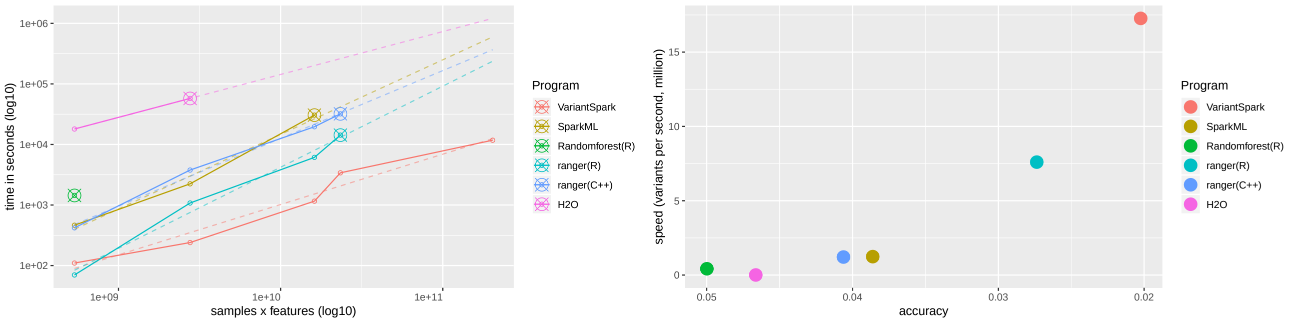

(a) Time consumption

(b) Accuracy vs speed

Figure 1. Comparison of speed and accuracy of VariantSpark with other published methods. SparkML refers to Google's PLANET. Crossed cicles (⊗) mark the last successfully process dataset by the respective methods.

Table 2. Accuracy and scalability of a multi-class classification with 50 trees using VariantSpark

Dataset	Error Rate (OOB)	Runtime (min) C2	C1	Speed up
1M	0.06	1.6	4.0	2.5
6M	0.04	4.2	19.3	4.6
20M	0.02	10.8	56.7	5.2
80M	0.01	36.9	196.0	5.3

VariantSpark is linearly scalable with samples, features and CPUs

Here, we explore the performance of VariantSpark in more detail by testing its ability to scale beyond the real dataset size. We hence generate synthetic datasets with up to 50 million features (i.e., p = 50,000,000), with up to 10,000 samples.

We measured the runtime to build a binary Random Forest model of 100 trees with a fixed *mtry* fraction of 0.25 using our C2 computer with these different synthetic datasets. VariantSpark scales linearly with an increase in feature sizes p and sublinearly with the increase in sample size n (see Figure 2a). VariantSpark also scales well when increasing the number of CPUs making cloud-application with on-demand cluster sizes possible (see Figure 2b). As shown in Table 2 the 80M dataset can be processed in about 37 minutes. With an increase in the number of executors, the execution time can further decrease.

Wide data and the choice of mtry and ntree

VariantSpark is a partial implementation of the original algorithm of [13]. As such the choice of ntree and mtry follows the same logic. However, the original choices of the default values of these parameters were based on Breiman's experience with a number of data sets [13]. VariantSpark may be operating in regions where a different set of heuristics may be needed to guide the parameter settings.

In this section, we test the limits of VariantSpark in association and classification analysis on high-dimensional data. For this purpose, we generated a synthetic dataset with 2.5 million features (i.e., p = 2,500,000), of which 5 are designed to be related to the response variable, with 5,000 samples (i.e. n = 5,000). We use our C2 computer for this analysis. More details are available in the method section.

We fit the Random Forest model and estimate the classification accuracy by capturing the OOB error. We also measure the feature selection performance by capturing the rank-biased overlap (RBO) measure [20]. RBO assesses whether VariantSpark is able to retrieve the 5 features in order of their association weight as a scale from 0 (no feature recovered) to 1 (fully recovered).

See Figure 3a for plots of these two measures for this example. Note that, for the parameter mtry, the plot shows the proportion, mtry/p, which means that the default value of 1581 is shown as 1581/$p \approx 0.0006$. The default value for mtry does not result in good classification performance for this large feature dataset. The OOB for this value of mtry does not drop below 0.5, even when the number of trees is increased. Increasing mtry in combination with ntree yields the best performance with the OOB error essentially constant around 0.4 across a large range of mtry and ntree values. This is in contrast to the feature-selection performance, where the RBO measure heavily depends on ntree and gives better results with lower values of mtry (Figure 3b).

This may be because a large mtry leads to more correlated trees as the same important features have a higher chance of being selected in all trees, which does not yield good performance outcomes. This issue is less pronounced for classification error where random features can mimic the response variable, hence resulting in more in a performance plateau. Increasing the number of trees, on the other hand, improves performance especially when the trees are kept diverse (small mtry) but appropriate for large feature datasets (mtry larger than default). The ability of VariantSpark to build decision trees in parallel at the compute node level hence caters perfectly to the requirement of large feature datasets as more trees can be built given a fixed time budget.

VariantSpark tree-building method is faster than Yggdrasil

Here we compare the tree building implementation of VariantSpark to Yggdrasil. Both methods partition the data by features to parallelize Gini computation, which was demonstrated to yield better performance for wide data than Google's PLANET, which partitions by samples [3]. Since VariantSpark is an ensemble method, we approximate the single Decision Tree functionality of Yggdrasil by (1) disabling bootstrapping, (2) setting $mtry = p$, (3) setting $nTree = 1$.

The largest dataset we were able to process with Yggdrasil is dataset 1M. It took 102 seconds (average over two runs) for Yggdrasil to build a Decision Tree for this dataset, compared to 3 seconds for VariantSpark (average over two trees, $mtry = p$ and without bootstrapping). This shows nearly 33 times speedup

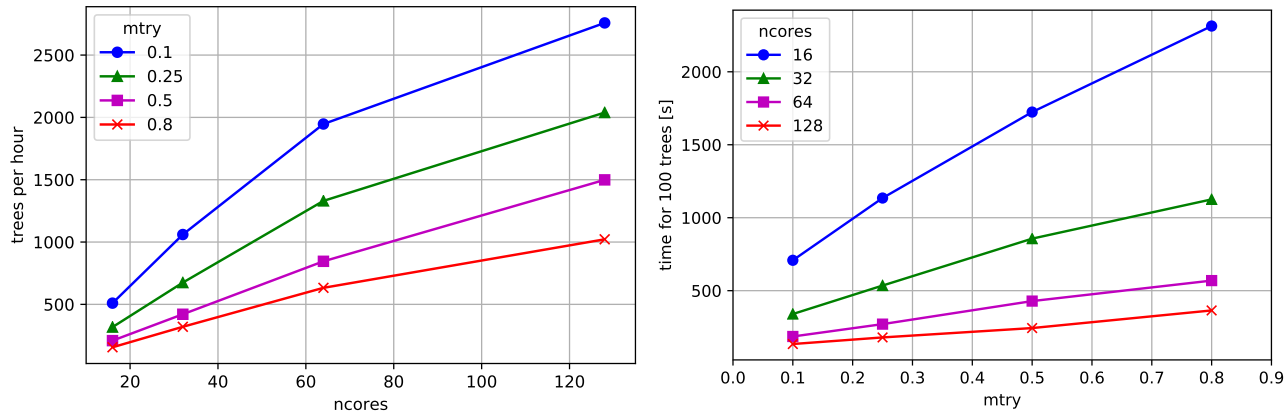

(a) Runtime as a function of increasing number of samples and/or features

(b) Scalability with increasing number of cores

Figure 2. Performance in response to varying ntree and mtry

Table 3. Datasets used for evaluations

Dataset	Description	Samples	Features
80M	1000 Genomes Phase 3 chr1 to chr22	2,540	81,047,467
20M	1000 Genomes Phase 3 chr1 to chr3	2,504	19,328,051
6M	1000 Genomes Phase 3 chr1	2,504	6,450,364
1M	1000 Genomes Phase 3 chr22	2,504	1,103,548
500K	1000 Genomes Phase 1 chr 22	1,092	490,036

over Yggdrasil. Enabling parallel tree-building reduces the runtime for VariantSpark to an average of 1.14 second to build each tree, which represents an 89-fold speed-up over the Yggdrasil implementation. The time mentioned above only includes the actual training process, not data loading and other auxiliary steps. For this experiment, we use C3 cluster as described in Table 4.

Methods

Datasets

For the evaluation, well-known genomic datasets (1000 Genomes Project [21]) along with a synthetic dataset are used. The genomic dataset is subsetted to create 5 datasets of different size (see Table 3). Each dataset includes the genomic profile as well as the ethnicity of a few thousand individual humans. In our evaluation, the person's ethnicity (response variable) is predicted from his or her genomic profile and evaluated against the known ethnicity. The genomic profile is a set of features taking the values 0/0, 0/1 or 1/1 which we encode as 0, 1 and 2 respectively.

For the synthetic dataset, we use the method provided by Wright and Ziegler [16]. The synthetic dataset consists of n samples and p features where $p >> n$ and values for each feature are ordinal with three levels represented as numbers 0, 1 and 2 (which correspond to an additive effect encoding of genomic variation) randomly generated from a uniform distribution with equal probabilities. For all synthetic dataset, the response variable is a function of five randomly selected features.

The model parameters we use for simulations are $w_i = 1/\sqrt{2^i - 1}$ for $i = 1, \cdots, 5$ and we set $z = \sum_{i=1}^{5} w_i x_i$. We let $\sigma_\epsilon^2 = \mathrm{Var}(z)(1-\theta)/\theta$ where θ is a parameter controlling the fraction of variance explained by the informative features, and in our study we chose $\theta = 0.125$. Then $y = z + \epsilon$ where $\epsilon \sim N(0, \sigma_\epsilon^2)$. The dichotomous response is generated by thresholding y at the 0.5 quantile:

$$\ddot{y} = \begin{cases} 0 & \text{for } y \geq Q2(y) \\ 1 & \text{for } y > Q2(y). \end{cases} \quad (1)$$

Computational resources

Computational platforms described in Table 4 are used in our experiments.

Implementation

VariantSpark implements the original algorithm of Breiman [13]. Given the dataset of n samples and p features, in our implementation, the dataset is partitioned by features and each partition is allocation in a worker such that the ith worker holds p_i features for all samples where $\sum_i p_i = p$. To build each tree, the Driver first bootstrap samples thus different bootstrapped set of samples are used to build each tree. Then, for each node of each tree *mtry* different features are randomly picked from the dataset to compute Gini Impurity (parallelized over all executors). Since features are partitioned across workers, Each executor (assume one per worker) randomly pick $mtry \times p_i$ features and finds the best split locally for the node. The Driver aggregates all local best split for each node finds the global best split and updates all executors about the global best split. This process is carried out across *rbs* trees in parallel where *rbs* is set by the user. Hence multiple nodes of multiple trees are processed in parallel.

We keep track of the change in Gini Impurity scores after splitting at each node in each tree. This information is used to calculate the Importance Score which is used as a metric to quantify the contribution of each feature in classifying the samples.

This implementation avoids communication bottlenecks be-

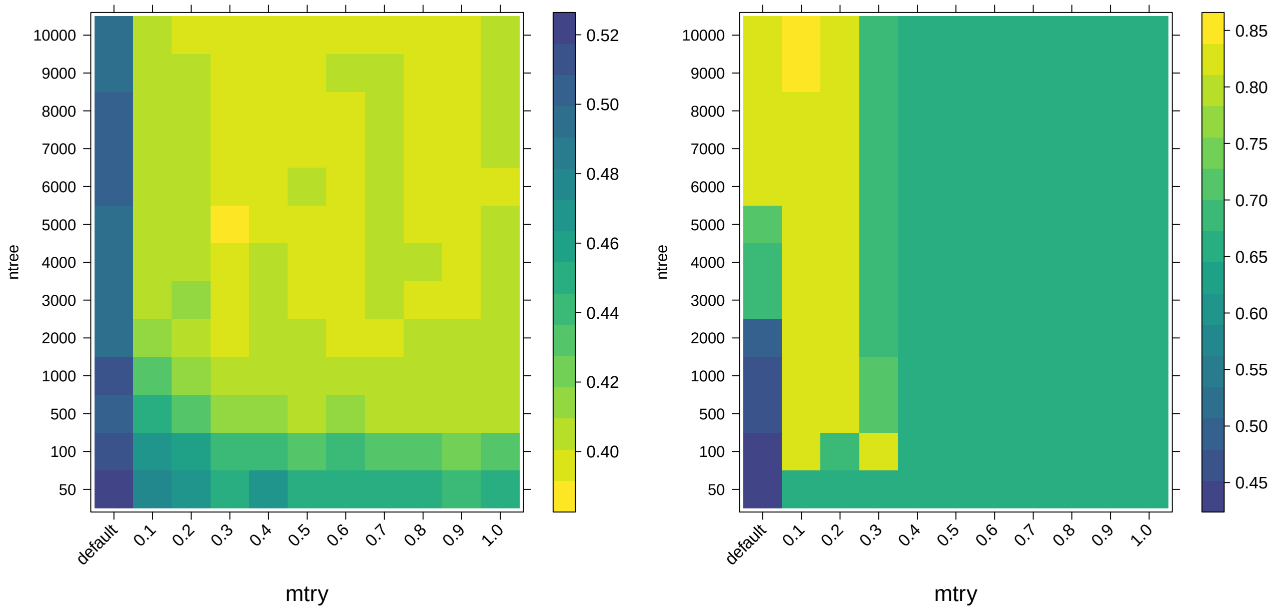

(a) OOB classification error

(b) RBO feature selection error

Figure 3. Accuracy performance in response to varying ntree and mtry. mtry is given as a fraction of p.

Table 4. Computational resources used in our experiment

	Description	Number of executors	Total Memory (GB)
C1	A Spark 1.6.1 cluster with 12 worker nodes each with 16 Intel Xeon E5-2660@2.20GHz CPU cores and 128 GB of memory	32	32×8
C2	A Spark 1.6.1 cluster with 12 worker nodes each with 16 Intel Xeon E5-2660@2.20GHz CPU cores and 128 GB of memory	130	130×8
C3	An Amazon aws emr cluster with r4.2xlarge machine as master and8 r4.4xlarge machines as worker nodes	8×16	8×61

tween the Driver and the executors as information exchange is minimal allowing it to build large numbers of trees efficiently. Furthermore, VariantSpark has memory-efficient representation of genomics data, optimized communication patterns, and computation batching. It also provides an efficient implementation of Out-Of-Bag (OOB) error calculation, which substantially simplifies parameter tuning over the more computationally expensive alternative of cross-validation.

VariantSpark is available as a *Github* repository
The GitHub repository (https://github.com/aehrc/VariantSpark) holds information for setting up on any local or cloud-based computing environment supporting Apache Spark, such as *Amazon Web Services* and *Google Cloud Platform*. In addition, VariantSpark can also be accessed through a notebook interface hosted at *Databricks*
(https://aehrc.github.io/VariantSpark/notebook-examples/VariantSpark_HipsterIndex.html). Note that, in addition to *VCF* and *CSV* file format, VariantSpark also works with the *ADAM16* data schema, implemented on top of *Avro* and *Parquet*, as well as the *HAIL API* (https://github.com/hail-is/hail) for variant pre-processing.

Usage of the Gini Impurity score as a data splitting criterion

The current implementation of VariantSpark uses a Gini Impurity criterion to choose the feature for splitting at each node. This was introduced for Decision Tree in Breiman *et al.* [5]. For each node A in each tree, except leaf nodes, the program finds the feature (between randomly selected *mtry* features) that best splits samples in A into two child node L and R. Assume node A includes m samples each of which labelled with q, where $q \in 1, \ldots, Q$. Given f_q as the fraction of samples in the R which are labeled as q, then the Gini Impurity of the node A ($Gini_R$)is computed as,

$$Gini_R = 1 - \sum_{q=1}^{Q} {f_q}^2. \tag{2}$$

Assume l and r are the fractions of samples in A which are moved to L and R respectively ($l + r = 1$) after splitting node A with feature t. The Information Gained by feature t (IG_t) at node A is computed as Gini Impurity of A minus weighted average of Gini Impurity of L and R using equation,

$$IG_t = Gini_A - (x \times Gini_L + y \times GiniR) \tag{3}$$

The feature that results in the highest Information Gained is selected as the local best split for the node by executor. When the Driver collects all local best splits it looks for the one that maximizes Information Gained and chooses it as the best global split.

Feature t might be selected as the best split for multiple nodes of multiple trees. The raw Importance Score of feature t is defined as the mean of IG_t across all node in the Random Forest where t is chosen as best split.

Out-of-Bag training and testing procedure

The Random Forest algorithm builds each tree on a subset of individuals (approximately two thirds), leaving the other third out. The algorithm then tests each tree against the held-out samples, giving an estimate of the error for each tree. Averaging the error for each tree returns the OOB error for the model. According to Breiman [5], the out-of-bag estimate is as accurate as using a test set of the same size as the training set.

Conclusion

The challenge of “big” and “wide” data is especially pronounced in the biomedical space where dataset acquisition is predicted to far outpacing that of traditional “Big Data” disciplines [22]. Catering for this, we extended Random Forest to cope with extremely high dimensional data using a novel parallelization approach enabled by Spark. Compared to Google’s PLANET and other non-Spark implementations, as well as the purpose-designed Yggdrasil, VariantSpark can scale to millions of features. It also offers the fastest training method for Random Forest on a wide range of data-sets sizes compared to the other tools tested.

References

1. Loebbecke C, Picot A. Reflections on societal and business model transformation arising from digitization and big data analytics: A research agenda. The Journal of Strategic Information Systems 2015;24(3):149–157. 10.1016/j.jsis.2015.08.002.
2. O’Brien AR, Saunders NFW, Guo Y, Buske FA, Scott RJ, Bauer DC. VariantSpark: population scale clustering of genotype information. BMC Genomics 2015;16(1). 10.1186/s12864-015-2269-7.
3. Abuzaid F, Bradley JK, Liang FT, Feng A, Yang L, Zaharia M, et al. Yggdrasil: An Optimized System for Training Deep Decision Trees at Scale. Advances in Neural Information Processing Systems 2016;29:3817–3825.
4. Consortium WTCC. Genome-wide association study of 14,000 cases of seven common diseases and 3,000 shared controls. Nature 2007;447(7145):661–678. 10.1038/nature05911.
5. Leo Breiman CJSRAO Jerome Friedman. Classification and Regression Trees. 1 ed. Belmont, California, U.S.A.: Wadsworth Publishing Company; 1984.
6. Lello L, Avery SG, Tellier L, Vazquez A, Campos Gdl, Hsu SD. Accurate Genomic Prediction Of Human Height. arXiv preprint arXiv:170906489 2017;.
7. Locke AE, Kahali B, Berndt SI, Justice AE, Pers TH, et al. Genetic studies of body mass index yield new insights for obesity biology. Nature 2015;518(7538):197–206. https://www.ncbi.nlm.nih.gov/pubmed/25673413.
8. Wright MN, Ziegler A, König IR. Do little interactions get lost in dark random forests? BMC Bioinformatics 2016;17(1):145. 10.1186/s12859-016-0995-8.
9. Qi Y. Random forest for bioinformatics. In: Ensemble machine learning Springer; 2012.p. 307–323.
10. Li J, Malley JD, Andrew AS, Karagas MR, Moore JH. Detecting gene-gene interactions using a permutation-based random forest method. BioData mining 2016;9(1):14.
11. Botta V, Louppe G, Geurts P, Wehenkel L. Exploiting SNP correlations within random forest for genome-wide association studies. PloS one 2014;9(4):e93379.
12. Meng YA, Yu Y, Cupples LA, Farrer LA, Lunetta KL. Performance of random forest when SNPs are in linkage disequilibrium. BMC bioinformatics 2009;10(1):78.
13. Breiman L. Random Forests. Machine Learning 2001;45(1):5–32.
14. Bauer DC, Gaff C, Dinger ME, Caramins M, Buske FA, Fenech M, et al. Genomics and personalised whole-of-life healthcare. Trends in Molecular Medicine 2014;20(9):479–486. 10.1016/j.molmed.2014.04.001.
15. Bellman R, Bellman RE. Adaptive Control Processes: A Guided Tour. Princeton University Press; 1961.
16. Wright MN, Ziegler A. Ranger: A Fast Implementation of Random Forests for High Dimensional Data in C++ and R. Journal of Statistical Software 2016;.
17. H2O, H2O, editor, Open-source machine learning platform for enterprises, https://www.h2o.ai/h2o/. web; 2018. https://www.h2o.ai/h2o/.
18. Bayardo BP, Herbach JS, Basu S, J R. PLANET: Massively Parallel Learning of Tree Ensembles with MapReduce. Proceedings of the 35th International Conference on Very Large Data Bases (VLDB-2009) 2009;.
19. Chen T, Guestrin C. Xgboost: A scalable tree boosting system. In: Proceedings of the 22nd acm sigkdd international conference on knowledge discovery and data mining ACM; 2016. p. 785–794.
20. Webber W, Moffat A, Zobel J. A Similarity Measure for Indefinite Rankings. ACM Transactions on Information Systems 2010;28(4):20:1–20:38. 10.1145/1852102.1852106.
21. Consortium GP, et al. A global reference for human genetic variation. Nature 2015;526(7571):68.
22. Stephens ZD, Lee SY, Faghri F, Campbell RH, Zhai C, Efron MJ, et al. Big Data: Astronomical or Genomical? PLoS Biol 2015;13(7):e1002195. https://www.ncbi.nlm.nih.gov/pubmed/26151137.
