# Supplementary material for: VariantSpark: Cloud-based machine learning for association study of complex phenotype and large-scale genomic data
Source: Gigascience. 2020 Aug 6;9(8):giaa077. doi: 10.1093/gigascience/giaa077 (PMC7407261; doi:10.1093/gigascience/giaa077)
Supplement: giaa077_GIGA-D-19-00335_Revision_1 [file giaa077_giga-d-19-00335_revision_1.pdf]

GigaScience

VariantSpark: Cloud-Based Machine Learning for Association Study of Complex Phenotype and Large-Scale Genomic Data

--Manuscript Draft--

**Manuscript Number:**	GIGA-D-19-00335R1
**Full Title:**	VariantSpark: Cloud-Based Machine Learning for Association Study of Complex Phenotype and Large-Scale Genomic Data
**Article Type:**	Technical Note
**Funding Information:**	
**Abstract:**	Background: Many traits and diseases are thought to be driven by more than one gene (polygenic). Polygenic Risk Scores (PRS) hence expand on Genome-Wide Association Studies (GWAS) by taking multiple genes into account when building risk models. However, PRS only considers the additive effect of individual genes but not epistatic interactions or the combination of individual and interacting drivers. While evidence of epistatic interactions are found in small datasets, large datasets have not been processed yet due to the high computational complexity of the search for epistatic interactions.<br><br>Finding: We have developed VariantSpark, a distributed machine learning framework able to perform association analysis for complex phenotypes that are polygenic and potentially involve a large number of epistatic interactions. Efficient multi-layer parallelization allows VariantSpark to scale to whole-genome of population-scale datasets with a hundred million genomic variants and hundred thousand samples.<br><br>Conclusions: Compared to traditional GWAS, VariantSpark better identifies genomic variants associated with complex phenotypes. VariantSpark is 3.6 times faster than ReForeSt and the only method able to scale to ultra-high dimensional genomic data in a manageable time.
**Corresponding Author:**	Arash Bayat<br><br>AUSTRALIA
**Corresponding Author Secondary Information:**	
**Corresponding Author's Institution:**	
**Corresponding Author's Secondary Institution:**	
**First Author:**	Arash Bayat
**First Author Secondary Information:**	
**Order of Authors:**	Arash Bayat
	Piotr Szul
	Aidan R. O'Brien
	Robert Dunne
	Brenden Hosking
	Yatish Jain
	Cameron Hosking
	Oscar J. Luo
	Natalie Twine
	Denis Bauer

Powered by Editorial Manager® and ProduXion Manager® from Aries Systems Corporation

Order of Authors Secondary Information:	
Response to Reviewers:	Letter to the editor and response to reviewers are attached as a supplementary data for reviewers only (LetterToEditorAndResponseToReviewers.pdf)<br>Proof of compile is provided as supplementary data for reviewers only (ProofOfCompile.pdf)
Additional Information:	
Question	Response
Are you submitting this manuscript to a special series or article collection?	No
**Experimental design and statistics**<br><br>Full details of the experimental design and statistical methods used should be given in the Methods section, as detailed in our Minimum Standards Reporting Checklist. Information essential to interpreting the data presented should be made available in the figure legends.<br><br>Have you included all the information requested in your manuscript?	Yes
**Resources**<br><br>A description of all resources used, including antibodies, cell lines, animals and software tools, with enough information to allow them to be uniquely identified, should be included in the Methods section. Authors are strongly encouraged to cite Research Resource Identifiers (RRIDs) for antibodies, model organisms and tools, where possible.<br><br>Have you included the information requested as detailed in our Minimum Standards Reporting Checklist?	Yes
**Availability of data and materials**<br><br>All datasets and code on which the conclusions of the paper rely must be either included in your submission or deposited in publicly available repositories (where available and ethically	Yes

appropriate), referencing such data using a unique identifier in the references and in the “Availability of Data and Materials” section of your manuscript.<br><br>Have you have met the above requirement as detailed in our Minimum Standards Reporting Checklist?	

LaTeX - Main Document (TeX file) Click here to access/download;LaTeX - Main Document (TeX file);main.tex

```
This is pdfTeX, Version 3.14159265-2.6-1.40.19 (TeX Live 2018/W32TeX)
(preloaded format=pdflatex 2018.7.12)  1 MAY 2020 22:34
entering extended mode
 restricted \write18 enabled.
 %&-line parsing enabled.
**main.tex
(./main.tex
LaTeX2e <2018-04-01> patch level 5

! LaTeX Error: File `oup-contemporary.cls' not found.

Type X to quit or <RETURN> to proceed,
or enter new name. (Default extension: cls)

Enter file name:
! Emergency stop.
<read *>

l.2 ^^M

*** (cannot \read from terminal in nonstop modes)


Here is how much of TeX's memory you used:
 10 strings out of 492646
 215 string characters out of 6133325
 56649 words of memory out of 5000000
 3994 multiletter control sequences out of 15000+600000
 3640 words of font info for 14 fonts, out of 8000000 for 9000
 1141 hyphenation exceptions out of 8191
 10i,0n,8p,56b,8s stack positions out of 5000i,500n,10000p,200000b,80000s
!  ==> Fatal error occurred, no output PDF file produced!
```

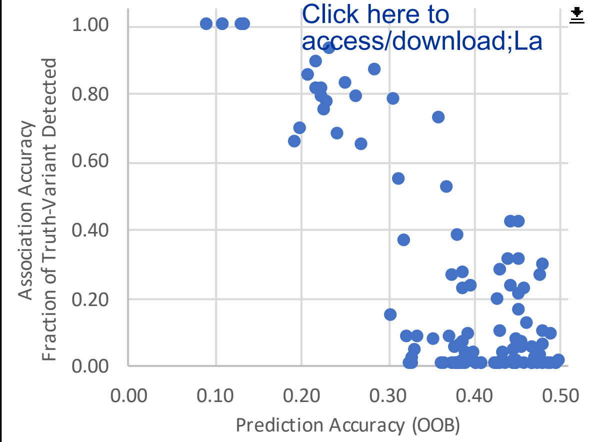

Click here to access/download;La
Association Accuracy
Fraction of Truth-Variant Detected
1.00
0.80
0.60
0.40
0.20
0.00
0.00
0.10
0.20
0.30
0.40
0.50
Prediction Accuracy (OOB)

Click here to access/download;La

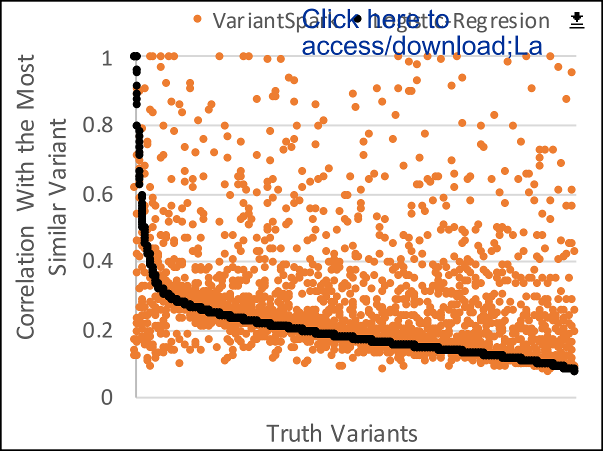

Click here to access/download;LaTeX - Figure (eps, ps, etc.);Fig-ExVar.pdf

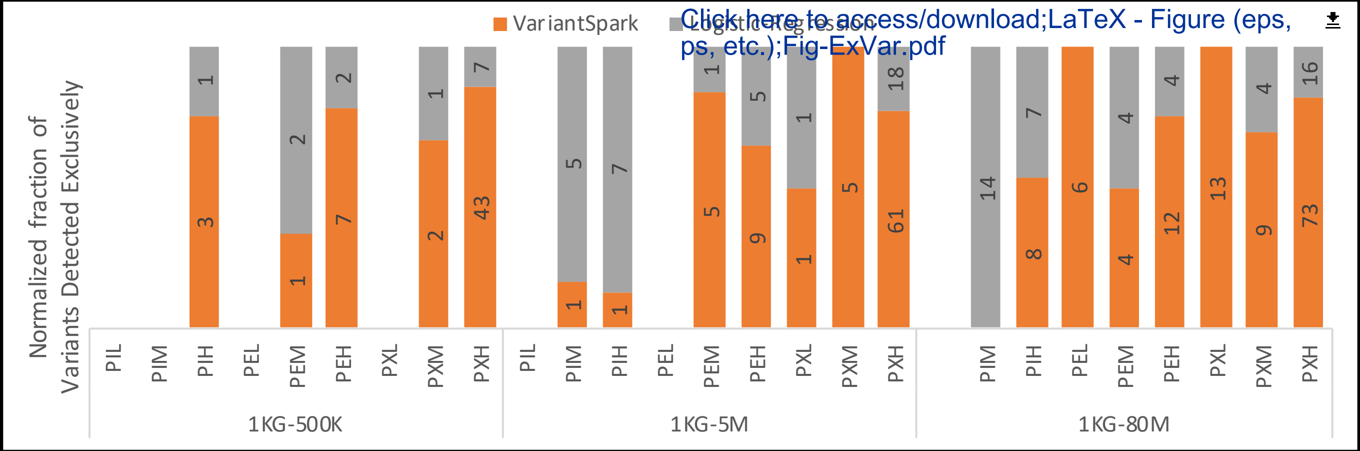

Click here to access/download;LaTeX - Figure

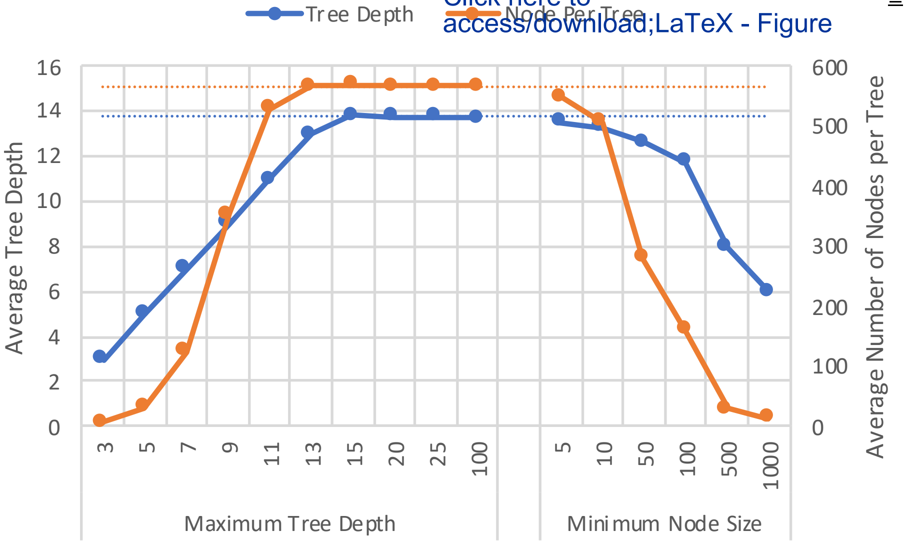

Click here to access/download;LaTeX - Figure

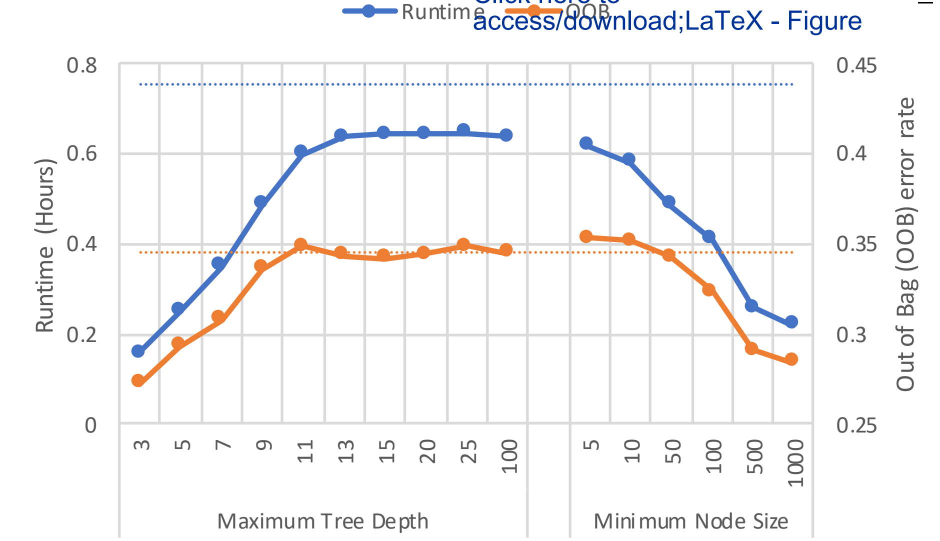

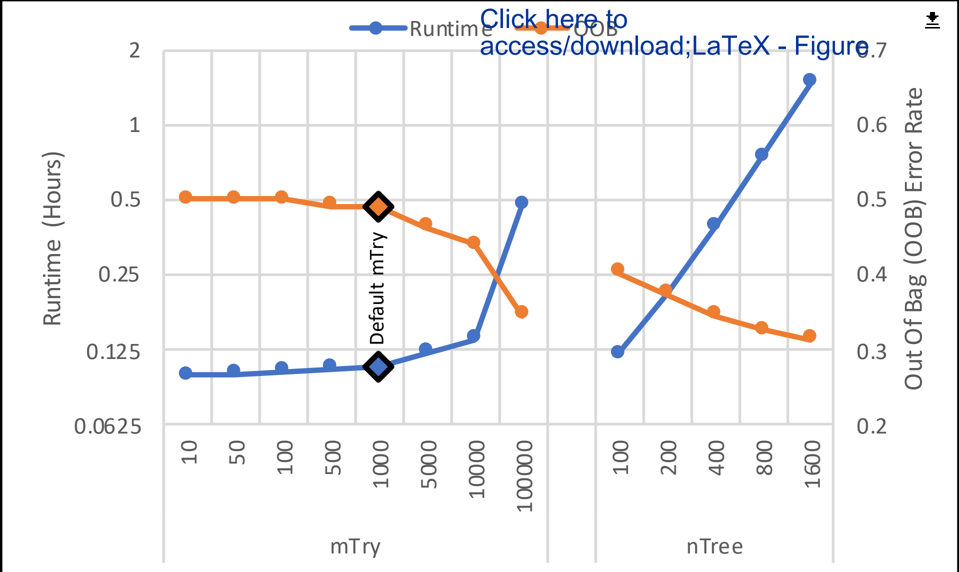

Runtime
OOB
Click here to access/download;LaTeX - Figure
2
1
0.5
0.25
0.125
0.0625
Runtime (Hours)
0.7
0.6
0.5
0.4
0.3
0.2
Out Of Bag (OOB) Error Rate
Default mTry
10
50
100
500
1000
5000
10000
100000
100
200
400
800
1600
mTry
nTree

Click here to access/download;LaTeX - Figure (eps, ps, etc.);FIG-Ranked-1.pdf

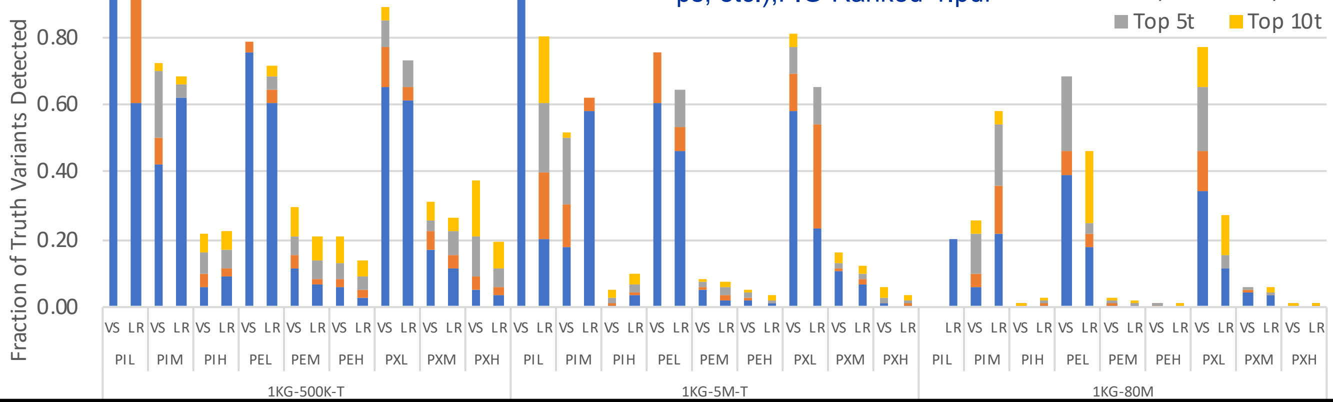

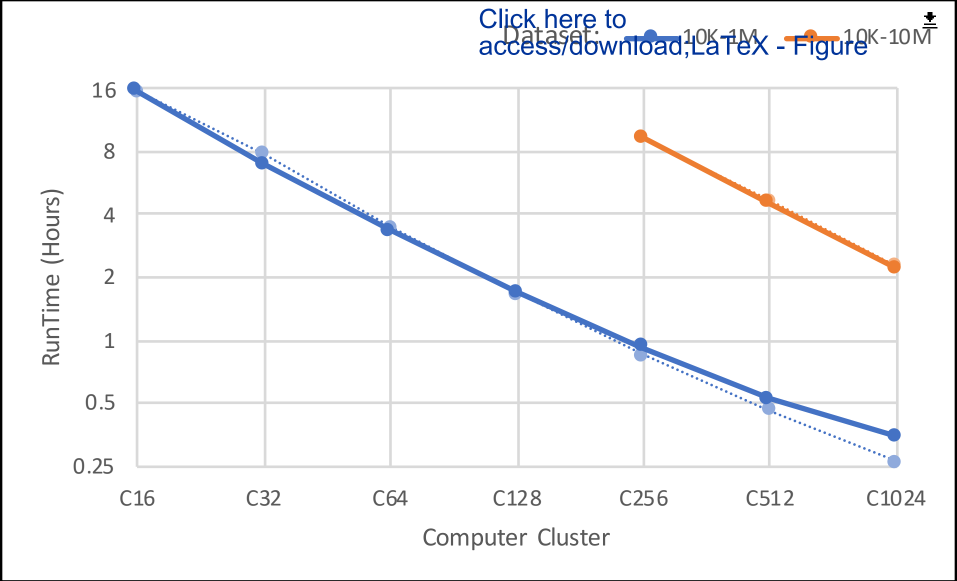

Click here to access/download;LaTeX - Figure
Dataset:
10K-1M
10K-10M
RunTime (Hours)
16
8
4
2
1
0.5
0.25
C16
C32
C64
C128
C256
C512
C1024
Computer Cluster

Click here to access/download;LaTeX - Figure

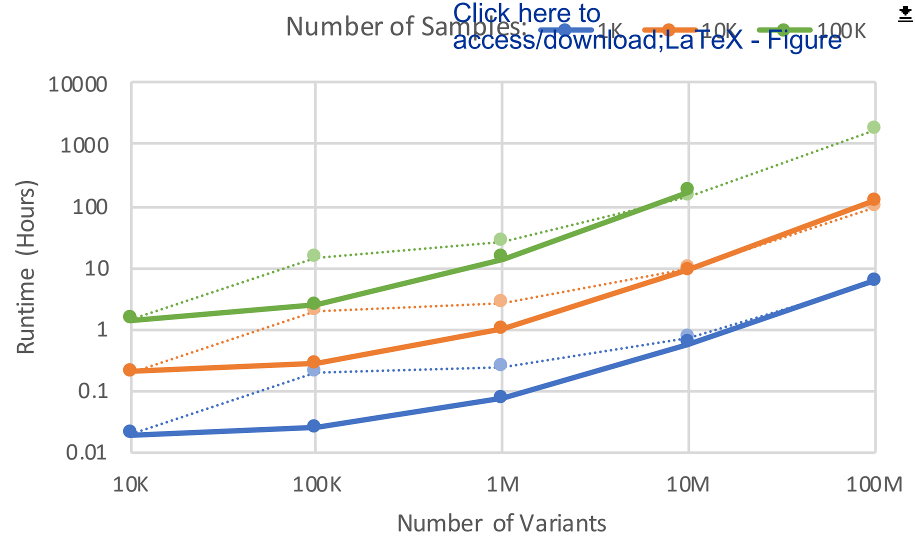

Click here to access/download;LaTeX - Figure

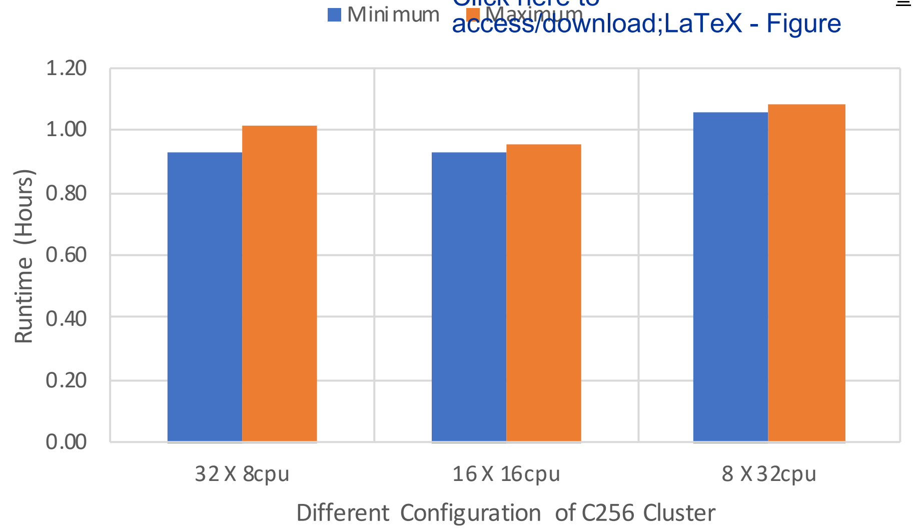

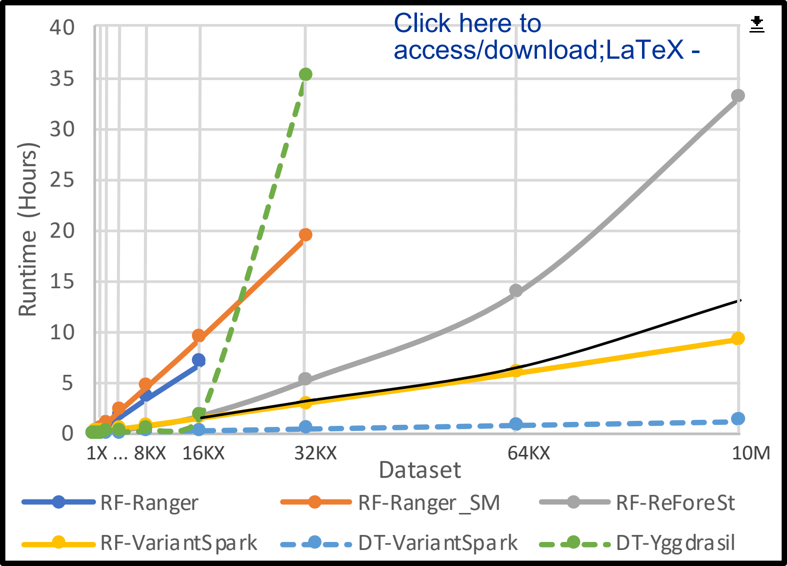
Click here to access/download;LaTeX -
40
35
30
25
20
15
10
5
0
Runtime (Hours)
1X ... 8KX
16KX
32KX
64KX
10M
Dataset
RF-Ranger
RF-Ranger_SM
RF-ReForeSt
RF-VariantSpark
DT-VariantSpark
DT-Yggdrasil

Proof Of Compile
Click here to access/download;Manuscript;ProofOfCompile.pdf

GigaScience, 2017, 1–11

doi: xx.xxxx/xxxx
Manuscript in Preparation
Paper

PAPER

VariantSpark: Cloud-Based Machine Learning for Association Study of Complex Phenotype and Large-Scale Genomic Data

Arash Bayat[1*], Piotr Szul[2], Aidan R. O'Brien[1], Robert Dunne[2], Brendan Hosking[1], Yatish Jain[1], Cameron Hosking[1], Oscar J. Luo[3], Natalie Twine[1] and Denis C. Bauer[1,4*]

[1]Commonwealth Scientific and Industrial Research Organisation (CSIRO), Health and Biosecurity, Australia and [2]Commonwealth Scientific and Industrial Research Organisation (CSIRO), Data61, CSIRO, Australia and [3]Jinan University, Department of Systems Biomedical Sciences School of Medicine, Guangzhou, China and [4]Macquarie University, Department of Biomedical Sciences, Macquarie Park, Australia

[*]arash.bayat@csiro.au, denis.bauer@csiro.au

Abstract

Background: Many traits and diseases are thought to be driven by more than one gene (polygenic). Polygenic Risk Scores (PRS) hence expand on Genome-Wide Association Studies (GWAS) by taking multiple genes into account when building risk models. However, PRS only considers the additive effect of individual genes but not epistatic interactions or the combination of individual and interacting drivers. While evidence of epistatic interactions are found in small datasets, large datasets have not been processed yet due to the high computational complexity of the search for epistatic interactions.
Finding: We have developed VariantSpark, a distributed machine learning framework able to perform association analysis for complex phenotypes that are polygenic and potentially involve a large number of epistatic interactions. Efficient multi-layer parallelization allows VariantSpark to scale to whole-genome of population-scale datasets with a hundred million genomic variants and hundred thousand samples.
Conclusions: Compared to traditional GWAS, VariantSpark better identifies genomic variants associated with complex phenotypes. VariantSpark is 3.6 times faster than ReForeSt and the only method able to scale to ultra-high dimensional genomic data in a manageable time.

Key words: GWAS, Polygenic, Epistasis, Machine Learning, Cloud Computing, Polygenic Risk Scores, Random Forest, Ultra-high dimensional

Compiled on: May 2, 2020.
Draft manuscript prepared by the author.

Key Points

- With the increasing evidence of polygenic and epistatic phenotype, there is a need for more complex association analysis.
- Random-Forests can find complex associations, but standard implementations do not scale to genomic data.
- Distributed computing overcomes this limitation but hyper-parameter tuning is critical to achieving optimal results.

Finding

Traditional genome-wide association studies (GWAS) evaluate genomic variants (a variant for short) across the genomes of many samples for statistical association with the phenotype in question. These studies are aimed at detecting variants associated with common and complex traits and diseases, such as heart disease, diabetes, and height [1]. GWAS has been successful at identifying over 50,000 associated variants in thousands of complex phenotypes (GWAS catalog [2]). Yet, many phenotypes with genetic components remain only partially explained by genetics, the so-called *missing heritability* problem [3].

One possible explanation is that these phenotypes are driven by an additive effect of several variants (**polygenic phenotype**), resulting in a small association power for each variant [4, 5, 6, 7]. Polygenic Risk Score (PRS) takes into account this additive effect to compute the genomic risk factor for a trait [8]. PRS refers to a range of statistical methods that consider the GWAS association power as a weight for the variant. Given the weight and the risk allele for each variant, PRS computes the genomic risk factor for a given sample [9]. For many phenotypes, PRS is shown to be a more accurate predictor of risk than single variants alone [10], which statistically supports the idea of the phenotype being polygenic.

Another explanation is the existence of epistatic interaction (interaction for short) between sets of variants [11] (**epistatic phenotype**). In an interaction, the combination of two or more variants highly correlate with the phenotype but individual variants do not show a strong correlation with the phenotype. Thus the phenotype can not be explained by the individual variants. Variant interactions remain invisible to traditional GWAS and subsequently to PRS methods. Several algorithms have been developed to speed up the search for the interactions [12, 13] and they have been successful to identify significant statistical interactions [14]. There is also evidence that interactions are biologically relevant [15]. However, the high computational complexity of these methods prevents them from being applied to whole-genome data. Pruning the dataset is an option but does not guarantee to preserve all the interacting variants.

Given that there is statistical proof for the existence of both polygenic phenotype and epistatic phenotype, there is a likelihood of a **complex phenotype** to exist. A phenotype that is driven by several variants individually as well as several sets of interactive variants. A novel association approach is hence needed to take into account the individual variant association power as well as the association power driven by the interactive variants. Furthermore, such a methodology needs to be applicable to genomic-scale data. Taking all variants into account reduces the chance of missing important interactions. Note that the association of interactive variant is only visible when all of them are combined. The computational complexity of such analyses made them infeasible in the past, however, combining more efficient algorithms with parallel computing resources has opened up a new avenue.

One promising algorithm to use is Random-Forest (RF) [16], which is a machine learning approach used in many modern bioinformatics analyses [17] including genomics [18, 19, 20]. It is designed to identify interactions between the given features (variants in the context of GWAS) and incorporate them into a prediction model. RF also computes a metric for each variant called *importance-score* that is an indicator of the association power for a variant. Importance-score combines individual and interaction association power into a single value. Thus RF is a perfect candidate for the association study of a complex phenotype. The randomness in the RF model is the key to avoid over-fitting, making it a robust method. Unlike black-box models such as Deep-Learning [21], the RF model is readable and can be used to extract important rules and identify interactive features. Even though RF is not a deterministic algorithm, it is an accurate approximation with a manageable computational requirement.

There are two layers of parallelization to speedup an algorithm: multi-threading and distributed computing. The former is a common approach that allows programmers to employ all processors and memory available in a single computer, usually a High-Performance Computer (HPC). The later allows a program to be executed in parallel on multiple independent computers connected by a network (known as *computer-cluster* or cluster for short). Given that the network is far slower than processors and memory, it is critical to implement the program in a way that reduces network operation and avoid a potential bottleneck. Apache Spark [22] (Spark for short) is a widely used platform for distributed computing. Distributed computing is a potential solution [23] to overcome the ever-increasing genomic data, exceeding astronomical data in volume [24].

Here, we introduce VariantSpark, a Spark-based software for association study of complex phenotypes and genomic-scale datasets. VariantSpark is the first publicly available distributed implementation of RF with the following features to reduce networking, to maximize resource utilization, and to suit genomic datasets:

- Vertical data partitioning
- Processing multiple nodes of multiple trees in parallel
- Efficiently storing genomic data in fast and a low-level Spark memory structure (Resilient Distributed Dataset or RDD for short)

VariantSpark is compatible with standard genomic data formats (e.g. VCF), and is integrated with Hail [25] to offer a range of other standard genomic analyses in a distributed manner. To assess VariantSpark's capability we compare it against the state-of-the-art bioinformatics implementation of RF as well as the latest application-agnostic distributed implementations of RF. Ranger [26] is one of the fastest multi-threaded RF, written in C++. As reported by its developer, Ranger is 180 times faster than the parallel version of the widely used *randomForest* R package [27] and requires 3.5 times less memory. It is also 2.2 and 2.6 times faster than randomForestSRC [28] and Random-

Table 1. Nine different phenotype simulated with PEPS

Phenotype Name	Phenotype Category	Number of n-way Truth Variables					Total Number of Truth	
		1-way	2-way	3-way	4-way	5-way	Variables	Variants
PIL	PI	5	0	0	0	0	5	5
PIM		50	0	0	0	0	50	50
PIH		500	0	0	0	0	500	500
PEL	PE	0	2	2	2	2	8	28
PEM		0	20	20	20	20	80	280
PEH		0	50	50	50	50	200	700
PXL	PX	5	3	2	1	1	12	26
PXM		50	25	17	13	10	115	253
PXH		500	250	167	125	100	1142	2501

Jungle [29] respectively. Ranger also implements a save-memory mode that is 1.6 times slower than normal mode but requires half the memory. To the best of our knowledge, no other multi-threaded RF claimed to be faster than Ranger. Despite this, processing data from whole-genome sequencing [30] remains practically impossible using this method. RF needs to maintain the complete dataset decompressed in memory. So a dataset of 100 million variants and 10 thousand samples requires 1 terabytes of memory (assuming 1 byte per genotype), which is unlikely available on standard HPC.

A cluster, on the other hand, can easily scale to hold hundreds of terabytes of data (as most cloud providers can supply). The most popular distributed implementation of RF is Google's PLANET [31], which is integrated into the Spark machine learning library (MLlib) [32]. PLANET uses horizontal partitioning, which is a parallelization along the wrong dimension for high dimensional genomic data. PLANET is faster than randomForest R package, with comparisons to other implementations provided in [33]. ReForeSt [34] is, to the best of our knowledge, the fastest distributed implementation of RF and is up to 3 times faster than MLlib (PLANET). ReForeSt uses similar partitioning as in Spark MLlib and extends a machine learning benchmark study [35] and was shown to be faster than XGBOOST [36] and H2O [37] for the largest dataset in the study (10M) [38]. Parallel Random-Forest (PRF) [39] is another distributed RF that takes a vertical partitioning approach and claims to be twice as fast as MLlib. The implementation has not been released and hence could not be included in our comparison. The only other relevant distributed algorithm with vertical partitioning is Yggdrasil [40]. Yet, Yggdrasil is limited to Decision-Tree [41] (DT) and does not expand to build an RF mode. However, none of these tools were tested in ultra-high dimensional data, which we define as datasets with more than 10M features.

Here, we first compare the performance of VariantSpark with the approach used in traditional GWAS, Logistic-Regression (LR) [42]. We consider various simulated phenotype, including complex phenotype, and different-sized datasets, to compare the tools' ability to detect associated variants. Then we compare VariantSpark's runtime with Ranger, ReForeSt, and Yggdrasil. Finally, we demonstrate the scalability of VariantSpark and evaluate sensitivity to hyper-parameter choices.

Datasets

Two different sets of synthetic datasets are used in this study all of which are publicly available for the replication of this study (see *Supplementary Data File 4*). The first set uses real genotypes taken from 1000-Genomes (*1KG*) Project [43] and a simulated phenotype made by Polygenic Epistatic Phenotype Simulator (PEPS) [44]. In the second set, both genotype and phenotype are simulated by VariantSpark's embedded simulator. The phenotype is a function of five randomly selected variants and a given noise parameter.

Real Genotype and Simulated Phenotype: We use these datasets to compare the accuracy of VariantSpark with Logistic-Regression. A set of phenotypes are simulated for 1KG samples using PEPS that uses real genotype data and simulates a binary phenotype associated with a subset of randomly selected variants.

PEPS first forms *n*-way truth-variables which are used to simulate the phenotype. A variable could be an individual variant (1-way variable) or set of *n* variants with epistatic interaction (*n*-way variable), so 2-way variables are pairwise epistatic interactions; 3, 4 and 5-way variables are higher-order epistatic interactions. Each variant is involved in only one variable. Variants involved in truth-variables (associated with the phenotype) are called truth-variants and are to be discovered by VariantSpark or Logistic-Regression.

Table 1 lists nine PEPS simulated phenotypes (provided in *Supplementary Data File 3*) in three categories: PI, PE, and PX. PI phenotypes are made of only 1-way variables (**I**ndividual variant). PE phenotypes are made of 2-way or higher-order variables (**E**pistatic variables only). PX phenotypes include epistatic and individual variables (comple**X** phenotype). In each category, there are three phenotypes with Low (L), Moderate (M), and High (H) number of truth-variants.

The 1KG dataset consists of 2,504 samples and about 80M variants with multi-allelic variants converted to multiple bi-allelic variants. We generate four subsets this data by randomly selecting variants, two by adding the truth-variants of all phenotypes back if they were removed by this process (see Table 2).

Simulated Genotype and Simulated Phenotype: These datasets, listed in Table 3, are used for the runtime analysis of VariantSpark. We start from 1,000 samples and 10,000 variants and increase the number of samples or variants 10 times at each step to reach either 100,000 samples and 10,000,000 or 10,000

Table 2. 1000-Genome dataset and its subsets. There are 2504 samples in these dataset.

Dataset	# variants	% of Truth variants Included
1KG-80M	81,647,203	100
1KG-5M	5,000,516	6.1
1KG-500K	500,446	0.6
1KG-5M-T	5,016,789	100
1KG-500K-T	517,729	100

Table 3. Synthetic datasets generated by VariantSpark.

Dataset	Size	Number of Samples (nS)	Variants (nV)	Genotypes $nS \times nV$
1K-10K	10M	1,000	10,000	1e7
1K-100K	100M	1,000	100,000	1e8
1K-1M	1B	1,000	1,000,000	1e9
1K-10M	10B	1,000	10,000,000	1e10
1K-100M	100B	1,000	100,000,000	1e11
10K-10K	100M	10,000	10,000	1e8
10K-100K	1B	10,000	100,000	1e9
10K-1M	10B	10,000	1,000,000	1e10
10K-10M	100B	10,000	10,000,000	1e11
10K-100M	1T	10,000	100,000,000	1e12
100K-10K	1B	100,000	10,000	1e9
100K-100K	10B	100,000	100,000	1e10
100K-1M	100B	100,000	1,000,000	1e11
100K-10M	1T	100,000	10,000,000	1e12

Table 4. Datasets for high-resolution comparison of the VariantSpark runtime with other implementations of RF. X represents 100 and KX represents 102,400. 10M is identical to the 10K-10M dataset. All dataset includes 10,000 samples.

Dataset	# variants (nV)	Dataset	# variants (nV)
1X	100	256X	25,600
2X	200	512X	51,200
4X	400	1KX	102,400
8X	800	2KX	204,800
16X	1,600	4KX	409,600
32X	3,200	8KX	819,200
64X	6,400	16KX	1,638,400
128X	12,800	32KX	3,276,800
256X	25,600	64KX	6,553,600
512X	51,200	10M	10,000,000

samples and 100,000,000 variants. These genotypes are simulated with random distribution of phenotypes using VariantSpark *gen-features* command.

The phenotype is simulated using VariantSpark *gen-label* commands and based on five randomly selected variants all with equal contributions (all weight are set to 1.0). To make a more complex phenotype, the mean and the standard deviation of the noise, *-gm* and *-gs* parameters respectively, are both set to 0.5. The fraction of noise variants, *-gvf* parameter, is set to $\frac{100}{nV}$ to include 100 noise variants (randomly selected from the variants in the dataset).

For the comparison to other tools, we subset variants from the 10K-10M dataset and include the 5 truth-variants in all subsets. We start from 100 variants and double it at each step. These datasets are listed in Table 4.

Compute resources

For reproducibility, all tests are performed on Amazon Web Service (AWS) compute resources. We use AWS EC2 (Elastic Compute Cloud) and EMR (Elastic Map Reduce) for HPC and cluster compute respectively. We use clusters of different sizes listed in Table 5. For all clusters, the master-node is an r4.2xlarge EC2 instance with 8 vCPU and 61GB of memory. Compute-nodes are r4.4xlarge with 16 vCPU and 122GB of memory except for C256-S and C256-L where we use r4.2xlare and r4.8xlarge EC2 instances as compute-nodes. The r4.8xlarge has 32 vCPU and 244GB of memory.

Table 5. EMR clusters and compute-nodes.

Cluster	Compute-Nodes			Master+Compute vCPU	Master+Compute Memory(GB)
C16	1	×	r4.4xlarge	8+16	61+122
C32	2	×	r4.4xlarge	8+32	61+244
C64	4	×	r4.4xlarge	8+64	61+488
C128	8	×	r4.4xlarge	8+128	61+976
C256	16	×	r4.4xlarge	8+256	61+1952
C512	32	×	r4.4xlarge	8+512	61+3904
C1024	64	×	r4.4xlarge	8+1024	61+7808
C256-S	32	×	r4.2xlarge	8+256	61+1952
C256-L	8	×	r4.8xlarge	8+256	61+1952

Experimental Setup

The combination of nine phenotypes described in Table 1 and five genotype dataset described in Table 2 result in $9 \times 5 = 45$ case/control datasets, which we process with both VariantSpark and Logistic-Regression (LR) *wald* test implemented in Hail. We pass the first two PCA (Principle Component Analysis) vectors as co-variate to LR. VariantSpark uses the following parameters for this experiment $nTree = 1000$, $mTry = 0.1 \times nV$, $maxD = 15$ and $minNS = 50$.

We ranked the variants biased on p-value computed by LR and importance-score computed by VariantSpark. We replicate the experiments three times (similar phenotypes are simulated but different randomly selected truth-variants are used to form phenotype). In the last two replicate, we did not process 1KG-80M dataset with VariantSpark due to high computational cost. Due to technical issues, the VariantSpark results for PIL on 1KG-80M was missed for the first replicate.

VariantSpark and ReForeSt are executed on C256 cluster while Ranger is executed on r4.16xlarge computer with 64 vCPU and 488GB of memory, which is the practical limit of HPC. We apply $maxD = 15$ and $minNS = 50$ where applicable and build 1000 trees ($nTree = 1000$) with $mTry = 0.1 \times nV$. We build a Decision-Tree with Yggdrasil 10 times. For VariantSpark we build a forest with 10 tree and set $mTry = nV$, as this parameter setting mimics growing a Decision-Tree.

When testing VariantSpark's scalability the following parameters were applied to all experiment below, unless mentioned otherwise: $maxD = 15$, $minNS = 50$, $mTry = 0.1 \times nV$ and $rbs = 100$ (grow 100 trees in parallel).

- *Dataset Size:* The expected runtime to build 1000 trees for all datasets in Table 3 on C256 cluster. Since the actual runtime is too high for larger datasets we build less trees (i.e. 500, 100 or 10) and record the data load time (β) and average train time per tree (θ) reported by the VariantSpark. The expected runtime for 1000 trees is computed as $\beta + (1000 \times \theta)$. The exact number of trees for each dataset and the un-normalized runtime can be found in *Supplementary Data File 1*.
- *Cluster Size:* 500 trees are built for 10K-1M dataset on clusters of different sizes (see Table 5). We also replicate this experiment for 10 time larger dataset (10K-10M) but only on C256, C512 and C1024 clusters.
- *Compute-Node Size:* 1000 trees are built for 10K-1M dataset on C256-S, C256, and C256-L. This experiment is replicated tree times to show that VariantSpark runtime variation is negligible.
- *Batch Size (rbs):* 500 trees are build for 10K-1M dataset with *rbs* eaqul to 10, 50, 100 or 500.

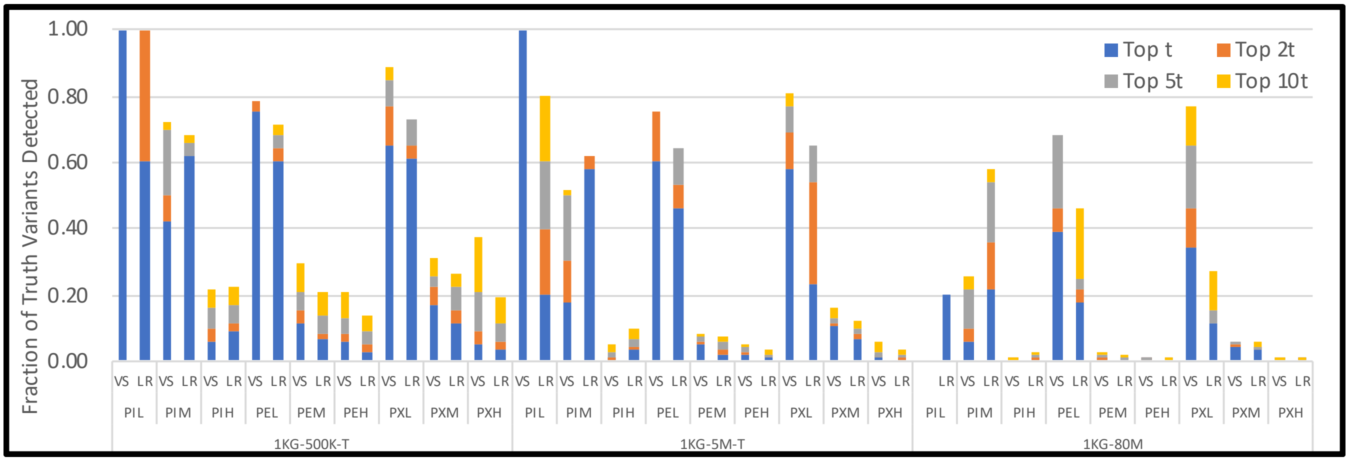

(a) Fraction of truth-variants detected in the top *t*, 2*t*, 5*t* and 10*t* top ranked-variants where *t* is the number of truth-variants. Variants are ranked by VariantSpark (VS) and Logistic-Regression (LR)

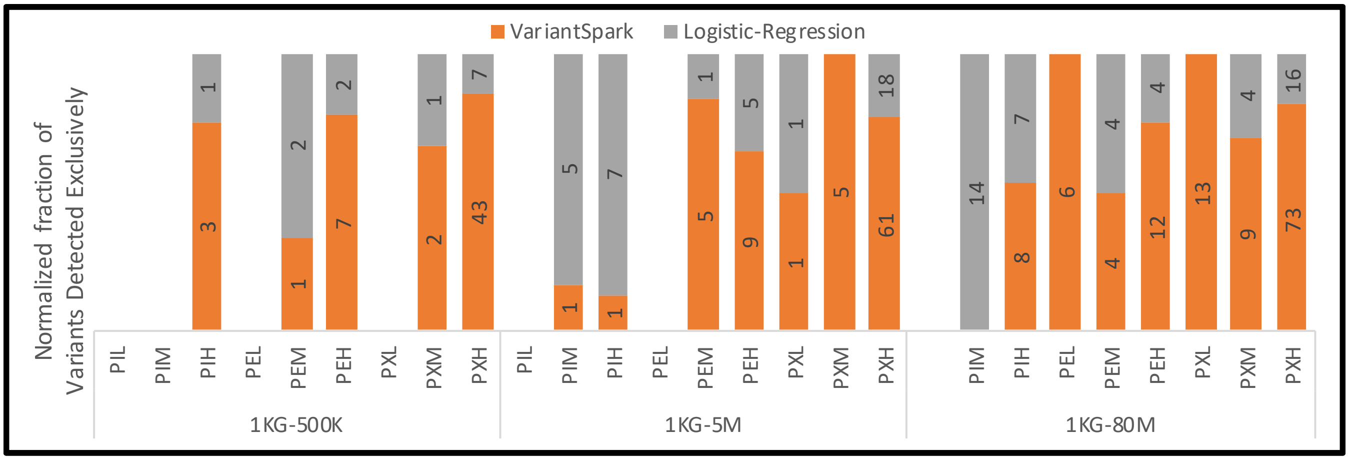

(b) Number of truth-variants exclusively detected by either VariantSpark or Logistic-Regression. Each bars is normalized to total number of exclusively detected truth-variants. Where, there is no exclusive detected truth-variants the bar is not plotted.

Figure 1. VariantSpark comparison with Logistic-Regression on their ability to detect phenotype-associated variants.

The following experiments are performed to show the effect of different parameters on the VariantSpark runtime and out-of-bag (*OOB*) error rate (prediction accuracy) when processing the 10K-1M dataset.

- *Unlimited:* 500 trees are built with no limits on the depth of the tree or the node size.
- *Maximum Depth (maxD):* 500 trees are built with no limits on the node size but the *maxD* varies as follow: 3, 5, 7, 9, 11, 13, 15, 20, 25, 100.
- *Minimum Node Size (minNS):* 500 trees are built with no limits on depth of the trees but the *minNS* varies as follow: 5, 10, 50, 100, 500, 1,000.
- *mTry*: 500 trees are built with the *mTry* varies as follow: 10, 50, 100, 500, 1,000, 5,000, 10,000, 100,000
- *nTree*: Starting from 100 trees and doubling the number of trees up to 1600 trees.

Result 1: VariantSpark detects complex genomic interactions

We compare the performance of VariantSpark with Logistic-Regression (LR) using phenotypes of different complexity and different sized datasets. First, we report how many truth-variants (TVs), i.e. variants associated with the phenotype, can be detected by the respective approaches.

Figure 1a shows the fraction of TVs found in the top *r* ranked-variants (RVs) for all phenotype categories (see Table 1) and dataset sizes. More TVs can be detected with higher value of *r* so we let *r* vary between *t*, 2*t*, 5*t* and 10*t* where *t* is the number of TVs (note, *t* is different for each phenotype). We do not consider the order of variants in the list of RVs. All experiments were replicated three times. We highlight results from the first replicate for 1KG-80M (2504 samples and 80M features), 1KG-5M-T (5M variants subset including all truth variants) and 1KG-500K-T here, but other results (second and third replicates as well as 1KG-5M and 1KG-500K subsets) are reported in *Supplementary Data File 1* and support the same conclusion.

Ideally, all TVs are expected to be listed in the top *t* RVs, resulting in a maximum value of 1. VariantSpark indeed achieves this

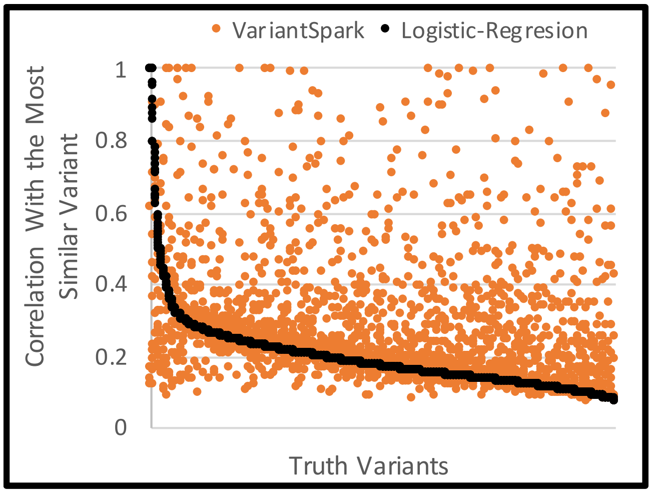

(a) The absolute value of Pearson correlation coefficient for each of the truth-variants and the most correlated variants in the top 10*t* ranked-variants

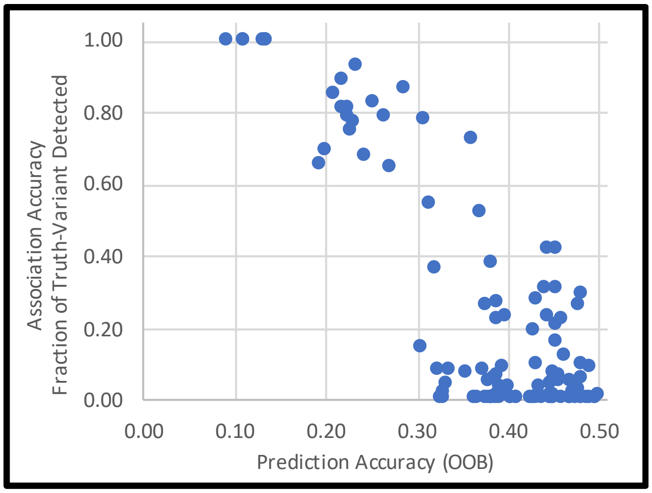

(b) Comparing association accuracy (fraction of detected TV) with prediction accuracy for all experiments shown in Figure 1a.

Figure 2. Comparison of exclusively detected variants and correlation with prediction accuracy.

for two datasets (1KG-500K-T, 1KG-5M-T) and **P**henotypes with **L**ow numbers of **I**ndividually associated variables (PIL). Logistic-Regression (LR), on the other hand, only detects all TVs in the smaller of these two datasets and only when expanding the list to the top 2*t* RVs.

More generally, VariantSpark detects either more TV or an equivalent proportion for most phenotypes and data set sizes. VariantSpark especially outperforms LR for **E**pistatic (PE) and comple**X** (PX) phenotypes where interactions are involved (achieving scores up to X times better than LR). This is because the association power gained by the interaction between variants remain invisible to LR.

Conversely, LR performs at most 2.2 times better than VariantSpark on datasets with **M**oderate and **H**igh numbers of **I**ndividual TVs (PIM and PIH). This gain over VariantSpark is likely due to the need to tune hyper-parameter choices for each dataset, which has resulted in non-optimal performance in these instances (see Hyper-Parameter Tuning section).

For phenotypes with a high number of TVs (i.e. PIH, PEH, and PXH) the detection rate is low for both VariantSpark and LR, especially in case of the largest dataset (1KG-80M). For such complex phenotypes, detecting all TVs, even in the top 10*t* RVs, is a difficult task.

Figure 2a illustrates a more in depth comparison of VariantSpark and LR processing the 1KG-80M dataset with PXH phenotype. Note, in this dataset the truth variables are not necessarily present, reflecting a more realistic scenario of associated variants being filtered out by various pre-processing and quality control steps. We hence perform a more qualitative analysis by considering the detection of variants that correlate with TVs (i.e. variants in the same haplotype as a TV). The horizontal axis lists all TVs even if they were not included in the dataset. For each TV we look for the most correlated variant in the top 10*t* RVs and plot the maximum absolute value of Pearson correlation coefficient (γ). High γ indicates that the detected variant highly correlates the TV and possibly identify the same genomic region as the TV. TVs are sorted based on their LR γ. The γ value for all experiments and both methods are listed in *Supplementary Data File 2*.

As shown in Figure 2a while LR quickly exhausts its ability to detect the TV or equivalent variants (γ drops below 0.5), VariantSpark's γ stays above 0.75 for more variants. We define the number of exclusively detected TVs by VariantSpark as the number of TVs where the VariantSaprk γ is above 0.75 and LR γ is below 0.5. The number of exclusively detected variants by LR is defined similarly. We quantify the number of exclusively detect variants by either method on 1KG-500K and 1KG-5M datasets. As shown in Figure 1b the number of exclusively detected TVs by VariantSpark is up to 4.6 times higher than LR (if both VariantSpark and LR detect TVs exclusively). Complete numerical comparison including for 1KG-5M-T and 1KG-500K-T datasets and the other two replicates are provided in *Supplementary Data File 1*.

It is worth noting that association accuracy, i.e. the ability to recover TV, is distinct from prediction accuracy, i.e. predicting the correct label for a sample. As shown in Figure 2b, prediction accuracy shows only a moderate correlation with association accuracy (correlation coefficient equal to -0.84). This is because a sufficiently large feature set can create a model that can predict the label by chance, while choosing the TV is a less stochastic process, as demonstrated by the larger value range on the vertical axis. When finding disease genes where the TVs are unknown, using the prediction accuracy to the known labels can only be used as a rough proxy.

Result 2: VariantSpark outperforms state-of-the-art HPC and distributed implementations

We benchmark VariantSpark against the fastest HPC and distributed implementation of RF, respectively: Ranger and ReForeSt. We record the runtime of all three tools on synthetic datasets with 10,000 samples and doubling the number of variants, starting from 100 to 6.5 million and then 10 million.

Figure 3 shows that only VariantSpark and ReForeSt were able to process the two largest datasets. Ranger fails to process datasets larger than 1.6M variants, and while in *save-memory* mode, it processes up to 3.2M it does so 1.4 times slower than in

normal mode. Ranger is executed on a computer with 488GB of memory. Yet it could not process dataset larger than 3.2M due to excessive memory usage. Note that the biggest dataset in the comparison (10M x 10K) has 100 billion genotypes, which can be loaded into 100GB of memory, and VariantSpark processes it with peak memory usage of 120GB.

For the largest dataset that Ranger processed (1.6M), ReForeSt and VariantSpark perform 4.1 and 4.6 times faster than Ranger, respectively. Ranger advertises a special GWAS-mode but we were unable to successfully run this mode. The quoted runtime in the Ranger publication (for 10,000 samples, 150,000 variants, and $mTry = 15,000$) shows that GWAS mode is twice as fast as normal mode and uses the same amount of memory as save-memory mode. Given this information, the GWAS-mode would not be able to process datasets with more than 3.2M variants and would remain to be slower than VariantSpark and ReForeSt.

VariantSpark and ReForeSt perform comparably for 1.6M variants (5450 and 6044 seconds respectively); After this point, the runtime of ReForeSt grows exponentially while VariantSpark grows sub-linearly. Note the thin unmarked black line illustrating a linearly runtime increase starting from the average runtime of VariantSpark and Reforest for a dataset of 1.6M variants (5747 seconds). VariantSpark is 3.6 times faster than ReForeSt processing dataset with 10M variants, a difference that increases further for larger datasets due to the exponential vs sub-linear runtime behavior.

We also compare VariantSpark with Yggdrasil, as the only other vertical partitioning implementation. As Yggdrasil only builds a single Decision-Tree, we run VariantSpark with *mTry* equal to the number of variants to emulate building a Decision-Tree and record the runtime of building Decision-Trees 10 times with each method. As shown in Figure 3, Yggdrasil's runtime increased dramatically for 3M variants and took 35 hours to complete (possibly due to excessive memory usage). VariantSpark performs 9 and 87.4 times faster than Yggrdasil for a dataset of 1.6M and 3.2M variants, respectively. As mentioned above the biggest dataset requires 100GB memory to be loaded. While Yggdrasil is executed on a computer cluster with 2TB of memory, it processes the 3.2M dataset with difficulty.

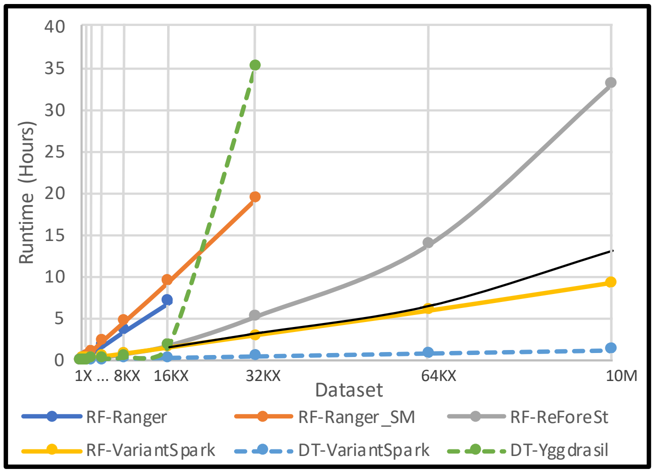

Figure 3. VariantSpark's runtime compared to other implementation of Random-Forest (RF) and Decision-Tree (DT). The RF and DT workloads are different and should not be compared together. The number of variants in the dataset is doubled at each step. The thin unmarked black line illustrating if the runtime increases linearly starting from the average runtime of VariantSpark and Reforest for a dataset of 1.6M variants.

Result 3: VariantSpark scales at most linearly with sample and variant increases

We test VariantSpark's scalability by recording the runtime when increasing the number of variants 10 times at each step, with 1K, 10K and 100K samples, respectively. As shown in Figure 4a, the runtime increases sub-linearly with the growing number of variants and increases linearly with the growing number of samples. Note that both axes are on a logarithmic scale.

VariantSpark can utilize distributed compute resources efficiently and scales linearly with the size of the cluster as shown in Figure 4b. It records the speedup gained when doubling the size of the cluster processing the 10K sample and 1M variant dataset. Up to the C512 cluster, the runtime can be halved ($speedup \sim= 2$). However, using a C1024 cluster, the speedup drops to 1.5, which is due to the 10K-1M dataset not being large enough to be efficiently partitioned over 1024 CPUs and networking becomes a bottleneck. The 2 fold speedup on this larger cluster is achieved when processing a 10 times larger dataset (10K-10M).

We also investigate whether high-performance compute-nodes perform better than commodity ones, by running the same job on three clusters of the overall same capacity but with different numbers and sizes of compute-nodes. Each cluster processes the job three times with minimum and maximum runtimes plotted. Figure 4c shows that compute-node choice has little impact on the runtime. Interestingly, the most expensive HPC computer node (8 computers each with 32 vCPU) delivered a worse runtime compared to a commodity set-up (32 computers each with 8 vCPU), with the best performance delivered by a moderate size computer (16 computers each with 16 vCPU). This is because of the balance between CPUs, memory, and network performance. The runtime variation between replicates is less than 10%, with the largest difference observed in clusters utilizing more compute-node. This is likely due to the increase in networking between nodes, which is subject to external fluctuations.

Result 4: Hyper-Parameter Tuning is different for ultra-high dimensional data

There are four important parameters to set when building an RF model:

- *nTree*: Number of trees in the forest
- *mTry*: Number of variants evaluated at each node of a tree
- *maxD*: Maximum depth of a tree to grow.
- *minNS*: Minimum number of samples in a node to be processed

Here, we show that parameter choice substantially impacts the performance and accuracy of the trained model. We varied these parameters and recorded runtime and Out-Of-Bag error rate (*OOB*). Also, we record the average number of nodes per tree and average tree depth to show the effect of *maxD* and *minNS* on the RF model.

As a rule of thumb, it is recommended to set *mTry* to $\sqrt{nV}$, however our findings shows that this recommendation does not suit the analysis of genome-wide datasets. Figure 4d shows the effect of *mTry* on the runtime and accuracy of the RF model (10K-1M dataset). $mTry = 0.1 \times nV = 100,000$ shows a substantial improvement in the accuracy compared to the previously recommended

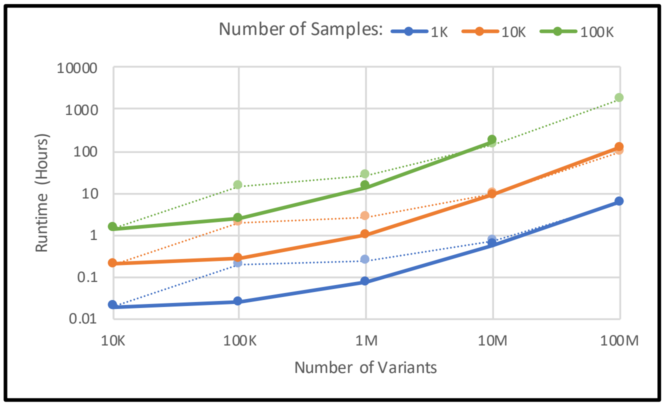

(a) VariantSpark runtime increases linearly with the number of variants and samples. For smaller datasets the runtime increases sub-linearly. The dashed lines is the expected linear increase with number of variants (the runtime of 10 times less variants multiplied by 10).

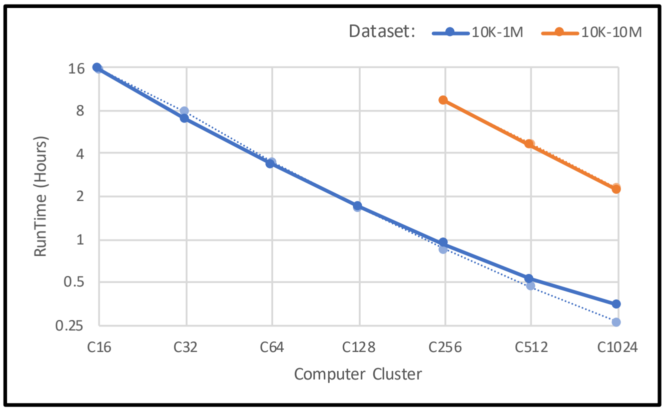

(b) VariantSpark runtime processing a small and a large datasets and doubling the size of cluster at each step. The dashed lines is the expected linear decrease with the size of cluster (the runtime of half-sized cluster divided by two).

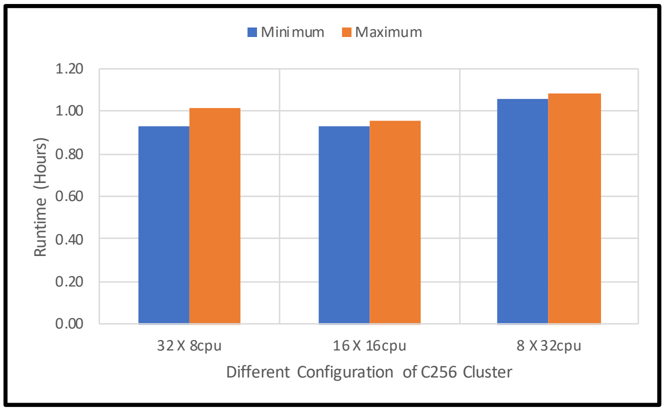

(c) The effect of cluster configuration on VariantSpark runtime. More small compute-nods on left and fewer large compute-nodes on right.

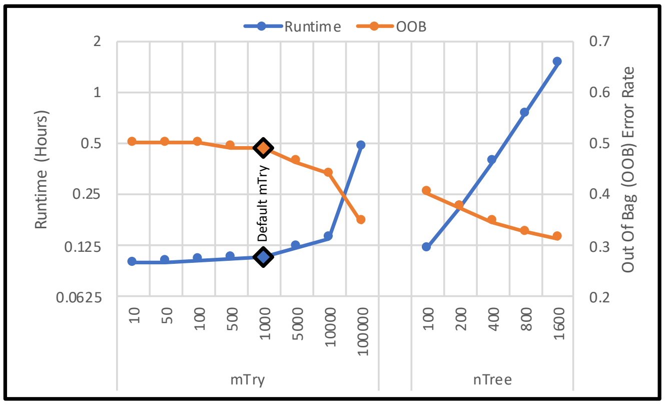

(d) The effect of *mTry* and *nTree* on the runtime and accuracy (*OOB*) of the VariantSpark. The widely used recommended *mTry* is highlighted.

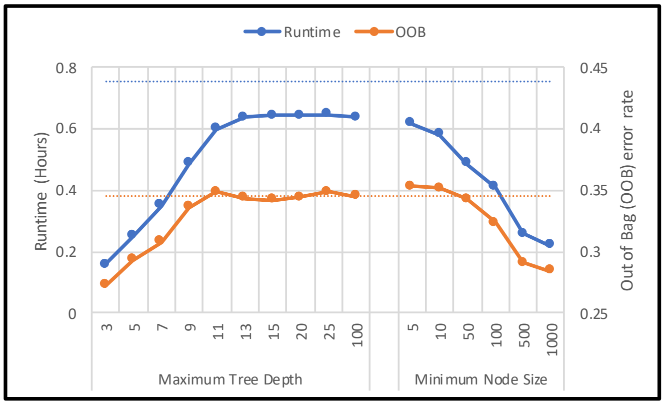

(e) The effect of *maxD* and *minNS* on the runtime and accuracy (*OOB*) of the VariantSpark. The dashed lines represent the case where no limit is applied.

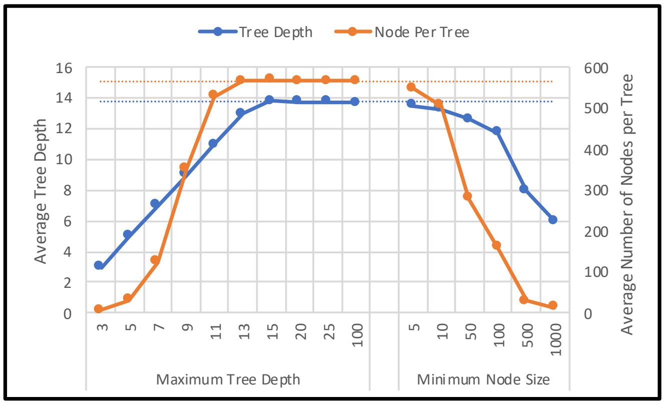

(f) The effect of *maxD* and *minNS* on the average depth and number of nodes of a tree. The dashed lines represent the case where no limit is applied.

Figure 4. VariantSpark runtime as a function of size of **(a)** the dataset **(b)** the cluster **(c)** the compute-nodes. VariantSpark runtime and accuracy as a function of **(d)** *mTry* and *nTree* **(e)** *maxD* and *minNS*. **(f)** The effect of *maxD* and *minNS* on the average depth and the number of nodes per tree.

$mTry = \sqrt{nV} = 1,000$.

Applying limits to RF training to keep trees shallow and efficient impacts the runtime and accuracy of the model. Figure 4e shows the runtime and *OOB* of VariantSpark when no limits are applied as well as when maximum depth (*maxD*) and minimum node size (*minNS*) are set. Applying these limits reduces the runtime up to 4.8 times. Interestingly, applying these limits also reduces *OOB* (increase accuracy). This is because deep down in the trees, there are fewer samples in nodes and it is more likely for a variant to gain information by chance. In other words, there

is less statistical support for the information obtained from the bottom of a deep tree and pushes the model to overfit the data. Also illustrated in this figure is the impact of *maxD* and *minNS* on runtime. Note, getting the best accuracy by setting optimal parameter values depends on the complexity of the phenotype and the size of the dataset, hence likely differs between datasets.

Figure 4d shows that the increase in the number of trees *nTree* increases the runtime linearly as expected. Also, the *OOB* is reduced (higher prediction accuracy) when doubling the *nTree* at each step. However, the reduction in *OOB* is slowed down when training excessive numbers of trees. In other words, we cannot reduce *OOB* to zero by increasing the number of trees. At this stage it is not possible to predefine the optimal number of trees as it depends on the complexity of the phenotype, and the size of the dataset.

The effect of VariantSpark batch size (number of trees processed in parallel) is recorded in *Supplementary Data File 1*. An appropriate batch size (depending on the size of cluster and networking performance) can result in the highest speedup at no cost to the accuracy.

Methods

VarintSpark is a distributed implementation of the original Random-Forest classification algorithm [16]. It accepts ordinal features and a categorical response variable. In the context of GWAS, features are genomic variants and encoded to 0, 1, and 2 for 0/0, 0/1 and 1/1 genotypes, respectively. This is the VariantSpark default encoding when the data is provided in a genomic VCF format. It is possible to use a more complex encoding in a Comma-Separated Value (CSV) format. The user can combine other ordinal omics data with genomics data for multi-omics analysis. In case/control studies, the response variable is a binary phenotype. Yet, VariantSpark can perform multi-class analysis too.

Importance-score captures interactions

When processing a node of a tree, Random-Forest evaluates randomly selected variants to separate samples of a node into two child nodes (a binary tree). The goal is to keep samples of the same class in one of the child nodes. The best-split is the variant that results in the highest separation of samples. The best-split maximizes the *information-gained*, which is a metric that measures the quality of separation. VariantSpark uses Gini-Index impurity as described in [16] to compute information-gained as used in the original Random-Forest.

The samples in each node are selected as a result of the best-split in all parent nodes. Thus the best-split and information-gained in each node depend on all variants selected in the upstream nodes (to the root) and the interaction between them. Given a large number of trees built in a Random-Forest model a variant can be selected as the best-split in various nodes in the forest. The information-gained by the variant in each of these nodes discloses part of its interactions (with variants selected in upstream nodes above it). The importance-score of a variant, computed as the average information-gained for the variant, represents all of its interactions discovered by the Random-Forest model.

Algorithmic Computational Complexity

Here we describe the theoretical dependency of VariantSpark's runtime on different parameters. The runtime of the core computation (excluding loading data to memory) is expected to be linear in $nTree \times nNode \times mTry \times nS$. The *nTree* and *mTry* are directly given by the user and represent the number of trees in the forest and the number of variables to be evaluated for each node of each tree, respectively. *nS* represents the number of samples in the dataset. To evaluate each variant at each node of a tree, the algorithm needs to loop through all samples. *nNode* represents the average number of non-leaf nodes per tree.

The value of *nNode* is determined after the Random-Forest is trained as it depends on *mTry*, *maxD*, and *minNS* as well as the complexity of the phenotype and the size of the dataset. The lower *mTry* the lower the chance for a node to divide into a pure (leaf) nodes thus the higher *nNode*. With a lower *maxD* or a higher *minNS*, trees are smaller (*nNode* is lower). Also, more samples in the dataset result in deeper trees (nodes get purer with more splits), which ultimately increases the *nNode*. If a phenotype depends only on a few strongly associated signals, trees are shallower and the *nNode* is smaller.

Given $nV = 100M$, $nS = 10K$, $nTree = 10K$, $nNode = 100$ and $mTry = 0.1 \times nV$ a computer should perform 10^{17} operations to build the RF model. This massive computational requirement indicates the importance of using a distributed computing platform for such analysis.

The number of classes in the phenotype and the number of different values a feature can take also affects the processing time. However, we did not consider their effect as for most analysis the phenotype is a binary value and bi-allelic genotypes are encoded to 0, 1, and 2. The time it takes to load data into memory is a linear function of $nS \times nV$ (number of samples and variants in the dataset, respectively).

Distributed Computing

VariantSpark is implemented on top of Apache Spark, a fast distributed computing platform. In the Spark platform, the dataset is partitioned in the memory of several computers (compute-nodes), controlled by a central computer (master-node). In most implementations of machine learning algorithms, the dataset is partitioned by samples (horizontal partitioning) such that each compute-node contains the data for all features and a set of samples. This is because most machine learning dataset includes a large number of samples and a small number of features. However, in genomic datasets, it is the number of features that outgrows the number of samples by several orders of magnitude. Partitioning by variants (Vertical Partitioning) is more effective for genomic data.

Vertical partitioning helps to reduce slow networking operations. If data is partitioned by samples, to process each node of a tree, each compute-node in the cluster must partially evaluate the selected *mTry* variants and send back results to the master node of the cluster for aggregation. However, when partitioning by variants, each compute-node evaluates a subset of the *mTry* variants (exist in its local memory) and only sends the information about the best local split to the master-node.

Another important optimization in VariantSpark is parallelizing the processing of several nodes from several trees in a batch such that network operations never become a bottleneck. Finally, VariantSpark uses Spark Resilient Distributed Datasets (RDD),

which provide the lowest level of access to the memory to deliver the highest performance.

Conclusion

While there is evidence for polygenic and epistatic phenotypes, polyphonic-epistatic phenotypes have not been studied yet. Likely because the existing GWAS methods are under-powered to perform such compute-intensive association studies. VariantSpark is the first methodology to perform complex association analyses on whole-genome sequencing experiments and outperforms other state of the art implementations.

Results provided in this paper, firstly demonstrate the capability of VariantSpark in detecting associative signals of complex interactions, and secondly elaborate the performance and scalability when processing large-scale datasets. Akin to DeepLearning methods, VariantSpark's hyper-parameters need to be iteratively tuned to each dataset, which is made possible by VariantSpark's speed and scalability.

VariantSpark is not a replacement for traditional association analysis, but a compliment. The result of traditional GWAS (LR) and VariantSpark should be considered together to gain insights into the full influence of the genome on disease and other phenotypes.

Availability of source code

VariantSpark source code and compilation instructions are available on https://github.com/aehrc/VariantSpark. To facilitate the use of VariantSpark, we made it available on the AWS Marketplace (https://aws.amazon.com/marketplace/pp/AEHRC-VariantSpark-Notebook/B07YVND4TD) such that a user with minimal technical knowledge can get access to a computer cluster of any size with VariantSpark, Hail and Jupyter notebook installed and ready to use. We also provide AWS CloudFormation templates to create a similar environment as in AWS Marketplace (https://github.com/aehrc/VariantSpark-aws). Finally, VariantSpark has been registered on https://bio.tools/ (biotools:variantspark) and https://scicrunch.org/ (RRID: SCR_018383).

Availability of supporting data and materials

Supplementary Data File 1 provides an extended and more detailed numerical comparison for all figures. *Supplementary Data File 2* includes the maximum correlation values (γ) for all experiments. *Supplementary Data File 3* provide all PEPS simulated phenotypes for 1000-Genomes dataset with truth-variants and PEPS configuration files. *Supplementary Data File 4* explains access to raw data and output file (available on AWS S3) as well as technical instruction including

- 1000-Genome data and subsets in vcf compressed format.
- Dataset simulated with VariantSpark.
- Complete correlation matrix between TVs and RVs for all analyses.
- List of RVs for all analyses.
- Random-Forest model created by VariantSpark in JSON format (used to compute average tree depth and number of nodes)
- Instructions to create an AWS EMR cluster via terminal.
- Instructions to submit VariantSpark jobs to the cluster.

Declarations

List of abbreviations

- PRS: Polygenic Risk Score
- GWAS: Genome wide association study
- RF: Random-Forest
- HPC: High-Performance Computer
- DT: Decision-Tree
- 1KG: 1000-Genomes
- PEPS: Polygenic Epistatic Phenotype Simulator
- *OOB* : out-of-bag error rate
- TV: Truth-variants
- RV: Ranked-variants

List of definitions

- *nS*: Number of samples in dataset
- *nV*: Number of variants in dataset
- *nTree*: Number of trees
- *mTry*: Number of variable to evaluate at each node of a tree
- *maxD*: Maximum depth of a tree
- *minNS*: Minimum number of samples in each node to be processed
- *rbs*: number of tree to be processed in parallel.
- γ: The maximum absolute Pearson correlation coefficient between a truth-variant and any of the ranked-variants

Author's Contributions

AB and DCB designed the experiment. PS, AB, BH, YJ, CH, and ARO implemented the software used in this research. AB and RD conducted the experiments. AB, DCB, OJL, and NT wrote the document. All authors read and approved the manuscript.

Acknowledgments

The authors gratefully acknowledge AWS cloud credits that were used to fund the cloud cost of this work.

References

1. Visscher PM, Wray NR, Zhang Q, Sklar P, McCarthy MI, Brown MA, et al. 10 years of GWAS discovery: biology, function, and translation. The American Journal of Human Genetics 2017;101(1):5–22.
2. MacArthur J, Bowler E, Cerezo M, Gil L, Hall P, Hastings E, et al. The new NHGRI-EBI Catalog of published genome-wide association studies (GWAS Catalog). Nucleic acids research 2017;45(D1):D896–D901.
3. Manolio TA, Collins FS, Cox NJ, Goldstein DB, Hindorff LA, Hunter DJ, et al. Finding the missing heritability of complex diseases. Nature 2009;461(7265):747–753.
4. Boyle EA, Li YI, Pritchard JK. An Expanded View of Complex Traits: From Polygenic to Omnigenic. Cell 2017;169(7):1177–1186.
5. Nicod J, et al. Genome-wide association of multiple complex traits in outbred mice by ultra-low-coverage sequencing. Nature genetics 2016;48(8):912.
6. Yang J, et al. Genome partitioning of genetic variation

for complex traits using common SNPs. Nature Genetics 2011;43(6):519–525.
7. Manolio TA, et al. Finding the missing heritability of complex diseases. Nature 2009;461(7265):747–753.
8. Wray NR, Goddard ME, Visscher PM. Prediction of individual genetic risk to disease from genome-wide association studies. Genome research 2007;17(10):1520–1528.
9. Chatterjee N, Shi J, García-Closas M. Developing and evaluating polygenic risk prediction models for stratified disease prevention. Nature Reviews Genetics 2016;17(7):392.
10. Mavaddat N, Pharoah PD, Michailidou K, Tyrer J, Brook MN, Bolla MK, et al. Prediction of breast cancer risk based on profiling with common genetic variants. JNCI: Journal of the National Cancer Institute 2015;107(5).
11. Phillips PC. Epistasis—the essential role of gene interactions in the structure and evolution of genetic systems. Nature Reviews Genetics 2008;9(11):855–867.
12. Niel C, Sinoquet C, Dina C, Rocheleau G. A survey about methods dedicated to epistasis detection. Frontiers in genetics 2015;6:285.
13. Shang J, Zhang J, Sun Y, Liu D, Ye D, Yin Y. Performance analysis of novel methods for detecting epistasis. BMC bioinformatics 2011 dec;12:475.
14. Wan X, Yang C, Yang Q, Xue H, Fan X, Tang NL, et al. BOOST: A fast approach to detecting gene-gene interactions in genome-wide case-control studies. The American Journal of Human Genetics 2010;87(3):325–340.
15. Evans DM, Spencer CC, Pointon JJ, Su Z, Harvey D, Kochan G, et al. Interaction between ERAP1 and HLA-B27 in ankylosing spondylitis implicates peptide handling in the mechanism for HLA-B27 in disease susceptibility. Nature genetics 2011;43(8):761–767.
16. Breiman L. Random Forests. Machine Learning 2001;45(1):5–32.
17. Qi Y. Random forest for bioinformatics. In: Ensemble machine learning Springer; 2012.p. 307–323.
18. Chen X, Ishwaran H. Random forests for genomic data analysis. Genomics 2012;99(6):323–329.
19. Goldstein BA, Polley EC, Briggs FB. Random forests for genetic association studies. Statistical Applications in Genetics and Molecular Biology 2011;10(1):32.
20. O'Brien AR, Saunders NFW, Guo Y, Buske FA, Scott RJ, Bauer DC. VariantSpark: population scale clustering of genotype information. BMC Genomics 2015;16(1).
21. Eraslan G, Avsec Ž, Gagneur J, Theis FJ. Deep learning: new computational modelling techniques for genomics. Nature Reviews Genetics 2019;20(7):389–403.
22. Zaharia M, Xin RS, Wendell P, Das T, Armbrust M, Dave A, et al. Apache spark: a unified engine for big data processing. Communications of the ACM 2016;59(11):56–65.
23. Massie M, Nothaft F, Hartl C, Kozanitis C, Schumacher A, Joseph AD, et al. ADAM: Genomics Formats and Processing Patterns for Cloud Scale Computing. UCB/EECS-2013-207, EECS Department, University of California, Berkeley; 2013.
24. Stephens ZD, Lee SY, Faghri F, Campbell RH, Zhai C, Efron MJ, et al. Big Data: Astronomical or Genomical? PLoS Biol 2015;13(7):e1002195.
27. Liaw A, randomForest package for R; 2020.
25. Team H, Hail Library. GitHub; 2020. https://github.com/hail-is/hail.
26. Wright MN, Ziegler A. Ranger: A Fast Implementation of Random Forests for High Dimensional Data in C++ and R. Journal of Statistical Software 2016;.
28. Ishwaran H, Kogalur UB, Blackstone EH, Lauer MS. Random survival forests. Ann Appl Statist 2008;2(3):841–860.
29. Schwarz DF, König IR, Ziegler A. On safari to Random Jungle: a fast implementation of Random Forests for high-dimensional data. Bioinformatics 2010;26(14):1752–1758.
30. Telenti A, other. Deep sequencing of 10,000 human genomes. Proceedings of the National Academy of Sciences 2016;113(42):11901–11906.
31. Bayardo BP, Herbach JS, Basu S, J R. PLANET: Massively Parallel Learning of Tree Ensembles with MapReduce. Proceedings of the 35th International Conference on Very Large Data Bases (VLDB-2009) 2009;.
32. Meng X, Bradley J, Yavuz B, Sparks E, Venkataraman S, Liu D, et al. MLlib: Machine learning in apache spark. The Journal of Machine Learning Research 2016;17(1):1235–1241.
33. Bayat A, Szul P, O'Brien AR, Dunne R, Luo OJ, Jain Y, et al. VariantSpark, A Random Forest Machine Learning Implementation for Ultra High Dimensional Data. bioRxiv 2019;p. 702902.
34. Lulli A, Oneto L, Anguita D. Reforest: random forests in apache spark. In: International conference on artificial neural networks Springer; 2017. p. 331–339.
35. Pafka S, benchm-ml GitHub Page. GitHub; 2020. https://github.com/szilard/benchm-ml.
36. Chen T, Guestrin C. Xgboost: A scalable tree boosting system. In: Proceedings of the 22nd acm sigkdd international conference on knowledge discovery and data mining ACM; 2016. p. 785–794.
37. H2O, H2O, editor, Open-source machine learning platform for enterprises. web; 2018. https://www.h2o.ai/h2o/.
38. Oneto L, Cipollini F, Lulli A, Anguita D, ReForeSt GitHub Page. GitHub; 2020. https://github.com/alessandrolulli/reforest.
39. Chen J, Li K, Tang Z, Bilal K, Yu S, Weng C, et al. A parallel random forest algorithm for big data in a spark cloud computing environment. IEEE Transactions on Parallel and Distributed Systems 2016;28(4):919–933.
40. Abuzaid F, Bradley JK, Liang FT, Feng A, Yang L, Zaharia M, et al. Yggdrasil: An Optimized System for Training Deep Decision Trees at Scale. Advances in Neural Information Processing Systems 2016;29:3817–3825.
41. Leo Breiman CJSRAO Jerome Friedman. Classification and Regression Trees. 1 ed. Belmont, California, U.S.A.: Wadsworth Publishing Company; 1984.
42. Kleinbaum DG, Dietz K, Gail M, Klein M, Klein M. Logistic regression. Springer; 2002.
43. Consortium GP, et al. A global reference for human genetic variation. Nature 2015;526(7571):68.
44. Bayat A, Hosking B, PEPS: Polygenic Epistatic Phenotype Simulator. GitHub; 2020. https://github.com/aehrc/PEPS.

Click here to access/download
Supplementary Material
LetterToEditorAndResponseToReviewers.pdf

Click here to access/download
Supplementary Material
SupplementaryDataFile3(PEPS-Phenotype).rar

SupplementaryDataFile2

Click here to access/download
Supplementary Material
SupplementaryDataFile2(Maximum-Correlation).rar

SupplementaryDataFile4

Click here to access/download
Supplementary Material
SupplementaryDataFile4(AccessData).pdf

Click here to access/download
Supplementary Material
SupplementaryDataFile1(NumericalResults).xls
