# Supplementary material for: VariantSpark: Cloud-based machine learning for association study of complex phenotype and large-scale genomic data
Source: Gigascience. 2020 Aug 6;9(8):giaa077. doi: 10.1093/gigascience/giaa077 (PMC7407261; doi:10.1093/gigascience/giaa077)
Supplement: giaa077_Supplemental_Files [file giaa077_supplemental_files.zip › SupplementaryDataFile4(AccessData).pdf]

All raw data and results are publicly available through **GigaDB**.

http://gigadb.org/dataset/100759

Link to FTP Site:

ftp://parrot.genomics.cn/gigadb/pub/10.5524/100001_101000/100759/

There are two folders (folder structure is only available on FTP site)

- **Dataset**: Contains the raw data processed by each algorithm
- **Results**: The raw output files which are used to prepare the result for the paper.

Dataset

The **Dataset** folder has the following subfolders which we describe them below

- **1KG-Subset**: 1000-Genome dataset and subsets in *vcf.bgz* format
- **PEPS-Phenotypes**: Information related to the simulation of PEPS phenotype.
- **VS-GT:** Genotype dataset simulated by VariantSpark simulator
- **VS-Pheno:** Phenotypes simulated by VariantSpark simulator

1KG-Subset

There are 5 *vcf.bgz* files in this folder each for a subset. Note that the naming convention we used in the paper is different from the naming convention we use in the experiment (see table below). Note that we break multiallelic variants to biallelic variants which slightly increases the number of variants. That is the only difference with the original 1000-Genome dataset.

Paper Name	Experiment Name
1KG-80M	Subset.0
1KG-5M	Subset.2
1KG-500K	Subset.3
1KG-5M-T	Subset.2.withTruth
1KG-500K-T	Subset.3.withTruth

PEPS-Phenotypes

For each phenotype we use in the experiment we share the PEPS output config file **(*.config.json)**, truth-variants **(*.TruthSNP.csv)**, and the binary phenotype **(*.pheno.csv)**. We initially subset ~20,000 variants from 1000-Genome data, convert them to a CSV format and then compress and store them in **PEPS-1000Genome-Subset-A.csv.gz**. As you can see in the phenotype config file all phenotypes are simulated based on a random selection of SNPs from this subset. Also, when we create the 1KG-5M-T and 1KG-500K-T subsets above, we push all SNPs in **PEPS-1000Genome-Subset-A.csv.gz** to 1KG-5M and 1KG-500K subsets respectively. Thus 1KG-5M-T and 1KG-500K-T includes all truth-variants for all phenotype. Note that we use a different naming convention for the phenotypes in the experiment which is described below. Also, in our naming **run1**, **run2** and **run3** represent three replicates of the phenotype simulation.

Paper Name	Experiment Name
PIL	Cnf01
PIM	Cnf02
PIH	Cnf03
PEL	Cnf04
PEM	Cnf05
PEH	Cnf06
PXL	Cnf07
PXM	Cnf08
PXH	Cnf09

VS-GT

Genotype dataset simulated by VariantSpark (we convert the parquet file into CSV and compress them with bzip2). Sample names are **S0, S1, …, Sn** and variant name are **v_0, v_1, …, v_m.** There are 2 numbers in the file name of each dataset where the first one is the number of samples and the second one is the number of variants. Those files with "s" and "v" prefix on the number of samples and variants are smaller subsets of 10K-10M dataset where all five truth-variants are included. This the same phenotype used for 10K-10M can be used for all of them. Note that the following files are identical and both represent 10K-10M dataset

- Dataset.10000.10000000.csv.bz2
- Dataset.s10000.v10000000.csv.bz2

In the CSV files genotype are encoded to 0, 1 and 2 (for 0/0 and 0/1 and 1/1)
CSV files are similar to VCF file where rows are variants and columns are samples.

VS-Pheno

Phenotype files use the same naming convention as genotype files above.
Each phenotype file **(*.pheno.csv)** includes 8 columns in this order:

- Sample id
- Binary phenotype
- Continues phenotype
- The genotype of 5 truth variants encoded to 0, 1 and 2 (for 0/0 and 0/1 and 1/1)

Results

There are the following subfolders in the **Result** folder

- **CorrMatrix**: Correlation coefficient matrix between truth-variant (columns) and top 10t ranked-variants (rows) by VariantSpark (vs) and Logistic-Regression (lr) for all phenotype and subsets (t is the number of truth-variants). The file name contains the GWAS method (vs/lr), subset, phenotype and replicate number.
- **RankedVariants**: the list of top 10t ranked-variants (sorted by rank so that the head of the file contains the most associative variants). The file name contains the GWAS method (vs/lr), subset, phenotype and replicate number.
- **RF-JSON**: Random-Forest models trained by VariantSpark (in json format) to compute the tree statistics in *Supplementary Data File 1* (sheet: *VariantSpark Runtime*)

Experimental Setup

To create an AWS cluster with VariantSpark installed we recommend using AWS marketplace (https://aws.amazon.com/marketplace/pp/AEHRC-VariantSpark-Notebook/B07YVND4TD) which is free (VariantSpark is free but the user should pay for AWS resources anyway). Alternatively, you can use the following script which uses aws-cli2 program. You may change the instance count to have cluster of different size.

```
ClusterName="C256" #options: C32, C64, C128, C256, C512, C1024
InstanceCount="16" #options: 2  , 4  , 8   , 16  , 32  , 64 # Each instance count as 16 CPU

LogURI=s3n://your-s3-path

# Do not change below lines

JSON_FMT='[{"InstanceCount":%s,"BidPrice":"OnDemandPrice","EbsConfiguration":{"EbsBlockDeviceConfigs":[{"VolumeSpecification":{"SizeInGB":64,"VolumeType":"gp2"},"VolumesPerInstance":4}]},"InstanceGroupType":"CORE","InstanceType":"r4.4xlarge","Name":"Core"},{"InstanceCount":1,"BidPrice":"OnDemandPrice","EbsConfiguration":{"EbsBlockDeviceConfigs":[{"VolumeSpecification":{"SizeInGB":32,"VolumeType":"gp2"},"VolumesPerInstance":4}]},"InstanceGroupType":"MASTER","InstanceType":"r4.2xlarge","Name":"Master"}]'

InstanceGroups=$(printf "$JSON_FMT" "$InstanceCount")

## only works with aws2. old aws command has some issue parsing the bidPrice
aws2 emr create-cluster --name "$ClusterName" --log-uri "$LogURI" --applications Name=Spark Name=Ganglia --ec2-attributes '{"InstanceProfile":"EMR_EC2_DefaultRole"}' --release-label emr-5.27.0 --instance-groups $InstanceGroups --configurations '[{"Classification":"spark-defaults","Properties":{"spark.hadoop.io.compression.codecs":"org.apache.hadoop.io.compress.DefaultCodec,is.hail.io.compress.BGzipCodec,org.apache.hadoop.io.compress.GzipCodec","spark.executor.extraClassPath":"/usr/lib/hadoop-lzo/lib/*:/usr/lib/hadoop/hadoop-aws.jar:/usr/share/aws/aws-java-sdk/*:/usr/share/aws/emr/emrfs/conf:/usr/share/aws/emr/emrfs/lib/*:/usr/share/aws/emr/emrfs/auxlib/*:/usr/share/aws/emr/security/conf:/usr/share/aws/emr/security/lib/*:./hail-all-spark.jar","spark.kryo.registrator":"is.hail.kryo.HailKryoRegistrator","spark.driver.extraClassPath":"/usr/lib/hadoop-lzo/lib/*:/usr/lib/hadoop/hadoop-aws.jar:/usr/share/aws/aws-java-sdk/*:/usr/share/aws/emr/emrfs/conf:/usr/share/aws/emr/emrfs/lib/*:/usr/share/aws/emr/emrfs/auxlib/*:/usr/share/aws/emr/security/conf:/usr/share/aws/emr/security/lib/*:/home/hadoop/hail-all-spark.jar","spark.serializer":"org.apache.spark.serializer.KryoSerializer","spark.dynamicAllocation.enabled":"false","spark.jars":"/home/hadoop/hail-all-spark.jar,/home/hadoop/variant-spark-all.jar"}},{"Classification":"spark-env","Properties":{},"Configurations":[{"Classification":"export","Properties":{"PYSPARK_PYTHON":"/usr/bin/python3","PYSPARK_DRIVER_PYTHON":"${PYSPARK_DRIVER_PYTHON:-/home/hadoop/biospark/bin/python}","PYSPARK_DRIVER_PYTHON_OPTS":"${PYSPARK_DRIVER_PYTHON_OPTS:-}"}}]},{"Classification":"spark","Properties":{"maximizeResourceAllocation":"true"}}]' --auto-scaling-role EMR_AutoScaling_DefaultRole --bootstrap-actions '[{"Path":"s3://variant-spark/GigaScience/biospark/1.0.1/bootstrap/install-biospark.sh","Args":["--biospark-url","s3://variant-spark/GigaScience/biospark/1.0.1"],"Name":"Install Biospark"}]' --ebs-root-volume-size 32 --service-role EMR_DefaultRole --enable-debugging --scale-down-behavior TERMINATE_AT_TASK_COMPLETION
```

To submit a VariantSpark job to the cluster you can use below script which uses aws-cli2. You should change the given parameters in the script.

```
Replace "j-XXXXXXXXXXXXX" with cluster id.

####### For csv genotype file

aws2 emr add-steps --cluster-id j- XXXXXXXXXXXXX --steps '[{"Args":["spark-submit","--
deploy-mode","client","--
class","au.csiro.variantspark.cli.VariantSparkApp","/home/hadoop/biospark/lib/python3.6/sit
e-packages/varspark/jars/variant-spark_2.11-0.3.0-SNAPSHOT-all.jar","importance","-
io","{\"defVariableType\":\"ORDINAL(3)\"}","-sp","16","-sr","13","-v","-ro","-rn","100","-
rbs","10","-rmtf","0.1","-it","csv","-if","s3://path-to/data.csv.bz2","-fc","label","-
ff","s3://path-to/data.pheno.csv","-of","s3://path-to/output.vsis.csv","-on","1000000","-
om","s3://path-to/output.rf.json","-omf","json","-rmns","50","-
rmd","15"],"Type":"CUSTOM_JAR","ActionOnFailure":"CONTINUE","Jar":"command-
runner.jar","Properties":"","Name":"Test"}]'

####### For VCF genotype file

aws2 emr add-steps --cluster-id j- XXXXXXXXXXXXX --steps '[{"Args":["spark-submit","--
deploy-mode","client","--
class","au.csiro.variantspark.cli.VariantSparkApp","/home/hadoop/biospark/lib/python3.6/sit
e-packages/varspark/jars/variant-spark_2.11-0.3.0-SNAPSHOT-all.jar","importance","-
sp","16","-sr","13","-v","-ro","-rn","100","-rbs","10","-rmtf","0.1","-it","vcf","-
if","s3://path-to/data.vcf.bgz","-fc","label","-ff","s3://path-to/data.pheno.csv","-
of","s3://path-to/output.vsis.csv","-on","1000000","-om","s3://path-to/output.rf.json","-
omf","json","-rmns","50","-
rmd","15"],"Type":"CUSTOM_JAR","ActionOnFailure":"CONTINUE","Jar":"command-
runner.jar","Properties":"","Name":"Test"}]'
```

VariantSpark parameters are:

-if: S3 path to genotype file

-ff: S3 path to phenotype file

-fc: Name of phenotype column in phenotype file

-of: S3 path to output importance-score file

-om: S3 path to output RF-model file

-rn: Number of trees (nTree)

-rbs: Batch size (number of trees to process in parallel)

-rmtf: Fraction of number of variants to be used as mtry

-rmt: mtry (not to be used with rmtf)

-rmns: Minimum node size (minNS)

-rmd: Maximum Tree depth (maxD)

-sp: Spark parallelisation (twice the number of CPUs in cluster)

-sr: Random seed

-ro: compute OOB
