# Supplementary material for: VariantSpark: Cloud-based machine learning for association study of complex phenotype and large-scale genomic data
Source: Gigascience. 2020 Aug 6;9(8):giaa077. doi: 10.1093/gigascience/giaa077 (PMC7407261; doi:10.1093/gigascience/giaa077)
Supplement: giaa077_Response_to_Reviewer_Comments_Original_Submission [file giaa077_response_to_reviewer_comments_original_submission.pdf]

Author's Response To Reviewer Comments

Close

Letter to the editor and response to reviewers are attached as a supplementary data for reviewers only (LetterToEditorAndResponseToReviewers.pdf)
Proof of compile is provided as supplementary data for reviewers only (ProofOfCompile.pdf)

Close
