# Supplementary material for: VariantSpark: Cloud-based machine learning for association study of complex phenotype and large-scale genomic data
Source: Gigascience. 2020 Aug 6;9(8):giaa077. doi: 10.1093/gigascience/giaa077 (PMC7407261; doi:10.1093/gigascience/giaa077)
Supplement: giaa077_Response_to_Reviewer_Comments_Revision_1 [file giaa077_response_to_reviewer_comments_revision_1.pdf]

Author's Response To Reviewer Comments

Close

Letter to editor and response to reviewers are attached as personal cover (single PDF file)
Proof of compile is attached as Supplementary File (PDF file)

Close
