# Supplementary material for: VariantSpark: Cloud-based machine learning for association study of complex phenotype and large-scale genomic data
Source: Gigascience. 2020 Aug 6;9(8):giaa077. doi: 10.1093/gigascience/giaa077 (PMC7407261; doi:10.1093/gigascience/giaa077)
Supplement: giaa077_Response_to_Reviewer_Comments_Revision_2 [file giaa077_response_to_reviewer_comments_revision_2.pdf]

# Author's Response To Reviewer Comments

Close

Dear Editor,

We have updated our manuscript and include the GigaDB reference to the manuscript. The GigaDB reference has been cited in two different places of the manuscript (Dataset section and Supporting data section). We also update the supplementary data file 4 to reflect GigaDB doi url. We have included a proof of compile pdf file in our submission. You can compile our project on Overleaf using the following link:
https://www.overleaf.com/read/kyvgsgvhwjyv

Yours Faithfully,
Authors

Close
