# Supplementary material for: VariantSpark: Cloud-based machine learning for association study of complex phenotype and large-scale genomic data
Source: Gigascience. 2020 Aug 6;9(8):giaa077. doi: 10.1093/gigascience/giaa077 (PMC7407261; doi:10.1093/gigascience/giaa077)
Supplement: giaa077_Response_to_Reviewer_Comments_Revision_3 [file giaa077_response_to_reviewer_comments_revision_3.pdf]

Author's Response To Reviewer Comments

Close

Dear Editor,

We perform the following changes in this submission:
- Format "Availability of Source Code" section as requested.
- Add GigaDB citation in 3 places
1: In the "Availability of source code" section with the statement you mentioned.
2: In the "Availability of the supporting data"
3: In the "Dataset" section
- Add funding statement. Also add the funding in the submission form as it appears on GigaDB website (Please revise it if necessary)
- Remove acknowledgment statement (AWS credit now goes into funding statement)
- Minor changes to Supplementary data file 4 (add a link to FTP site)

Proof of Compile is also provided as supplementary data (for review only)

Yours Sincerely,
Authors

Close
