# Supplementary material for: VariantSpark: Cloud-based machine learning for association study of complex phenotype and large-scale genomic data
Source: Gigascience. 2020 Aug 6;9(8):giaa077. doi: 10.1093/gigascience/giaa077 (PMC7407261; doi:10.1093/gigascience/giaa077)
Supplement: giaa077_Reviewer_1_Report_Original_Submission — Brendan Lawlor -- 10/13/2019 Reviewed [file giaa077_reviewer_1_report_original_submission.pdf]

Reviewer Report

Title: VariantSpark: Cloud-Based Machine Learning for Association Study of Complex Phenotype and Large-Scale Genomic Data

Version: Original Submission **Date:** 10/13/2019

Reviewer name: Brendan Lawlor

Reviewer Comments to Author:

1. The paper describes the use of a program on Apache Spark to create decision trees in a random forest applied to data of particularly high dimensionality - in this case variant data from the 1000 Genomes project.
The program, called VariantSpark, is proposed as more performant and more accurate than a list of alternatives.
The authors hold that this program exhibits novelty in the way parallelization for random forests is implemented, and for the number of features that it can process.
The experiment has 4 sections:
a. A comparison of run time and accuracy of competing random forest implementations on increasing subsets of a common dataset;
b. An exploration of the VariantSpark program's scalability using a synthetic dataset
c. An exploration of the effects of choice of mtry and ntrees parameters on the accuracy of VariantSpark
d. A direct comparison of performance between VariantSpark and yggdrasil, with the former set to create a single Decision Tree in order to approximate the behavior of the latter.

2. There are a number of typos or grammatical errors that should be fixed to aid readability, as follows:
a. Page 1. paragraph 2: "GWAS context" - > "a GWAS context" or "GWAS contexts"
b. Page 4, paragraph 5: Various issues in text. To be re-proofed.
c. Page 6, paragraph 2: "outpacing" -> "outpace"
3. Page 2. Paragraph 1 states "all of these implementations are optimized ... for HPC". But H20.ai is listed in Table 1 as running on a proprietary cluster.
4. Page 2, paragraph 7 states "execution time of all methods" should specify "all Random Forest methods" so as not to include yggdrasil. Also, please state here which cluster was used.
5. Page 2, paragraph 7 : A 500K dataset is mentioned in the narrative, but not in the associated table 2. If the samples and features columns for table 2 were provided as they are with table 3, it would make fig 1a clearer with respect to which data points were which.
6. General point: Given the title of the paper and the name of the program, and the fact that the y-axis of fig 1b uses it, some treatment of the word "variant" would aid some readers, perhaps with reference to the 1000 Genome VCF files that serve as input.
7. Page 3, paragraph 1: The maximum values of p (50,000,000) and samples (10,000) do not correspond to the values in Table 3 (81M and 1.5K respectively)
8. Page 3, paragraph 2: Fig 2a does not represent the narrative, or support the conclusions, of this

paragraph. The narrative describes an experiment where mtry is held fixed, 100 trees are built, while p and n are increased and the time to build the trees is measured.
Fig 2a shows the number of cores on the x-axis and the trees-per-hour on the y-axis, as well as showing 4 mtry scenarios. The caption corresponds to the narrative, and not the plot.
There is no breakdown of n and p to demonstrate the (respectively) sub-linear and linear scaling described in the narrative.
Figure 2b does not appear to support the "linear scalability" of this sections title, with respect to CPU, or even the "scales well" of the narrative.
The four scenarios of the "ncore", where the number of cores is doubled from 16, to 32, to 64, to 128, shows rapidly diminishing returns in terms of speedup for 100 trees, at all values of mtry.
If an open-ended approach is taken, as with Fig 1a where a 'trees-per-hour" metric is used, then we can say that things look better, but to use the authors' own language, it is sub-linear.
There is no explanation of how the number of cores is controlled. Given the values used, it appears that increasing numbers of worker nodes are used (1,2, 4 and 8, but never all 12?). Or was it controlled by setting the number of executors?
In summary, there is a disconnect between the narrative and its claims, when compared to the data presented in the figures. The authors should reconsider what claims they wish to make, and re-build a suitable narrative, with directly corresponding data/figures.
9. Page 4, Paragraph 5: The description of the implementation is poor. It has typo/grammatical errors that confuse the reader, and terms are not clearly defined. What follows is a set of questions that is not exhaustive but indicates points of confusion.
a. Is 'worker' intended to mean 'worker node' in the Spark sense?
b. Does each 'i' partition build its own tree?
c. If not, what decides how many trees are to be built overall, and per worker/node?
d. Does the word "node" in the phrase "best split locally for the node" refer to worker node (Spark) or tree node (Decision Tree)?
e. "Assume one [executor] per worker": If "worker" means "worker node", this does not correspond to the number of executors in table 4.
Given the above, this paragraph could do with re-writing for clarity. Explaining complex code in concise English is challenging, but necessary. The burden on the authors would be lightened if the scripts that ran all 4 sections of the experiment were made available as part of the source code in github.
Summary:
Description of methods could be more systematic. Several readings were required before a clear (albeit still incomplete) picture of the experimental design emerged.
This would be offset if there were a way of reproducing the 4 phases of the experiment. While I was able to build Variant Spark and run the built-in examples, there were no instructions for reproducing the experiments themselves.
Given the cloud-based nature of the experiment, it would have been possible for the authors to provide Terraform/CloudFormation scripts to reproduce the clusters, and scripts to run the experiment on those clusters. This would have lightened the burden of describing the experiment and presenting its novel aspects.
The idea to use Apache Spark to create random forests for high-dimensional data is a good one, and

clearly a great deal of work has gone into the implementation.
But while the experimental design seems sensible, the paper does not present the experimental data in a way that supports all of the paper's conclusions.

Level of Interest

Please indicate how interesting you found the manuscript: Choose an item.

Quality of Written English

Please indicate the quality of language in the manuscript: Choose an item.

Declaration of Competing Interests

Please complete a declaration of competing interests, considering the following questions:

- Have you in the past five years received reimbursements, fees, funding, or salary from an organisation that may in any way gain or lose financially from the publication of this manuscript, either now or in the future?
- Do you hold any stocks or shares in an organisation that may in any way gain or lose financially from the publication of this manuscript, either now or in the future?
- Do you hold or are you currently applying for any patents relating to the content of the manuscript?
- Have you received reimbursements, fees, funding, or salary from an organization that holds or has applied for patents relating to the content of the manuscript?
- Do you have any other financial competing interests?
- Do you have any non-financial competing interests in relation to this paper?

If you can answer no to all of the above, write 'I declare that I have no competing interests' below. If your reply is yes to any, please give details below.

I declare that I have no competing interests.

I agree to the open peer review policy of the journal. I understand that my name will be included on my report to the authors and, if the manuscript is accepted for publication, my named report including any attachments I upload will be posted on the website along with the authors' responses. I agree for my report to be made available under an Open Access Creative Commons CC-BY license (http://creativecommons.org/licenses/by/4.0/). I understand that any comments which I do not wish to be included in my named report can be included as confidential comments to the editors, which will not be published.

Choose an item.

To further support our reviewers, we have joined with Publons, where you can gain additional credit to further highlight your hard work (see: https://publons.com/journal/530/gigascience). On publication of this paper, your review will be automatically added to Publons, you can then choose whether or not to claim your Publons credit. I understand this statement.

Yes Choose an item.
