# Supplementary material for: VariantSpark: Cloud-based machine learning for association study of complex phenotype and large-scale genomic data
Source: Gigascience. 2020 Aug 6;9(8):giaa077. doi: 10.1093/gigascience/giaa077 (PMC7407261; doi:10.1093/gigascience/giaa077)
Supplement: giaa077_Reviewer_1_Report_Revision_1 — Brendan Lawlor -- 5/19/2020 Reviewed [file giaa077_reviewer_1_report_revision_1.pdf]

Reviewer Report

Title: VariantSpark: Cloud-Based Machine Learning for Association Study of Complex Phenotype and Large-Scale Genomic Data

Version: Revision 1 **Date:** 5/19/2020

Reviewer name: Brendan Lawlor

Reviewer Comments to Author:

This paper has clearly gone through a major rewrite since the last review. The narrative is clearer and issues of reproducibility on the cloud have been addressed.
There remain a few minor points around readability. I present a non-exhaustive list below, and generally recommend that parts of the paper be proof-read for basic grammatical errors.
Some of the diagrams required multiple passes in order to appreciate the information they were conveying.
Overall I'm happy to recommend this paper for publication. The narrative focuses on the particular use case of high-dimension GWAS studies, and goes to the trouble of explaining current relevant issues around phenotypes influenced by gene interaction and epistasis. This has the benefit of educating readers of a more informatic background, while at the same time giving biologists the raison d'etre for the technology: the key facts that "variant interactions remain invisible to traditional GWAS" while algorithms that address these interactions typically do not scale to whole-genome data. The space devoted to this in the paper is particularly welcome in a cross-disciplinary field where two audiences need to be addressed simultaneously.
I was able to build the software provided on its master branch (with some adjustments like disabling unit tests and switching off the scala formatting plugin). Creating the overall infrastructure was also straightforward, using the AWS marketplace option with the provided AWS Cloudformation Templates. Note that the steps presented in the explanatory video (another very welcome addition) are slightly out-of-date with respect to those that need to be followed today, but not enough to be of serious concern. In principle, using this approach, it would have been possible for me to run large datasets like those used for the experiment (also provided). As it stood, I was able to verify the correct functioning of the running system.
All major cloud providers have analogous products, and there are some products such as Terraform which work across multiple cloud providers. Papers such as this one do well in making use of them.
Non-exhaustive list of issues relating to grammar, spelling or readability follows. In general, despite a very well written initial section, some very basic errors were made from page 3 (Datasets) onwards. I don't propose to list everything here, but strongly recommend that it be proof-read.
p 3 para 8: "We generate four subsets [of/from?] this data"
Table 2 caption: "... these dataset[s].
Table 4 caption: "All dataset includes" -> "All datasets include"
Table 4 data: There appear to be duplicate rows for 256X and 513X
p4 para 5: "five genotype dataset[s]"

p4 para 6: "In the last two replicate[s]"
p4 para 7 : "We build a forest with 10 tree[s]
p4 para 8: "applied to all experiment[s]"
Figure 1b caption: on last line (beginning "Where, ..." move the comma to after variants, and change 'is' to 'are' for variants.
p6 para 5: "highly correlates [with?] the TV and possibly [identifies]
p6 para 6 "VariantSaprk" to "VariantSpark"
p6 para 6 "of exclusively detect[ed] variants"
Figure 3 caption: "...black line [illustrates] if the..."
p10 para 2: "polyphonic" -> "polygenic"?
List of definitions: "mTry: Number of variable[s].."

Level of Interest

Please indicate how interesting you found the manuscript: Choose an item.

Quality of Written English

Please indicate the quality of language in the manuscript: Choose an item.

Declaration of Competing Interests

Please complete a declaration of competing interests, considering the following questions:

- Have you in the past five years received reimbursements, fees, funding, or salary from an organisation that may in any way gain or lose financially from the publication of this manuscript, either now or in the future?
- Do you hold any stocks or shares in an organisation that may in any way gain or lose financially from the publication of this manuscript, either now or in the future?
- Do you hold or are you currently applying for any patents relating to the content of the manuscript?
- Have you received reimbursements, fees, funding, or salary from an organization that holds or has applied for patents relating to the content of the manuscript?
- Do you have any other financial competing interests?
- Do you have any non-financial competing interests in relation to this paper?

If you can answer no to all of the above, write 'I declare that I have no competing interests' below. If your reply is yes to any, please give details below.

I declare that I have no competing interests

I agree to the open peer review policy of the journal. I understand that my name will be included on my report to the authors and, if the manuscript is accepted for publication, my named report including any attachments I upload will be posted on the website along with the authors' responses. I agree for my report to be made available under an Open Access Creative Commons CC-BY license (http://creativecommons.org/licenses/by/4.0/). I understand that any comments which I do not wish to be included in my named report can be included as confidential comments to the editors, which will not be published.

Choose an item.

To further support our reviewers, we have joined with Publons, where you can gain additional credit to further highlight your hard work (see: https://publons.com/journal/530/gigascience). On publication of this paper, your review will be automatically added to Publons, you can then choose whether or not to claim your Publons credit. I understand this statement.

Yes Choose an item.
