# Supplementary material for: VariantSpark: Cloud-based machine learning for association study of complex phenotype and large-scale genomic data
Source: Gigascience. 2020 Aug 6;9(8):giaa077. doi: 10.1093/gigascience/giaa077 (PMC7407261; doi:10.1093/gigascience/giaa077)
Supplement: giaa077_Reviewer_2_Report_Original_Submission — Joshua W. K. Ho, PhD -- 10/18/2019 Reviewed [file giaa077_reviewer_2_report_original_submission.pdf]

Reviewer Report

Title: VariantSpark: Cloud-Based Machine Learning for Association Study of Complex Phenotype and Large-Scale Genomic Data

Version: Original Submission **Date:** 10/18/2019

Reviewer name: Joshua W. K. Ho, PhD

Reviewer Comments to Author:

Bayat et al developed a scalable distributed random forest machine learning algorithm, and provided an implementation of this algorithm in their previously developed software called VariantSpark. The major innovation and significance is their algorithm's ability to efficiency deal with ultra high dimensional data. Using real genomic data and synthetic data, they have shown that their implementation has excellent scalability property, and represent a significant improvement in terms of run-time performance compared to other cloud-based software. I believe this distributed implementation of random forest is indeed addressing an important unmet need in genomic big data analysis.

I have some concerns regarding the scope of work and readability of the manuscript:

1. Scope of work: In the abstract and introduction of the manuscript, the authors mentioned IoT and life sciences as the broad application domain that may benefit from your tool. While I understand the general argument, I must note that the authors have only shown that VariantSpark works well with human genome variant data - which are 'wide' and 'big' data in which the features are homogenous and of the same scale. IoT data, for example, not only have high dimensions, but often involve heterogeneous types of features that may have units in different scales (e.g., a single wearable smartwatch can generate time course for step count, GPS, heart rate, temperature and ECG data, all at the same time). It is unclear that their random forest approach will work well in this type of situations. I suggest the authors either focus on the genomic applications of VariantSpark (and highlight this fact in the title of the paper), or perform additional experiments with a variety of data to support their claim that VariantSpark is useful for all high dimensional data.
2. Methods: For a technical note, I expect that the description of the method and the software is front and centre of the manuscript. Currently, such information can only be found towards the end of the manuscript. I almost think the distributed random forest algorithm and the VariantSpark implementation should be a sub-section within the Results section. The data set and evaluation details can be kept in the 'Methods' section. I suggest the authors should consider adding a figure to show a schematic of their implementation of the distributed random forest algorithm.
3. Application: A central assumption behind this work is that building a machine learning classifier with ultra high dimensional data is useful. In practice, most people will do some sorts of feature selection prior to training a classifier. In this case, the dimensions are generally not too high. Therefore, the needs for a distributed algorithm is not necessarily that high. Besides using 1000 Genome data to separate individuals based on ethnicity - which is not a difficult task at all, I wonder if the authors can provide a more realistic genomic example in which the classification accuracy depends heavily on having

a large number of features.

Level of Interest

Please indicate how interesting you found the manuscript: Choose an item.

Quality of Written English

Please indicate the quality of language in the manuscript: Choose an item.

Declaration of Competing Interests

Please complete a declaration of competing interests, considering the following questions:

- Have you in the past five years received reimbursements, fees, funding, or salary from an organisation that may in any way gain or lose financially from the publication of this manuscript, either now or in the future?
- Do you hold any stocks or shares in an organisation that may in any way gain or lose financially from the publication of this manuscript, either now or in the future?
- Do you hold or are you currently applying for any patents relating to the content of the manuscript?
- Have you received reimbursements, fees, funding, or salary from an organization that holds or has applied for patents relating to the content of the manuscript?
- Do you have any other financial competing interests?
- Do you have any non-financial competing interests in relation to this paper?

If you can answer no to all of the above, write 'I declare that I have no competing interests' below. If your reply is yes to any, please give details below.

I declare that I have no competing interests

I agree to the open peer review policy of the journal. I understand that my name will be included on my report to the authors and, if the manuscript is accepted for publication, my named report including any attachments I upload will be posted on the website along with the authors' responses. I agree for my report to be made available under an Open Access Creative Commons CC-BY license (http://creativecommons.org/licenses/by/4.0/). I understand that any comments which I do not wish to be included in my named report can be included as confidential comments to the editors, which will not be published.

Choose an item.

To further support our reviewers, we have joined with Publons, where you can gain additional credit to further highlight your hard work (see: https://publons.com/journal/530/gigascience). On publication of this paper, your review will be automatically added to Publons, you can then choose whether or not to claim your Publons credit. I understand this statement.

Yes Choose an item.
