# Supplementary material for: VariantSpark: Cloud-based machine learning for association study of complex phenotype and large-scale genomic data
Source: Gigascience. 2020 Aug 6;9(8):giaa077. doi: 10.1093/gigascience/giaa077 (PMC7407261; doi:10.1093/gigascience/giaa077)
Supplement: giaa077_Reviewer_2_Report_Revision_1 — Joshua W. K. Ho, PhD -- 5/12/2020 Reviewed [file giaa077_reviewer_2_report_revision_1.pdf]

Reviewer Report

Title: VariantSpark: Cloud-Based Machine Learning for Association Study of Complex Phenotype and Large-Scale Genomic Data

Version: Revision 1 **Date:** 5/12/2020

Reviewer name: Joshua W. K. Ho, PhD

Reviewer Comments to Author:

The revised manuscript is significantly better than the previous version. This version has been significantly re-written to focus on the application of VariantSpark in genomics, and have added a lot of experimental results to support their claims. It has addressed all my previous concerns.
Nonetheless, there are still quite a few things that require clarification:
1. The title and abstract make it sounds like VariantSpark is a 'general' machine learning framework for genomic data. Am I right in saying that vertical partitioning primarily works in paralleling large genomic data because you are using random forest? If I am correct, then it may be more prudent to say that VariantSpark harness the parallelisable nature of RF to develop a scalable cloud-based method to identify epistatic and polygenic genotype-phenotype associations in very large genomic data.
2. I am hoping the authors can provide a figure to illustrate how vertical partitioning is implemented in the Spark framework, as this seems to be an important technical contribution. Can the authors also comment on how this 'framework' can be extended to other non-RF machine learning methods?
3. The authors mentioned PRS quite a few times in the text. It would be nice if the authors can provide at least one example to show that a PRS that incorporates epistatic gene-gene interactions found by VariantSpark can significant increase the prediction of phenotype based on genotypic data, ideally from real GWAS data.
4. In the abstract, the first sentence in conclusions is problematic. I think you meant to say 'Compared to univariate genotype-phenotype testing performed on GWAS data, VariantSpark better ...'

Level of Interest

Please indicate how interesting you found the manuscript: Choose an item.

Quality of Written English

Please indicate the quality of language in the manuscript: Choose an item.

Declaration of Competing Interests

Please complete a declaration of competing interests, considering the following questions:

- Have you in the past five years received reimbursements, fees, funding, or salary from an organisation that may in any way gain or lose financially from the publication of this manuscript, either now or in the future?
- Do you hold any stocks or shares in an organisation that may in any way gain or lose financially from the publication of this manuscript, either now or in the future?
- Do you hold or are you currently applying for any patents relating to the content of the manuscript?
- Have you received reimbursements, fees, funding, or salary from an organization that holds or has applied for patents relating to the content of the manuscript?
- Do you have any other financial competing interests?
- Do you have any non-financial competing interests in relation to this paper?

If you can answer no to all of the above, write 'I declare that I have no competing interests' below. If your reply is yes to any, please give details below.

I declare that I have no competing interests

I agree to the open peer review policy of the journal. I understand that my name will be included on my report to the authors and, if the manuscript is accepted for publication, my named report including any attachments I upload will be posted on the website along with the authors' responses. I agree for my report to be made available under an Open Access Creative Commons CC-BY license (http://creativecommons.org/licenses/by/4.0/). I understand that any comments which I do not wish to be included in my named report can be included as confidential comments to the editors, which will not be published.

Choose an item.

To further support our reviewers, we have joined with Publons, where you can gain additional credit to further highlight your hard work (see: https://publons.com/journal/530/gigascience). On publication of this paper, your review will be automatically added to Publons, you can then choose whether or not to claim your Publons credit. I understand this statement.

Yes Choose an item.
