# Supplementary material for: VariantSpark: Cloud-based machine learning for association study of complex phenotype and large-scale genomic data
Source: Gigascience. 2020 Aug 6;9(8):giaa077. doi: 10.1093/gigascience/giaa077 (PMC7407261; doi:10.1093/gigascience/giaa077)
Supplement: giaa077_Reviewer_3_Report_Original_Submission — Faraz Faghri -- 11/4/2019 Reviewed [file giaa077_reviewer_3_report_original_submission.pdf]

Reviewer Report

Title: VariantSpark: Cloud-Based Machine Learning for Association Study of Complex Phenotype and Large-Scale Genomic Data

Version: Original Submission **Date:** 11/4/2019

Reviewer name: Faraz Faghri

Reviewer Comments to Author:

Summary and strength
- Many datasets have a large number of features that raise algorithmic and computational challenges. This paper has focused on tackling the high dimensionality problem by focusing on the computational aspect of random forest (RF) algorithms. For developing machine learning based predictive models, the random forest is one of the algorithms which produces high prediction accuracies. This paper has implemented a parallel version of RF on top of Spark. The work is based on YGGDRASIL, a scalable decision tree implementation. Authors contribution: (1) extended YGGDRASIL by adding boosting and mtry, (2) parallelization in the tree growing process such that nodes of different trees are processed in parallel.
- Authors have made VariantSpark publicly available.
- In the results, authors have provided explanation/intuition of why Variant Spark performs better. Which seems to the parallelization algorithm and less about certain coding/optimization techniques. The algorithmic contribution makes their work more generalizable for future systems.
Issues
- There seems to be a major issue in benchmarking and comparison. Authors are extending YGGDRASIL but there is no performance comparison to YGGDRASIL. Table 1 shows they are going to compare but Results and Figure 1 do not include any comparison to YGGDRASIL.
Other comments
- "Ultra high dimensional" is not well-defined, I suggest just using "high dimensional"
- Keywords: machine learning and developing predictive models is not part of traditional GWAS. I suggest mentioning the polygenic risk score
- Introduction/background section references are irrelevant papers.
  - to solution papers [2] and [3]. They should reference survey/review type papers that have generated the data.
  - [4] reference is on the non-high dimension data. It is a 12-year-old GWAS that used Affymetrix GeneChip 500K Mapping Array Set (500,568 features). Better reference a recent large deep whole-genome sequencing analysis, for instance: https://www.pnas.org/content/113/42/11901.short
  - [5] should be: https://www.nature.com/articles/s41576-019-0122-6
- "…all of these implementations are optimized for a high- performance computing (HPC) machine (a single computing node)." This seems a false statement. For instance, H2O has a distributed RF which is designed for distributed multi-machine computation http://docs.h2o.ai/h2o/latest-stable/h2o-docs/data-science/drf.html

- In practice, we are seeing that xgboost implementation of decision trees is currently producing the highest accuracy for genomic data. It would be great to show how this work compares to xgboost, since xgboost also has a distributed implementation. Not necessary for this paper but a nice addition for future work maybe.

Level of Interest

Please indicate how interesting you found the manuscript: Choose an item.

Quality of Written English

Please indicate the quality of language in the manuscript: Choose an item.

Declaration of Competing Interests

Please complete a declaration of competing interests, considering the following questions:

- Have you in the past five years received reimbursements, fees, funding, or salary from an organisation that may in any way gain or lose financially from the publication of this manuscript, either now or in the future?
- Do you hold any stocks or shares in an organisation that may in any way gain or lose financially from the publication of this manuscript, either now or in the future?
- Do you hold or are you currently applying for any patents relating to the content of the manuscript?
- Have you received reimbursements, fees, funding, or salary from an organization that holds or has applied for patents relating to the content of the manuscript?
- Do you have any other financial competing interests?
- Do you have any non-financial competing interests in relation to this paper?

If you can answer no to all of the above, write 'I declare that I have no competing interests' below. If your reply is yes to any, please give details below.

I declare that I have no competing interests

I agree to the open peer review policy of the journal. I understand that my name will be included on my report to the authors and, if the manuscript is accepted for publication, my named report including any attachments I upload will be posted on the website along with the authors' responses. I agree for my report to be made available under an Open Access Creative Commons CC-BY license (http://creativecommons.org/licenses/by/4.0/). I understand that any comments which I do not wish to be included in my named report can be included as confidential comments to the editors, which will not be published.

Choose an item.

To further support our reviewers, we have joined with Publons, where you can gain additional credit to further highlight your hard work (see: https://publons.com/journal/530/gigascience). On publication of this paper, your review will be automatically added to Publons, you can then choose whether or not to claim your Publons credit. I understand this statement.

Yes Choose an item.
